# Profiling of runs of homozygosity from whole-genome sequence data in Japanese biobank

**DOI:** 10.1038/s10038-025-01331-3

**Published:** 2025-04-03

**Authors:** Aye Ko Ko Minn, Motomichi Matsuzaki, Akira Narita, Takamitsu Funayama, Yurii Kotsar, Satoshi Makino, Jun Takayama, Tohoku Medical Megabank Project Study Group, Akira Narita, Satoshi Makino, Hikaru Abe, Michiaki Abe, Momoka Abe, Naomi Abe, Noriko Abe, Tomomi Abe, Yuto Abe, Shizuko Ahiko, Kayo Aiki, Hiromi Aizawa, Yukari Akiyama, Hayato Anzawa, Eri Aoki, Yuichi Aoki, Hiroko Arai, Misaki Arakawa, Yukie Asano, Liam Baird, Ayano Chiba, Haruna Chiba, Ippei Chiba, Kenji Chiba, Keiko Chida, Inaho Danjoh, Hisako Endo, Reika Fue, Futaba Fujishiro, Yayoi Fujita, Waka Fukunaga, Takuo Fukushi, Mami Funata, Takamitsu Funayama, Sho Furuhashi, Nobuo Fuse, Kumiko Fushiya, Tomomi Gamo, Chinatsu Gocho, Katsuhiro Gonoi, Maki Goto, Takahiko Goto, Yukie Goto, Kaori Gouko, Michiko Haga, Yoko Haga, Yuko Hamada, Yohei Hamanaka, Mika Hanazawa, Yukari Hara, Hisano Hasebe, Atsushi Hasegawa, Hiroaki Hashizume, Asuka Hatakeyama, Sumika Hatakeyama, Nozomi Hatanaka, Rieko Hatanaka, Takanori Hidaka, Kenji Hino, Hiroe Hirama, Ikuo Hirano, Sachiko Hirano, Takumi Hirata, Masahiro Hiratsuka, Yuki Hiratsuka, Ikuko Hirayama, Eiji Hishinuma, Atsushi Hozawa, Keisuke Ido, Nobuko Igari, Chikako Iida, Katsuko Imai, Makiko Inoue, Marie Inoue, Reiko Inoue, Rumi Irie, Motoko Ishida, Noriko Ishida, Eri Ishigaka, Chihiro Ishii, Osamu Ishii, Tadashi Ishii, Tatsuro Ishikawa, Mami Ishikuro, Kazutoshi Ishimori, Ryosuke Ishiwata, Miho Itabashi, Maiko Ito, Masumi Ito, Mayumi Ito, Rie Ito, Saori Ito, Fumihiko Iwabuchi, Maki Iwabuchi, Yoko Izumi, Yoshiko Izumi, Masataka Kambe, Kanako Watanabe, Takanari Kanno, Mayu Kano, Naoko Kasahara, Hinako Kashiwa, Kiyomi Katahira, Mayumi Kato, Yukie Kato, Fumiki Katsuoka, Takeshi Kawabata, Rika Kawada, Aoi Kawagoe, Hiroshi Kawame, Junko Kawashima, Yukako Kawashima, Junko Kikuchi, Masahiro Kikuya, Masae Kimura, Kengo Kinoshita, Ikuko Kishi, Tomoko Kishimoto, Tamie Kitaura, Mika Kobayashi, Tadao Kobayashi, Tomoko Kobayashi, Eiichi N. Kodama, Shun Kodate, Mana Kogure, Toshisada Kohagizawa, Naomi Kohketsu, Noa Koida, Chie Koide, Mika Koide, Toshihiko Koike, Shohei Koiso, Kaname Kojima, Junko Komatsu, Ayumi Kondo, Yukie Konno, Sachie Koreeda, Seizo Koshiba, Takuya Koyama, Hisaaki Kudo, Kazuki Kumada, Ryoko Kumadaki, Rika Kumagai, Toshie Kumagai, Yuko Kumagai, Yasuto Kunii, Miho Kuriki, Shinichi Kuriyama, Miyuki Kuroda, Emiko Kurokawa, Seiko Kurota, Hisako Kusano, Bin Li, Donghan Li, Kanako Maeshibu, Keiko Maeta, Hiroko Matsubara, Naomi Matsukawa, Masako Matsumoto, Takako Matsuoka, Yuka Matsushita, Fumiko Matsuzaki, Motomichi Matsuzaki, Hirohito Metoki, Sayaka Minakawa, Yuki Minami, Kyoko Mitate, Satomi Mito, Ayako Miura, Noriko Miura, Ryo Miyagi, Akiko Miyazawa, Satoshi Mizuno, Akiko Mochida, Mika Momii, Hiroko Mori, Naoko Mori, Hozumi Motohashi, Ikuko N. Motoike, Shunji Mugikura, Keiko Murakami, Takahisa Murakami, Toshiro Muranishi, Masato Nagai, Satoshi Nagaie, Fuji Nagami, Tatsuo Nagasaka, Sachiko Nagase, Kumiko Nakagawa, Taku Nakai, Noriko Nakajo, Naoki Nakamura, Tomohiro Nakamura, Yuko Nakasato, Kumi Nakaya, Naoki Nakaya, Kei Nanatani, Natsuko Narisawa, Yuka Narita, Hafumi Nishi, Kohji Nishida, Ichiko Nishijima, Takahiro Nobukuni, Kotaro Nochioka, Aoi Noda, Kenichi Noguchi, Kiriko Nozoe, Rie Nunokawa, Taku Obara, Tomoko Obara, Kaori Ogasawara, Satoru Ogawa, Soichi Ogishima, Nahoko Ohi, Namiko Ohisa, Kinuko Ohneda, Hayami Ohori, Yumi Oikawa, Yumiko Ojima, Yumi Okada, Yasunobu Okamura, Hiroshi Okuda, Mitsuko Okuda, Ayako Okumoto, Akane Ono, Chiaki Ono, Genki Onodera, Kaname Onodera, Masako Onodera, Midori Onuma, Tomomi Onuma, Keiichiro Oohashi, Masumi Oomachi, Kazuya Ootomo, Yukie Oouchi, Kazuko Oowada, Masatsugu Orui, Mayumi Osada, Tamae Osanai, Reiko Ota, Noriko Otake, Sumie Otomo, Tatsui Otsuka, Akihito Otsuki, Yoko Otsuki, Yuki Oyama, Keiko Oyamada, Masahiro Ozawa, Yoko Ozawa, Satomi Obara, Daisuke Saigusa, Asami Saito, Asuka Saito, Hisako Saito, Kazue Saito, Manami Saito, Megumi Saito, Ritsumi Saito, Sakae Saito, Tomo Saito, Yoshinobu Saitoh, Hiroko Sakai, Masaki Sakaida, Hiroshi Sakamono, Hiromi Sakamoto, Kana Sakamoto, Mia Sakamoto, Kasumi Sakurai, Miyuki Sakurai, Rieko Sakurai, Mika Sakurai-Yageta, Eriko Sasaki, Kana Sasaki, Miho Sasaki, Tadashi Sasaki, Yukari Sasaki, Yukie Sasaki, Akemi Sato, Chika Sato, Hirokazu Sato, Mayumi Sato, Michiyo Sato, Miho Sato, Mitsuharu Sato, Miu Sato, Naoko Sato, Reiko Sato, Satoshi Sato, Shiho Sato, Taku Sato, Yoshiko Sato, Youko Sato, Yui Sato, Yuriko Sato, Michihiro Satoh, Ayako Sekiya, Koji Shibuya, Hirohito Shima, Yoshiko Shima, Muneaki Shimada, Atsushi Shimizu, Ritsuko Shimizu, Genki Shinoda, Nobuyuki Shirakawa, Matsuyuki Shirota, Hiroe Shoji, Ikuko Shoji, Mariko Shoji, Midori Shoji, Wakako Shoji, Satomi Someya, Shinya Sonobe, Itsumi Sou, Rie Suenaga, Yasuko Suenaga, Mayumi Suga, Rika Sugai, Junichi Sugawara, Megumi Sugawara, Michiko Sugawara, Nanako Sugawara, Saori Sugawara, Yuki Sugawara, Sachiyo Sugimoto, Yoshiko Suto, Airi Suzuki, Ayano Suzuki, Keiko P. Suzuki, Mariko Suzuki, Michirou Suzuki, Mikiko Suzuki, Norio Suzuki, Rie Suzuki, Ryoko Suzuki, Takafumi Suzuki, Tatsuya Suzuki, Yoichi Suzuki, Kaho Sato, Shu Tadaka, Keiko Taguchi, Nozomi Taiji, Makiko Taira, Kaori Takagi, Emi Takahashi, Harumi Takahashi, Junko Takahashi, Megumi Takahashi, Noriko Takahashi, Rieko Takahashi, Yukiko Takahashi, Mayuko Takasawa, Jun Takayama, Miho Takeuchi, Yoshinobu Takeyama, Sayaka Takita, Toru Tamahara, Gen Tamiya, Naomi Tamura, Akari Tanaka, Saiko Tanaka, Chihiro Tanno, Naoko Tanno, Keiko Tateno, Minoru Tateno, Chika Terui, Yuriko Tezuka, Mihoko Toki, Etsuko Tomita, Hiroaki Tomita, Mai Tomizuka, Akiko Toriyama, Naho Tsuchiya, Miyuki Tsuda, Tomomi Tsumuraya, Junko Tsunasawa, Issei Tsunoda, Juri Uchiya, Akiko Ueda, Yuriko Ueki, Fumihiko Ueno, Rumi Ujiie, Keiko Umeda, Akira Uruno, Ikuko Wada, Tomoko Wada, Mika Wagatsuma, Hitoshi Watanabe, Kazue Watanabe, Nobuo Yaegashi, Mika Yagyu, Etsuko Yamada, Yumi Yamaguchi-Kabata, Masayuki Yamamoto, Tomiko Yamauchi, Yukari Yamauchi, Mika Yamazaki, Kenji Yano, Jun Yasuda, Hang Yin, Hiroshi Yokota, Manami Yokoyama, Yuko Yoshida, Mizue Yoshino, Zhiqian Yu, Yoshiyuki Yukawa, Lin Zhang, Makoto Sasaki, Akimune Fukushima, Yasushi Ishigaki, Atsushi Shimizu, Koichi Asahi, Ryoichi Tanaka, Kozo Tanno, Kotaro Otsuka, Fumie Aizawa, Naoyuki Nishiya, Mitsuko Iwabuchi, Fumitaka Tanaka, Shinichi Omama, Kouhei Hashizume, Noriko Takebe, Kazuhiro Yoshikawa, Yuka Kotozaki, Masato Nagai, Takahiro Mikami, Takahito Nasu, Junko Akai, Yorihiro Koeda, Yohei Sawa, Nobuyuki Takanashi, Yayoi Yamasaki, Haruki Terui, Kasumi Hannokizawa, Hideki Ohmomo, Shohei Komaki, Mamoru Satoh, Yoichi Sutoh, Fumio Yamashita, Yutaka Hasegawa, Shiori Minabe, Tsuyoshi Hachiya, Tomoharu Tokutomi, Yukiko Toya, Akiko Yoshida, Satoshi Nishizuka, Ryujin Endo, Shinichi Kuriyama, Gen Tamiya

**Affiliations:** 1 Department of AI and Innovative Medicine, Graduate School of Medicine, Tohoku University, Sendai, Japan; 2 RIKEN Center for Advanced Intelligence Project, Tokyo, Japan; 3 Tohoku Medical Megabank Organization, Tohoku University, Sendai, Japan; 4 Mathematical Intelligence for Medicine, Graduate School of Medicine, Tohoku University, Sendai, Japan; 5 International Research Institute of Disaster Science, Tohoku University, Sendai, Japan; 6 Iwate Tohoku Medical Megabank Organization, Iwate Medical University, Iwate, Japan

**Keywords:** Computational biology and bioinformatics, Genomics, Population genetics

## Abstract

Runs of homozygosity (ROHs) are widely observed across the genomes of various species and have been reported to be associated with many traits and common diseases, as well as rare recessive diseases, in human populations. Although single nucleotide polymorphism (SNP) array data have been used in previous studies on ROHs, recent advances in whole-genome sequencing (WGS) technologies and the development of nationwide cohorts/biobanks are making high-density genomic data increasingly available, and it is consequently becoming more feasible to detect ROHs at higher resolution. In the study, we searched for ROHs in two high-coverage WGS datasets from 3552 Japanese individuals and 192 three-generation families (consisting of 1120 family members) in prospective genomic cohorts. The results showed that a considerable number of ROHs, especially short ones that may have remained undetected in conventionally used SNP-array data, can be detected in the WGS data. By filtering out sequencing errors and leveraging pedigree information, longer ROHs are more likely to be detected in WGS data than in SNP-array data. Additionally, we identified gene families within ROH islands that are associated with enriched pathways related to sensory perception of taste and odors, suggesting potential signatures of selection in these key genomic regions.

## Introduction

Runs of homozygosity (ROH), stretches of continuously homozygous segments in genomes, are widely observed in various species, including humans. The degree of homozygosity within individual genomes can be altered by a combination of factors, including population history, which is characterized by migration patterns, demographic shifts, population bottlenecks, and cultural practices such as endogamy or consanguinity. Japanese population is known for its unique genetic background, shaped by geographic isolation and limited gene flow, which makes it particularly relevant for the assessment of genetic homogeneity and the detection of both distant and recent inbreeding. Previous studies demonstrated that modern Japanese and ancient Jomon individuals exhibit a relatively high average total length of ROH, especially in shorter categories (≤500 KB) [1–3]. However, since ROH analyses in the modern Japanese have so far been conducted on a small scale with low-coverage whole genome sequencing (WGS) and array data, there remains a lack of comprehensive insights into ROH patterns based on large-scale, high-coverage data in the Japanese population.

Currently available methods for estimating homozygosity level have been advanced during the past decades. Traditionally, the inbreeding coefficient [4], *F*, was used to estimate the proportion of the homozygous segments from pedigree information [5]. Microsatellites, known as short tandem repeats (STRs), have been also used to directly seek the consecutive homozygous genotypes [6]. Subsequently, significant advancements in SNP-genotyping microarray technologies allowed researchers to interrogate genomic regions with an elevated degree of homozygosity with higher accuracy [2]. In former years, most studies have focused on megabase-scale ROHs due to their association with inbreeding. However, ROHs can affect human traits and diseases beyond the context of inbreeding, making it important to consider shorter ROHs. Accordingly, shorter ROHs have been extensively studied in recent years, but in such cases, it was questionable whether SNP-array data, even if it is high-density, can detect very short ROHs accurately [3]. However, with the emergence of high-throughput next-generation sequencing (NGS) technologies, the detectability of ROHs has been continuously increasing, thereby allowing for the identification of shorter ROHs to a greater extent.

ROH segments in the genome can be investigated by different ROH detection tools, each based on a different approach. PLINK [7] scans chromosomes for consecutive homozygous genotypes by sliding a fixed-size window of detection, and an ROH is called if the count of consecutive homozygous SNPs satisfies the predefined condition. However, its algorithms were initially designed for SNP genotyping array data, and hence, it is necessary to adjust some of its parameters when applied to other data types [3, 8]. Alternatively, several model-based programs, including Beagle, H^3^M^2^, and BCFtools, which are employing hidden Markov models (HMM), can identify potential sequences of homozygosity [9–11]. Among the available tools, PLINK has been extensively used in numerous ROH studies [12–14], providing a convenient mean of comparing SNP-array outputs across different study groups. However, a prior study by Narasimhan et al. [11] asserted that BCFtools can be applied in sequencing data with better accuracy.

An obvious concern is that NGS may introduce sequencing errors [15, 16], which could potentially affect the accuracy of ROH identification [3]. The precise origins of such erroneous calls have been widely studied, indicating that they can arise at any stages of the sequencing process, ranging from sample handling and genomic library preparation to intrinsic errors of sequencing platforms [17, 18]. Some sequencing errors could be observed as Mendelian errors if pedigrees are known. Error correction processes such as removing sequencing errors which are not consistent with Mendelian inheritance would be effective for the identification of ROH. The impact of sequencing errors on ROH should not be neglected, yet only few studies address this issue. The accuracy of ROH identification can be enhanced to a certain extent by leveraging the extensively available pedigree data and removing sequencing errors through scanning Mendelian inconsistencies.

One of the surprising attributes of ROH is its vast degrees of variability in distributions across genomic regions, reflecting diverse patterns of inheritance, recombination, and population structure. Indeed, Ceballos et al. have shown that ROHs are not uniformly distributed across the human genome. Instead, ROHs tend to cluster within the specific genomic regions, forming ROH islands, the genomic locations of which can vary depending on ethnicities or genetic backgrounds [19]. Understanding the biological pathways of genes within ROH islands can provide comprehensive insights into the broader functional significance of these regions.

In this study, we applied two high-coverage whole-genome sequencing datasets: 3.5KJPNv2—a dataset constructed from a haplotype frequency panel of 3552 Japanese individuals [20], and BirThree—a dataset along with pedigree information of 1120 Japanese individuals, derived from Birth and Three-generation Cohort Study [21], which can allow us to more precisely identify shorter ROH segments by taking into account the effects of sequencing errors.

To illustrate the large advantages provided by WGS datasets, we investigated the detectability of ROHs down to very low minimum length, at the kilobase level, in the Japanese population. To further validate the detection of ROHs, we assessed the impact of SNP density by comparing all variant sites as well as trimmed SNP array-based sites in each dataset. We also evaluated the effects of sequencing errors on detection of ROHs by leveraging pedigree information. Genomic distribution of ROH segments and their functional impacts were also explored by identifying the ROH islands and pathways enriched within these regions.

## Results

We investigated the detectability of ROH under different analytical approaches and assessed the SNP-density and functional effects, using biobank-level scale Japanese WGS datasets [20–23] and major bioinformatic tools [7, 11]. We showed that whole genome sequencing uncovers very short ROH segments that genotyping arrays fail to detect. Although long ROH segments may be affected by sequencing errors, integrating pedigree-based quality control into WGS data can help counteract these inaccuracies. Furthermore, our results revealed that using WGS data that incorporated pedigree information substantially enhances functional pathway enrichment within ROH islands.

### The total numbers of ROH segments

**Fig. 1:**
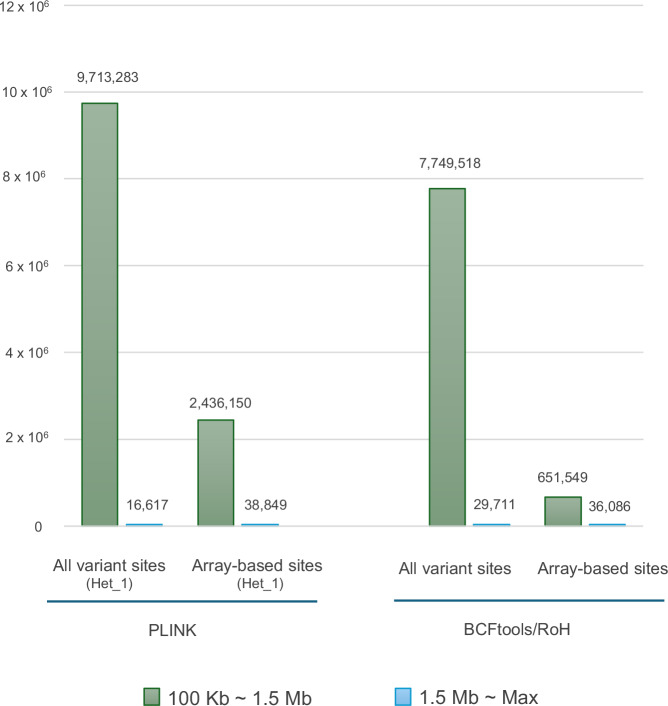
Distribution of total numbers of ROHs in 3.5KJPNv2 dataset among all individuals. Bar graphs represent genome-wide all variant sites and OmniExpressExome array-based sites specific ROH distribution in 3.5KJPNv2 dataset based on the selected tools. “Het_1” denotes the use of default value 1 in PLINK “--homozyg-window-het”. Color scheme represents ROH segments length intervals: ROHs between 100 Kb and 1.5 Mb, and ROHs above 1.5 Mb

First, we performed an analysis on 3.5KJPNv2 dataset to investigate the effects of marker density. We have compared length intervals of detected ROH, and for both BCFtools and PLINK, we observed a higher prevalence of ROHs between 100 KB and 1.5 Mb at all variant sites compared to the array-based sites (Fig. 1 and S1A–B). However, we noticed that longer ROHs (>1.5 Mb) are more abundant in array-based sites compared to all variant sites in both tools (Fig. 1).

Next, we further investigated the mean number (NROH) and the mean cumulative sums (SROH) of ROH segments per individual. In this context, ROH minimal length thresholds were set starting from 100 Kb for shorter ROHs (i.e., ROH_100_, NROH_100_ and SROH_100_), and from 1.5 Mb for longer ROHs (i.e., ROH_1500_, NROH_1500_ and SROH_1500_) to facilitate comparison with previous research [12, 13, 24].

### Mean number of ROH segments (NROH) and mean cumulative sums of ROH segments (SROH)

#### ROH > 1.5 Mb (3.5KJPNv2 dataset)

**Fig. 2:**
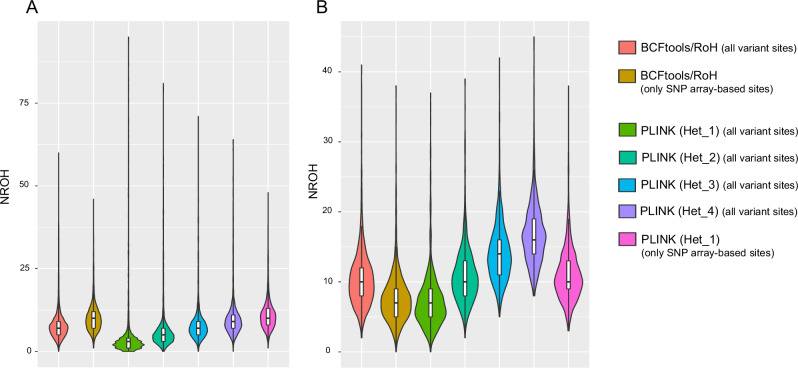
Distribution of mean number of ROH_1500_ (NROH) per individual in (**A**) 3.5KJPNv2 dataset and (**B**) BirThree dataset. Violin plot represents the distribution of mean number of ROH segments longer than 1.5 Mb across individuals in the 3.5KJPNv2 and BirThree dataset. Color schemes represent specific conditions: genomic regions and parameter adjustments in selected tools. PLINK “--homozyg-window-het” option values were set to a range of 1–4, i.e., allowing from one to four heterozygous calls per window. These are abbreviated as “Het_1”, “Het_2”, “Het_3”, and “Het_4”, respectively

We set a minimal ROH length of 1.5 Mb to detect longer ROHs per-individual and investigated the NROH and SROH accordingly. We detected a consistent pattern, as seen in analysis of total numbers of ROH segments, where ROH_1500_ are more prevalent in the array-specific regions than in all variant sites by using both BCFtools and PLINK (Table 1, Fig. 2A). The default value for the PLINK parameter (--homozyg-window-het 1), which allows one heterozygous call per window, has been widely used in previous array-based studies on longer ROHs [3, 12, 13]. We applied this conventional parameter value to facilitate direct comparisons with previous research. Our results demonstrated strong concordance between results obtained using PLINK (mean NROH_1500_ of 10.94) and BCFtools (mean NROH_1500_ of 10.16) in detecting ROH_1500_ within array-based sites (Table 1, Fig. 2A).

**Table 1:** Distribution of Mean of total number of ROH (NROH) and total sums of ROH (SROH) per individual in 3.5KJPNv2 dataset after implementing minimal ROH length threshold of 100 Kb, and 1.5 Mb, respectively

Tools	Regions and parameter options	ROH metrics	Mean values for different minimal ROH lengths	
			>100 Kb	>1.5 Mb
**BCFtools**	All variant sites	NROH	2190	8.37
		SROH	582,285,742	23,464,275
	Only SNP array-based sites^*a^	NROH	194	10.16
		SROH	127,461,992	29,879,304
**PLINK**	All variant sites/Het_1	NROH	2739	4.68
		SROH	594,581,242	12,701,591
	All variant sites/Het_2	NROH	3083	6.59
		SROH	720,861,960	17,901,917
	All variant sites/Het_3	NROH	3302	8.53
		SROH	800,370,778	22,679,954
	All variant sites/Het_4	NROH	3471	10.06
		SROH	858,847,627	26,260,213
	Only SNP array-based sites/Het_1^*a^	NROH	697	10.94
		SROH	318,169,943	30,192,850

PLINK “--homozyg-window-het” option values were set to a range of 1 to 4, i.e., allowing from one to four heterozygous calls per window. These are abbreviated as “Het_1”, “Het_2”, “Het_3”, and “Het_4”, respectively

*^a^ Although the SNP array data is unavailable, we have effectively trimmed whole genome sequencing (WGS) data to restrict our analysis exclusively on the regions specified by OmniExpressExome Array

However, longer ROHs tend to be less common in highly dense variant regions than in sparse array sites. This is likely due to sequencing errors, which increase the probability of interruptions in consecutive homozygous genotypes. Ceballos et al. [3] suggested that aforementioned PLINK parameter value can be adjusted to 3 or 4 to allow for more heterozygous calls in WGS data to achieve comparability with array-based data. Therefore, we adjusted PLINK to allow specific number of heterozygous calls per window to account for the impact of sequencing errors in all variant sites, ensuring a reliable comparison with array-based sites. To match with the results observed in SNP array-based sites (mean NROH_1500_ of 10.94), four heterozygous calls were required to be allowed per window in all variant sites (mean NROH_1500_ of 10.06), which is in agreement with Ceballos et al.’s previous findings [3].

Indeed, when comparing the results between BCFtools and PLINK with adjusted parameters, we observed mean NROH_1500_ of 8.53 and SROH_1500_ of 22.7 Mbp for “Het_3” in PLINK, which can be comparable with findings for BCFtools (mean NROH_1500_ of 8.37 and SROH_1500_ of 23.5 Mbp). This result enables us to estimate the influence of sequencing errors in BCFtools detection (Table 1, Fig. 2A).

#### ROH > 1.5 Mb (BirThree dataset)

We next analyzed the dataset from the Tohoku Medical Megabank (TMM) Project BirThree Cohort (Table 2, Fig. 2B). In contrast to the 3.5KJPNv2 dataset, BCFtools detected more ROH_1500_ segments across all variant sites (mean NROH_1500_ of 10.44 and SROH_1500_ of 27.5 Mbp) than in the array-specific regions of the BirThree dataset. The heterozygous calls allowed per window were reduced to two when compared with PLINK across all variant sites (mean NROH_1500_ of 10.88 and SROH_1500_ of 29.1 Mbp), indicating greater robustness against sequencing errors in this dataset (Table 2, Fig. 2B).

**Table 2:** Distribution of Mean of total number of ROH (NROH) and total sums of ROH (SROH) per individual in BirThree dataset after implementing minimal ROH length threshold of 100 Kb, and 1.5 Mb, respectively

Tools	Regions and parameter options	ROH metrics	Mean values for different minimal ROH lengths	
			>100 Kb	>1.5 Mb
**BCFtools**	All variant sites	NROH	1713	10.44
		SROH	505,931,167	27,486,031
	Only SNP array-based sites^*a^	NROH	67	7.71
		SROH	61,110,307	22,104,265
**PLINK**	All variant sites/Het_1	NROH	2726	7.18
		SROH	627,622,136	20,846,395
	All variant sites/Het_2	NROH	3072	10.88
		SROH	758,579,183	29,116,852
	All variant sites/Het_3	NROH	3324	14.16
		SROH	848,691,286	36,505,896
	All variant sites/Het_4	NROH	3532	16.82
		SROH	919,263,432	42,598,045
	Only SNP array-basedsites/Het_1^*a^	NROH	357	11.06
		SROH	206,508,058	27,499,818

PLINK “--homozyg-window-het” option values were set to a range of 1–4, i.e., allowing from one to four heterozygous calls per window. These are abbreviated as “Het_1”, “Het_2”, “Het_3”, and “Het_4”, respectively

*^a^ Although the SNP array data is unavailable, we have effectively trimmed whole genome sequencing (WGS) data to restrict our analysis exclusively on the regions specified by OmniExpressExome Array

A comprehensive evaluation of ROH segments, comparing distributions of numerous ROH lengths between all variant sites and array specific sites showed smaller dissimilarities in the BirThree dataset (Fig. S4E–H).

#### ROH > 100 Kb

To further investigate these results, we have run BCFtooIs on ROH_100_ segments within all variant sites in BirThree dataset. As listed in Tables 1, 2, a significantly reduced number of ROH (mean NROH_100_ of 1713 and SROH_100_ of 505.9 Mbp) were detected in BirThree dataset compared to the 3.5KJPNv2 dataset (NROH_100_ of 2,190 NROH and SROH_100_ of 582.3 Mbp). Intriguingly, depending on the minimal lengths of ROH, we observed an opposite tendency in both datasets (Fig. S3A). Using PLINK instead, we have not observed significant differences between two datasets (Tables 1, 2).

To exclude the effects of SNP density, we randomly selected the same number of SNPs from the 3.5KJPNv2 dataset as in the BirThree dataset. We utilized BCFtools on the pruned 3.5KJPNv2 dataset again, applying the same differentiation methods for minimal ROH lengths as described above. Increased numbers of longer ROHs were observed when comparing BirThree (mean NROH_1500_ of 10.44) and the pruned 3.5KJPNv2 (mean NROH_1500_ of 8.82) datasets, while no such significant change was noted when comparing the pruned 3.5KJPNv2 and its unfiltered version (Table 3 and Fig. S3A, left). Unlike ROH_1500_, we observed a noticeable declining pattern in the detection of ROH_100_ in both comparisons, indicating the possible influence of SNP density, upon adjusting minimal length to 100 Kb (Table 3 and Fig. S3A, right). However, we did not observe significant differences in both ROH_100_ and ROH_1500_ when conducting analysis on only overlapped samples (Fig. S3B–E).

**Table 3:** Assessing the effects of SNP density after SNP-pruning on 3.5KJPNv2 dataset

Tools (Regions)	Minimal ROH length	Mean NROH per individual in different datasets		
		3.5KJPNv2	Pruned 3.5KJPNv2	BirThree
**BCFtools (All variant sites)**	>100 Kb	2190	1976	1713
	>1.5 Mb	8.37	8.82	10.44

To create pruned 3.5KJPNv2 dataset, the same number of SNPs were randomly selected from the 3.5KJPNv2 dataset as in the BirThree dataset for ten iterations

### ROH islands and functional analysis

**Fig. 3:**
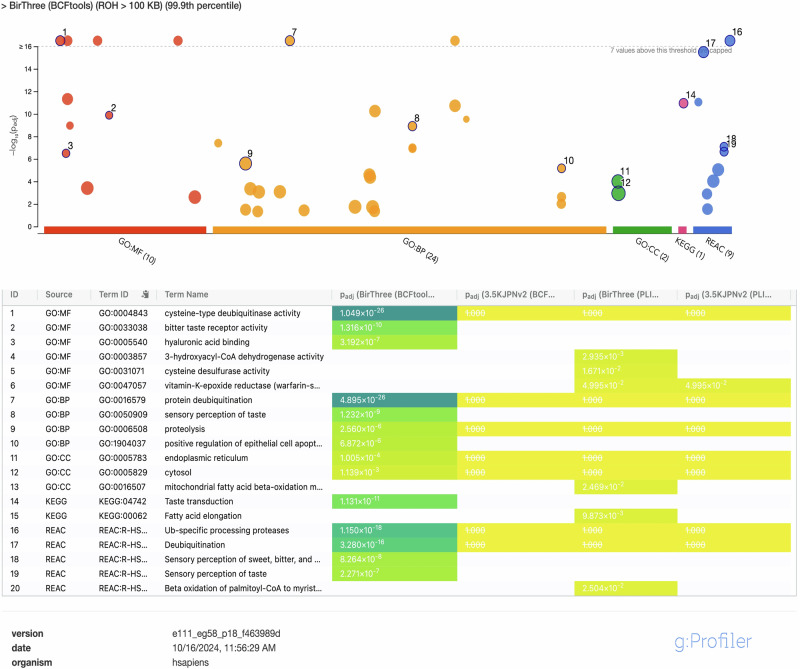
Functional enrichment analysis of annotated genes within runs of homozygosity (ROH) Islands. This figure presents the results of functional enrichment analysis on genes identified within ROH islands located in shorter ROH regions (>100 KB), detected in the BirThree dataset via BCFtools (by setting 99.9^th^ percentile threshold based on the frequencies of overlapping ROH_100_ regions shared among individuals). The gProfiler tool was utilized to identify enriched biological pathways (BP), molecular functions (MF), and cellular components (CC) from Gene Ontology (GO), KEGG, and Reactome. The y-axis displays the enrichment score, indicating statistical significance, while the x-axis and color coding represent the data source. Dot size corresponds to the number of genes associated with each term. A summary of comparative statistics with other dataset (3.5KJPNv2) and tool (PLINK) is also provided. Full figures and statistics related to all analysis can be found in Supplementary Figs. S2A–B and Tables S2A–F

To examine the specific genomic distribution of ROH segments and their functional impacts, we defined ROH islands by applying 99.9th or 99.5th percentile thresholds for the regions with shared ROH segments, depending on the minimum ROH length (Fig. S1C–G). Our analysis revealed enriched pathways related to three main gene families that are prominently involved in ROH island regions detected by BCFtools. First, the USP17 family of genes showed significant presence in pathways related to protein deubiquitination, proteolysis, and cell apoptosis. Second, several members of the TAS2R family (TAS2R14, TAS2R20, TAS2R30, TAS2R31, TAS2R43, TAS2R46, TAS2R50) were identified within ROH islands on chromosome 12 in the BirThree dataset and these were associated with taste receptor activity, taste transduction, and sensory perception of taste pathways (Fig. 3 and S2A, Table S2A). Significant enrichment of olfactory receptor activity, signaling, and transduction pathways was driven by multiple OR4 genes (OR4A47, OR4B1, OR4C3, OR4C5, OR4S1, OR4X1, OR4X2) detected within ROH islands located in longer ROH regions (>1.5 MB) (Fig. S2B and Table S2D–E). Additionally, ROH islands located in regions containing genes such as HADHA, HADHB, IP6K1, and IP6K2, which are involved in enzymatic activity linked to fatty acid metabolism and inositol phosphate synthesis were also identified by PLINK (Fig. 3 and S2A–B) (Tables S2B–C and F).

Additionally, we also examined the genomic-based inbreeding coefficient (F_ROH_) to unravel the validity of our results, using the same method as previously described [13]. By doing this, we obtained the array-based sites result (F_ROH > 1.5 MB_ = 0.010064) consistent with that of a previous Clark et al. study, specifically for Japanese population (Table S1G). Similarly, upon stratification analysis, we validated a marked reduction in the number of very short ROH segments (200–500 KB) in array-based regions compared to all variant sites across both tools (Fig. S4A–H).

Next, we identified the common sites where Mendelian-inconsistent calls occurred in the BirThree dataset and then analyzed a subset of the 3.5KJPNv2 dataset with these sites excluded. We also altered the genetic map to the 1000 Genomes Project (1KGP) in BCFtools analysis. However, these latter two analyses did not provide significant differences (Table S1A–D).

## Discussion

ROHs are distributed over a wide range of genomic regions in many species. Modern humans have substantially lower genetic diversity, i.e., an estimated effective population size of only about 10,000, compared with other species, and are thus expected to have many ROHs. In this study, we demonstrated that WGS data with a heightened SNP density in widespread genome regions can call many shorter ROH segments compared to array-specific regions. In addition, having used the BirThree dataset, we observed that leveraging pedigree information can mitigate sequencing error effects, especially on longer ROH segments, regardless of SNP-density effects and also demonstrate strong functional enrichment.

Initially, we performed ROH segment identification in the 3.5KJPNv2 dataset using PLINK and BCFtools. Our results demonstrate that a greater number of shorter ROH segments can be detectable in all variant sites compared to only array-specific sites, suggesting that WGS technology can enhance the detection of ROHs in many unexplored regions that cannot be identified by using array-based technologies. Previous studies indicated that the density of genetic variants and the quality of genotype calling may affect the detection of true ROHs [12, 25]. Array genotyping has been extensively used in numerous ROH studies [2, 5]. However, it should be applied with caution when predicting ROH segments, as SNP arrays yield sparse data consisting of a few million autosomal nucleotide positions at most.

We then extended the minimal length of ROH to 1.5 Mb to examine the similar impact in longer ROHs. In contrast, using BCFtools, we first noticed that the capability of detecting ROH_1500_ is reduced in all variant sites of the genome compared to array-specific sites. As an additional benchmark, we utilized PLINK to evaluate its concordance with the NROH and SROH results obtained through BCFtools, by adjusting its parameters. We allowed four heterozygous calls per window across all variant sites, to reach a close similarity in the results with the analysis of only array-specific sites. These results are nearly consistent with the findings in the previous study, which recommended setting the parameter to allow more than three heterozygous calls per window to achieve equivalent results between low-coverage WGS data and array data for a Japanese population [3]. Due to array data usage along with PLINK in previous studies, PLINK’s array-sites results can be considered as the most standard findings when detecting longer ROHs. This also allows us to compare our findings with previous studies [3, 12, 13, 24]. ROH_1500_ detected within array-specific sites between PLINK and BCFtools are comparable in our study, thus further supporting that BCFtools can achieve similar accuracy. However, it is expected that the involvement of sequencing errors in the 3.5KJPNv2 dataset may disrupt long ROHs, thereby reducing the ROH_1500_ detected by BCFtools in all variant sites. Moreover, such possibility of error presence can be corroborated by allowing three heterozygous calls per window by a parameter adjustment in PLINK.

Notwithstanding that the efficiency and effectiveness of WGS technologies are remarkable, applying the technologies in unrelated individuals may result in relatively high probability of error rates [26]. For that reason, we have analyzed another WGS dataset derived from the TMM BirThree Cohort Study, and which includes family-tree information that encompassed fathers, mothers, grandparents and children [21, 23]. Using BCFtools on the BirThree dataset, this time, more ROH_1500_ were identified from all the variant sites than from only array-based sites. Likewise, the allowance of heterozygous calls per window can be reduced from three to two in PLINK analysis, indicating better tolerance to sequencing errors. In addition, ROH_100_ were less detectable in the BirThree dataset, and we assume this could be established from the fact that shorter ROHs may be united into longer ones. Such a superior performance in detecting ROHs may arise from using the data in which sites with higher Mendelian error rates were preliminarily removed based on pedigree information as a pre-processing quality control step, which may have reduced sequencing errors that deviate from Mendelian inheritance. When we examined the distribution of ROH by size (length intervals) in both datasets, we found that applying the BirThree dataset can actually mitigate the differences between array-based and all variant sites, particularly on longer ROHs, and thereby strengthen the effectiveness of the BirThree dataset. However, to make correct assumption, the differences in SNP marker density between the 3.5KJPNv2 and BirThree datasets need to be considered extensively.

To accurately isolate the effects of sequencing errors from those of SNP marker density, we conducted additional analyses for assessing SNP density effects after making methodological refinements, specifically designed to equalize SNP density between the datasets, thereby ensuring that any resultant differences in the ROH analysis could be attributed solely to sequencing errors, not to variations in SNP density. Our analysis showed that there was no significant difference in the number of ROH_1500_ per individual between 3.5KJPNv2 and its pruned dataset, suggesting that SNP marker density does not substantially affect the detection of longer ROHs. Interestingly, the results for BirThree consistently showed an increased number of ROH_1500_ compared to results for both unfiltered and pruned 3.5KJPNv2. This indicates that despite its lower SNP density, the BirThree dataset might have effects in reducing sequencing errors that cannot be effectively removed in 3.5KJPNv2 dataset. However, this was markedly different for shorter ROHs. We observed a significant reduction in the number of ROH_100_ in the pruned 3.5KJPNv2 dataset compared to its unfiltered version. This points to the possibility that the sparser marker coverage in the pruned dataset may miss some homozygous regions and lead to fewer identified ROH_100_ overall, whereas the higher SNP density in the unfiltered dataset may be more likely to detect heterozygous sites within stretches of homozygosity, thereby splitting or creating additional ROH_100_ segments. Comparatively, the number of ROH_100_ in the BirThree dataset was even lower than in the pruned 3.5KJPNv2 dataset, suggesting that factors beyond SNP density, possibly sequencing errors, are still influencing shorter ROH detection in these datasets. However, the differences in SNP density depending on the minimal ROH lengths across the datasets did not have a notable effect on the results in our additional analysis, that included overlapped samples from both datasets, reaffirming that the additional application of pedigree information played a larger role.

Our functional analysis of runs of homozygosity (ROH) showed statistically significant pathway enrichments that may indicate areas experiencing selective pressure. Using BCFtools, we identified the ROH islands harboring the gene-families such as OR4, USP17 and TAS2R, which revealed statistically notable pathway enrichment processes related to protein deubiquitination and sensory perception, especially regarding taste and smell. These functions likely reflect adaptations to environmental conditions. For instance, the ability to find food sources and detect bitter compounds, which are often linked to toxins, can provide critical survival advantages. Previous research indicates genetic variations in TAS2R genes across human populations [27, 28]. Considerable variation in the perception of odorants has also long been established among populations, with some of this variation being attributed to genetic changes in olfactory receptor (OR) genes [29, 30]. This diversity is likely the result of natural selection favoring alleles that enhance the detection of bitter tastes or specific odors, which may vary based on different ancestries. In contrast, the ROH islands detected by PLINK were weakly associated with different processes, such as fatty acid metabolism and inositol phosphate signaling pathways. It is plausible that certain homozygous stretches of the genome may have been favored and conserved as ROH islands in the Japanese population through natural selection in response to certain environmental stimuli. Moreover, further exploration of shorter ROH holds promise for better understanding the roles of ROH islands in investigating the relationships with several diseases/traits phenotypes while also providing insights into population-specific genetic architectures and evolutionary events.

In the past, SNP array technology was the standard method for ROH detection, but the constraints in handling dense SNP regions led to failures in detecting shorter ROH segments. In recent years, the advent of NGS platforms allowed us to access large proportions of the genome in detail, but WGS technologies can generate more significant error rates than array-based ones. Notably, the error rates vary in different populations, and the Japanese population in particular exhibits a high rate of regarding mistakenly called heterozygotes, which is about 13,000 per genome, or approximately 4.5 SNPs per 1 Mb [3]. To reduce these error rates, a fruitful way to explore potential functionality of ROH segments would be to use WGS data together with leveraging pedigree information in bio-bank level cohorts. There are, however, several restrictions in using such sequencing technologies in most research, and these data are yet to be utilized to their full potential in ROH studies. In the TMM Project, high coverage WGS (>30x) data is consistently collected for expanded cohorts of both related and unrelated Japanese individuals on a large scale, including tens of thousands of participants, which can allow for higher-resolution detection of ROHs. Henceforth, our future works will center on ROH homozygosity mapping in collaboration with global ROH research communities by taking advantage of such high-coverage WGS data. We concur with previous research discussion [19] in that understanding where ROH affects diseases and traits in the genome and connecting these insights with previously identified significant loci from genome-wide association studies is a promising direction for further research.

To facilitate comparison with array data in our study, we trimmed the WGS dataset by defining OmniExpressExome array-specific regions, which may underlie potential precision variations. Despite adopting the alternative trimming method in our WGS data, we observed no significant differences in F_ROH_ between our results and previous study in Japanese population [13], thereby supporting the robustness of our findings. As a limitation, our study did not investigate the possibility of uniparental isodisomy and hemizygous deletion, which could lead to extended homozygosity, and such types of potential cytogenetic abnormalities should be taken into consideration in further studies.

In conclusion, our study demonstrates that by including unrelated individuals and family pedigree information, high-coverage genome sequencing enables detection of shorter ROHs, which are undetectable by genotyping arrays. Furthermore, while longer ROHs may be prone to sequencing errors, the integration of pedigree information can mitigate these inaccuracies. We also conducted a comparative analysis of the fine-scale detection of ROH segments, particularly in hotspot regions like ROH islands, in which we compared the two most representative tools in ROH research (BCFtools and PLINK). Our findings indicated that WGS dataset that incorporated pedigree data can exhibit significantly greater functional pathway enrichment. Additionally, we suggest that future improvements to BCFtools should focus on integrating sequencing errors into the HMM training model process to further enhance the accuracy of the ROH analyses.

## Materials and methods

### Whole-genome sequence data

We used two high-coverage (~30x) WGS datasets in the study: 3.5KJPNv2, which consists of data from a total of 3552 individuals participating in multiple Japanese cohorts, including the Tohoku Medical Megabank (TMM) Project cohorts [20], and BirThree, which was generated from 192 families (62 trios/106 septets/24 octets) consisting of a total of 1120 participants in the TMM Birth and Three-Generation (BirThree) Cohort [21]. Both datasets included 208 overlapping individuals. The TMM BirThree Cohort has the advantage of providing precise information on allele inheritance based on pedigree information available in the multigenerational study design, which made it possible for initial detection and removal of potential genotype error sites in the dataset.

To investigate the effect of marker density on ROH detection, we also generated trimmed datasets, in which genotypes are available only for SNP-array sites, of the two WGS datasets. We compared the numbers and the lengths of detected ROHs in the trimmed datasets with those in the initial datasets including all variant sites. We defined SNP sites on the Infinium OmniExpressExome-8 BeadChip (Illumina, San Diego, CA, USA) as “SNP array-based sites”.

### RoH detection

Previous studies have predicted that long ROH segments are commonly found in the centromere regions of different species, which could possibly be related to selective sweeps and meiotic drive in these regions [5, 31, 32]. These studies indicated the absence of SNPs in centromeric regions, and so we excluded these regions from our analyses to avoid overestimating ROH. To achieve this, we divided each chromosome into short and long arms.

Initially, variants were filtered only to biallelic SNPs (-m2 -M2 -v snps), with allele frequencies between 0.05 and 0.95 (-q 0.05 -Q 0.95), and without missing alleles (-g ^miss) by using BCFtools. We primarily used Bcftools/RoH [11] to search for ROHs. RoH regions were detected for each chromosome of each sample, and per-region data (-O r option) was collected. Memory usage was set to 20GB (-b 20480 option). We used a fine-scale genetic map which was constructed from WGS data on 150 unrelated participants in the TMM BirThree Cohort (https://jmorp.megabank.tohoku.ac.jp/downloads/tommo-genetic_map-20210907) [33] and compared the results by switching it to the 1KGP genetic map [34]. We then set the parameter value of the -G option to 30 to account for GT errors.

Next, we applied PLINK 1.90 for comparison [7]. We then followed the command line parameters suggested in the previous study with slight modifications for the purpose of our study [12]. In part, for considering sequencing errors that may break a long ROH, 1 heterozygous call per detection window is generally allowed, but it was suggested to allow 3 heterozygous calls per window for low-coverage WGS data in order to provide equivalent results to SNP-array data [3]. Using this suggestion, we evaluated PLINK parameter by adjusting the allowance of heterozygous genotypes in the homozygous window. The detailed options and parameters used in the analyses are shown in the additional Information (S1 Text).

For each sample, the number and summed lengths of detected ROHs (NROH and SROH, respectively) were collected according to their minimal length class (100 Kb, 300 Kb, and 1.5 Mb). Detailed descriptive statistics for detected ROH segments, with different minimal lengths were shown in Supplementary Tables S1A–F.

To distinguish the effects of sequencing errors from those of SNP marker density, we added the additional methods by creating a modified version of the 3.5KJPNv2 dataset (referred to as the pruned 3.5KJPNv2 dataset), which involved 10 iterations where variant sites in 3.5KJPNv2 were randomly selected to match the total number of variant sites in BirThree that comprised of fewer variants, and ROH calling were done again by using BCFtools. For each iteration, we calculated the number of ROHs and then averaged these values to define the mean NROH of 3.5KJPNv2 SNP-pruning dataset. Subsequently, the results from pruned dataset were compared with those from both unfiltered 3.5KJPNv2 and BirThree datasets. Additionally, to assess the effects of SNP density using an alternative approach, we performed the analyses exclusively on the 208 individuals shared between both datasets.

We also identified ROH islands by focusing on the regions that contained on top 0.1% and 0.5% of the most frequently occurring loci with the ROH segments overlapped across the individuals by using BEDtools [35]. Subsequently, we annotated the genes that overlapped with ROH islands, based on the GENCODE v46lift37 version [36]. We then used g:Profiler [37], that integrates various databases, including Gene Ontology (GO), Kyoto Encyclopedia of Genes and Genomes (KEGG), and Reactome (REAC), enabling us to identify whether molecular functions and biological pathways are over-represented in our targeted gene lists.

Genomic based inbreeding coefficient (F_ROH_) was estimated by dividing the cumulative length of all ROH segments in an individual's genome by the total length of the autosomal genome [13]. We also conducted the stratification analysis, grouping ROH segments into the different bins of size (S4A–H).

Additionally, we identified common sites with Mendelian-inconsistent calls that had frequently occurred in the three-generational BirThree dataset by PLINK’s mendel option (--mendel) and removed those sites from the 3.5KJPN dataset. Then, we compared the results in the 3.5KJPNv2 dataset, both with and without the inclusion of Mendelian-inconsistent error sites.

The study was approved by the Institutional Review Board of the Tohoku Medical Megabank Organization (initially approved with the approval number 2013-4-103, and last updated with the approval number 2023-4-075). The study was conducted in accordance with the Declaration of Helsinki.

## Supplementary information

Additional information for parameter adjustments in ROH detection tools

Detailed descriptive statistics summary of NROH and SROH

Detailed summary statistics for Functional Enrichment Analysis within ROH islands

Genomic Distributions of ROHs per individual (on chromosome 6 in 3.5KJPNv2 dataset only)

Genomic Distributions of ROH islands on autosomes

Full figures for Functional Enrichment Analysis of Annotated Genes within ROH islands

Assessments on SNP-density effects

Stratification of ROHs based on different ROH length intervals

## Data Availability

The datasets were used in the study with the permission of Tohoku Medical Megabank Organization (ToMMo). Sequence data will be made available upon request following approval from the Ethical Committee and the Materials and Information Distribution Review Committee of the Tohoku Medical Megabank Project.

## References

[1] Cooke NP, Mattiangeli V, Cassidy LM, Okazaki K, Stokes CA, Onbe S, et al. Ancient genomics reveals tripartite origins of Japanese populations. Sci Adv. 2021;7:1–15. 10.1126/sciadv.abh2419 PMC8448447 34533991

[2] Kirin M, McQuillan R, Franklin CS, Campbell H, Mckeigue PM, Wilson JF. Genomic runs of homozygosity record population history and consanguinity. PLoS One. 2010;5:e13996. 21085596 10.1371/journal.pone.0013996 PMC2981575

[3] Ceballos FC, Hazelhurst S, Ramsay M. Assessing runs of Homozygosity: a comparison of SNP Array and whole genome sequence low coverage data. BMC Genomics. 2018;19:1–12. 29378520 10.1186/s12864-018-4489-0 PMC5789638

[4] Wright S. Coefficients of Inbreeding and Relationship Author (s): Sewall Wright Source: The American Naturalist, Vol. 56, No. 645 (Jul. - Aug., 1922), pp. 330-338 Published by: The University of Chicago Press for The American Society of Naturalists Sta. Am Nat. 1922;56:330–8.

[5] McQuillan R, Leutenegger AL, Abdel-Rahman R, Franklin CS, Pericic M, Barac-Lauc L, et al. Runs of homozygosity in European populations. Am J Hum Genet. 2008;83:359–72. 18760389 10.1016/j.ajhg.2008.08.007 PMC2556426

[6] Broman KW, Weber JL. Long homozygous chromosomal segments in reference families from the Centre d’Etude du Polymorphisme Humain. Am J Hum Genet. 1999;65:1493–500. 10577902 10.1086/302661 PMC1288359

[7] Chang CC, Chow CC, Tellier LCAM, Vattikuti S, Purcell SM, Lee JJ. Second-generation PLINK: Rising to the challenge of larger and richer datasets. Gigascience. 2015;4:7. 25722852 10.1186/s13742-015-0047-8 PMC4342193

[8] Silva GAA, Harder AM, Kirksey KB, Mathur S, Willoughby JR. Detectability of runs of homozygosity is influenced by analysis parameters and population-specific demographic history. PLoS Comput Biol. 2024;20:e1012566. 39480880 10.1371/journal.pcbi.1012566 PMC11556709

[9] Browning SR, Browning BL. High-resolution detection of identity by descent in unrelated individuals. Am J Hum Genet. 2010;86:526–39. 20303063 10.1016/j.ajhg.2010.02.021 PMC2850444

[10] Magi A, Tattini L, Palombo F, Benelli M, Gialluisi A, Giusti B, et al. H3M2: detection of runs of homozygosity from whole-exome sequencing data. Bioinformatics. 2014;30:2852–9. 24966365 10.1093/bioinformatics/btu401

[11] Narasimhan V, Danecek P, Scally A, Xue Y, Tyler-Smith C, Durbin R. BCFtools/RoH: A hidden Markov model approach for detecting autozygosity from next-generation sequencing data. Bioinformatics. 2016;32:1749–51. 26826718 10.1093/bioinformatics/btw044 PMC4892413

[12] Joshi PK, Esko T, Mattsson H, Eklund N, Gandin I, Nutile T, et al. Directional dominance on stature and cognition in diverse human populations. Nature. 2015;523:459–62. 26131930 10.1038/nature14618 PMC4516141

[13] Clark DW, Okada Y, Moore KHS, Mason D, Pirastu N, Gandin I, et al. Associations of autozygosity with a broad range of human phenotypes. Nat Commun. 2019;10:1–17. 31673082 10.1038/s41467-019-12283-6 PMC6823371

[14] Johnson EC, Evans LM, Keller MC. Relationships between estimated autozygosity and complex traits in the UK Biobank. PLoS Genet. 2018;14:1–19. 10.1371/journal.pgen.1007556 PMC6082573 30052639

[15] Hemstrom W, Grummer JA, Luikart G, Christie MR. Next-generation data filtering in the genomics era. Nat Rev Genet. 2024;25:750–67. 10.1038/s41576-024-00738-6 38877133

[16] Goodwin S, McPherson JD, McCombie WR. Coming of age: ten years of next-generation sequencing technologies. Nat Rev Genet. 2016;17:333–51. 27184599 10.1038/nrg.2016.49 PMC10373632

[17] Stoler N, Nekrutenko A. Sequencing error profiles of Illumina sequencing instruments. NAR Genom Bioinform. 2021;3:1–9. 10.1093/nargab/lqab019 PMC8002175 33817639

[18] Ma X, Shao Y, Tian L, Flasch DA, Mulder HL, Edmonson MN, et al. Analysis of error profiles in deep next-generation sequencing data. Genome Biol. 2019;20:1–15. 30867008 10.1186/s13059-019-1659-6 PMC6417284

[19] Ceballos FC, Joshi PK, Clark DW, Ramsay M, Wilson JF. Runs of homozygosity: Windows into population history and trait architecture. Nat Rev Genet. 2018;19:220–34. 29335644 10.1038/nrg.2017.109

[20] Tadaka S, Katsuoka F, Ueki M, Kojima K, Makino S, Saito S, et al. 3.5KJPNv2: an allele frequency panel of 3552 Japanese individuals including the X chromosome. Hum Genome Var. 2019;6:1–9. 31240104 10.1038/s41439-019-0059-5 PMC6581902

[21] Kuriyama S, Metoki H, Kikuya M, Obara T, Ishikuro M, Yamanaka C, et al. Cohort Profile: Tohoku Medical Megabank Project Birth and Three-Generation Cohort Study (TMM BirThree Cohort Study): Rationale, progress and perspective. Int J Epidemiol. 2020;49:18–19M. 31504573 10.1093/ije/dyz169 PMC7124511

[22] Kuriyama S, Yaegashi N, Nagami F, Arai T, Kawaguchi Y, Osumi N, et al. The Tohoku Medical Megabank Project: Design and mission. J Epidemiol. 2016;26:493–511. 27374138 10.2188/jea.JE20150268 PMC5008970

[23] Yasuda J, Kinoshita K, Katsuoka F, Danjoh I, Sakurai-Yageta M, Motoike IN, et al. Genome analyses for the Tohoku Medical Megabank Project towards establishment of personalized healthcare. J Biochem. 2019;165:139–58. 30452759 10.1093/jb/mvy096

[24] Frazer KA, Ballinger DG, Cox DR, Hinds DA, Stuve LL, Gibbs RA, et al. A second generation human haplotype map of over 3.1 million SNPs. Nature. 2007;449:851–61. 17943122 10.1038/nature06258 PMC2689609

[25] Ku CS, Naidoo N, Teo SM, Pawitan Y. Regions of homozygosity and their impact on complex diseases and traits. Hum Genet. 2011;129:1–15. 21104274 10.1007/s00439-010-0920-6

[26] Pilipenko VV, He H, Kurowski BG, Alexander ES, Zhang X, Ding L, et al. Using Mendelian inheritance errors as quality control criteria in whole genome sequencing data set. BMC Proc. 2014;8:1–5. 10.1186/1753-6561-8-S1-S21 PMC4144465 25519373

[27] Kim U, Wooding S, Ricci D, Jorde LB, Drayna D. Worldwide haplotype diversity and coding sequence variation at human bitter taste receptor loci. Hum Mutat. 2005;26:199–204. 16086309 10.1002/humu.20203

[28] Wooding SP, Ramirez VA. Global population genetics and diversity in the TAS2R bitter taste receptor family. Front Genet. 2022;13:1–13. 10.3389/fgene.2022.952299 PMC9592824 36303543

[29] Ayabe-Kanamura S, Schicker I, Laska M, Hudson R, Distel H, Kobayakawa T, et al. Differences in perception of everyday odors: a Japanese-German cross-cultural study. Chem Senses. 1998;23:31–38. 9530967 10.1093/chemse/23.1.31

[30] Keller A, Zhuang H, Chi Q, Vosshall LB, Matsunami H. Genetic variation in a human odorant receptor alters odour perception. Nature. 2007;449:468–72. 17873857 10.1038/nature06162

[31] Lencz T, Lambert C, DeRosse P, Burdick KE, Morgan TV, Kane JM, et al. Runs of homozygosity reveal highly penetrant recessive loci in schizophrenia. Proc Natl Acad Sci USA. 2007;104:19942–7. 18077426 10.1073/pnas.0710021104 PMC2148402

[32] Williamson SH, Hubisz MJ, Clark AG, Payseur BA, Bustamante CD, Nielsen R. Localizing recent adaptive evolution in the human genome. PLoS Genet. 2007;3:0901–15. 10.1371/journal.pgen.0030090 PMC1885279 17542651

[33] Takayama J, Makino S, Funayama T, Ueki M, Narita A, Murakami K et al. A fine-scale genetic map of the Japanese population. Clin Genet. 2024;106:284–92. 10.1111/cge.14536 38719617

[34] Altshuler DL, Durbin RM, Abecasis GR, Bentley DR, Chakravarti A, Clark AG, et al. A map of human genome variation from population-scale sequencing. Nature. 2010;467:1061–73. 20981092 10.1038/nature09534 PMC3042601

[35] Quinlan AR, Hall IM. BEDTools: A flexible suite of utilities for comparing genomic features. Bioinformatics. 2010;26:841–2. 20110278 10.1093/bioinformatics/btq033 PMC2832824

[36] Harrow J, Frankish A, Gonzalez JM, Tapanari E, Diekhans M, Kokocinski F, et al. GENCODE: the reference human genome annotation for the ENCODE project. Genome Res. 2012;22:1760–74. 22955987 10.1101/gr.135350.111 PMC3431492

[37] Kolberg L, Raudvere U, Kuzmin I, Adler P, Vilo J, Peterson H. G:Profiler-interoperable web service for functional enrichment analysis and gene identifier mapping (2023 update). Nucleic Acids Res. 2023;51:W207–W212. 37144459 10.1093/nar/gkad347 PMC10320099

