# Supplementary material for: Profiling of runs of homozygosity from whole-genome sequence data in Japanese biobank
Source: J Hum Genet. 2025 Apr 3;70(6):287–96. doi: 10.1038/s10038-025-01331-3 (PMC12058513; doi:10.1038/s10038-025-01331-3)
Supplement: Supplementary file 1 — Additional information for parameter adjustments in ROH detection tools [file 10038_2025_1331_MOESM1_ESM.pdf]

S1 Text. Supplementary Materials

Parameter adjustment details:

In this study, we examined the presence of runs of homozygosity (ROH) in Japanese individuals by using two high-coverage WGS datasets. We used BCFtools/RoH and PLINK 1.90 in which the following parameters were applied to adjust the detectability of ROH according to the different compositions in each cohort.

BCFtools/RoH:

<Extraction of data>

```
bcftools view -m2 -M2 -v snps \
              -g ^miss \
              -q 0.05 -Q 0.95 \
              -O b -o ${BCFFILE} ${THREADS_OPT} ${VCFFILE}
```

<Detection of ROH>

```
bcftools roh  -G30 \
              -b 20480 \
              -m ${MAPFILE} \
              -R ${REGION} \
              -O r -o ${OUTFILE} ${BCFFILE}
```

PLINK:

```
plink   --memory 20480 \
        --bcf ${BCFFILE} \
```

```
--allow-extra-chr \
--keep-allele-order \
--geno 0.03 \
--homozyg-snp 50 \
--homozyg-kb 100 \
--homozyg-density 50 \
--homozyg-gap 1000 \
--homozyg-window-snp 50 \
--homozyg-window-het (1 or 2 or 3 or 4) \
--homozyg-window-missing 5 \
--out ${OUTFILE}
```
