# Supplementary material for: Profiling of runs of homozygosity from whole-genome sequence data in Japanese biobank
Source: J Hum Genet. 2025 Apr 3;70(6):287–96. doi: 10.1038/s10038-025-01331-3 (PMC12058513; doi:10.1038/s10038-025-01331-3)
Supplement: Supplementary file 2 — Detailed descriptive statistics summary of NROH and SROH [file 10038_2025_1331_MOESM2_ESM.pdf]

Table S1A. Basic statistics for the total numbers of ROH (NROH) detected in 3.5KJPNv2 dataset and BirThree dataset with the implementation of a ROH length threshold of 100 Kb.

3.5KJPNv2

	Min.	Median	Mean	Max.
BCFtools/RoH (all variant sites/ ToMMo genetic map)	1,908	2,193	2,190	2,353
BCFtools/RoH (only SNP array-based sites/ ToMMo genetic map)	151	193	194	259
PLINK (all variant sites/--homozyg-window-het 1)	2,494	2,740	2,739	2,940
PLINK (all variant sites/--homozyg-window-het 2)	2,754	3,086	3,083	3,242
PLINK (all variant sites/--homozyg-window-het 3)	2,899	3,308	3,302	3,477
PLINK (all variant sites/--homozyg-window-het 4)	3,036	3,476	3,471	3,636
PLINK (only SNP array-based sites/--homozyg-window-het 1)	582	696	697	780
BCFtools/RoH (all variant sites/1KGP genetic map)	1,911	2,195	2,192	2,361
BCFtools/RoH (only SNP array-based sites/1KGP genetic map)	150	195	196	268
**Removed loci with Mendel error rate > 0.05 predicted in BirThree**				
BCFtools/RoH (all variant sites / ToMMo genetic map)	1,900	2,195	2,191	2,352
BCFtools/RoH (only SNP array-based sites/ ToMMo genetic map)	–	–	–	–
PLINK (all variant sites/--homozyg-window-het 1)	2,482	2,740	2,739	2,944
PLINK (all variant sites/--homozyg-window-het 2)	2,737	3,085	3,082	3,243
PLINK (all variant sites/--homozyg-window-het 3)	–	–	–	–
PLINK (all variant sites/--homozyg-window-het 4)	–	–	–	–
PLINK (only SNP array-based sites/--homozyg-window-het 1)	–	–	–	–

**Removed loci with Mendel error rate > 0.01 predicted in BirThree**				
BCFtools/RoH (all variant sites/ ToMMo genetic map)	1,894	2,198	2,194	2,355
BCFtools/RoH (only SNP array-based sites/ ToMMo genetic map)	–	–	–	–
PLINK (all variant sites/--homozyg-window-het 1)	2,460	2,743	2,742	2,949
PLINK (all variant sites/--homozyg-window-het 2)	2,718	3,085	3,081	3,242
PLINK (all variant sites/--homozyg-window-het 3)	–	–	–	–
PLINK (all variant sites/--homozyg-window-het 4)	–	–	–	–
PLINK (only SNP array-based sites/--homozyg-window-het 1)	–	–	–	–

BirThree

	**Min.**	**Median**	**Mean**	**Max.**
BCFtools/RoH (all variant sites/ ToMMo genetic map)	1,481	1,716	1,713	1,813
BCFtools/RoH (only SNP array-based sites/ ToMMo genetic map)	38	67	67	95
PLINK (all variant sites/--homozyg-window-het 1)	2,446	2,730	2,726	2,872
PLINK (all variant sites/--homozyg-window-het 2)	2,732	3,075	3,072	3,201
PLINK (all variant sites/--homozyg-window-het 3)	2,963	3,328	3,324	3,450
PLINK (all variant sites/--homozyg-window-het 4)	3,173	3,535	3,532	3,676
PLINK (only SNP array-based sites/--homozyg-window-het 1)	274	358	357	405

*PLINK "--homozyg-window-het" option values were set to a range of 1 to 4, i.e., allowing from one to four heterozygous calls per window.

These are abbreviated as "Het_1", "Het_2" , "Het_3", and "Het_4", respectively. Although the SNP array data is unavailable, we have effectively trimmed whole genome sequencing (WGS) data to restrict our analysis exclusively on the regions specified by OmniExpressExome Array.

Total loci in genome-wide regions of 3.5KJPNv2 dataset: 5,198,319 loci

Total loci in array-specific regions of 3.5KJPNv2 dataset: 655,966 loci

Total loci in genome-wide regions of BirThree dataset: 3,796,555 loci

Total loci in array-specific regions of BirThree dataset: 385,176 loci

The units in the total sums of ROH (SROH) are expressed in terms of base pairs (bp).

1KGP means 1000 Genomes Project.

Table S1B. Basic statistics for the total sums of ROH (SROH) detected in 3.5KJPNv2 dataset and BirThree dataset with the implementation of a ROH length threshold of 100 Kb.

3.5KJPNv2

	Min.	Median	Mean	Max.
BCFtools/RoH (all variant sites/ ToMMo genetic map)	534,370,587	576,096,237	582,285,742	895,705,740
BCFtools/RoH (only SNP array-based sites/ ToMMo genetic map)	85,695,949	118,755,673	127,461,992	521,425,163
PLINK (all variant sites/--homozyg-window-het 1)	540,240,433	588,622,293	594,581,242	910,097,234
PLINK (all variant sites/--homozyg-window-het 2)	674,329,533	715,383,436	720,861,960	1,017,566,599
PLINK (all variant sites/--homozyg-window-het 3)	756,187,433	795,102,003	800,370,778	1,086,507,890
PLINK (all variant sites/--homozyg-window-het 4)	812,674,520	853,904,322	858,847,627	1,138,886,646
PLINK (only SNP array-based sites/--homozyg-window-het 1)	264,534,405	310,819,343	318,169,943	680,967,148
BCFtools/RoH (all variant sites/1KGP genetic map)	534,216,750	576,214,255	582,277,572	895,546,537
BCFtools/RoH (only SNP array-based sites/1KGP genetic map)	87,781,777	119,931,597	128,472,300	521,902,697
**Removed loci with Mendel error rate > 0.05 predicted in BirThree**				
BCFtools/RoH (all variant sites/ ToMMo genetic map)	535,552,194	578,027,758	584,098,911	896,780,734
BCFtools/RoH (only SNP array-based sites/ ToMMo genetic map)	–	–	–	–
PLINK (all variant sites/--homozyg-window-het 1)	541,402,934	590,508,322	596,466,091	911,519,017
PLINK (all variant sites/--homozyg-window-het 2)	676,035,603	717,465,209	722,893,953	1,018,881,236

PLINK (all variant sites/--homozyg-window-het 3)	–	–	–	–
PLINK (all variant sites/--homozyg-window-het 4)	–	–	–	–
PLINK (only SNP array-based sites/--homozyg-window-het 1)	–	–	–	–

**Removed loci with Mendel error rate > 0.01predicted in BirThree**				
BCFtools/RoH (all variant sites/ ToMMo genetic map)	539,117,711	580,997,895	586,972,503	900,996,997
BCFtools/RoH (only SNP array-based sites/ ToMMo genetic map)	–	–	–	–
PLINK (all variant sites/--homozyg-window-het 1)	544,142,882	594,126,586	600,145,006	915,137,655
PLINK (all variant sites/--homozyg-window-het 2)	679,330,756	720,513,509	725,948,749	1,021,315,657
PLINK (all variant sites/--homozyg-window-het 3)	–	–	–	–
PLINK (all variant sites/--homozyg-window-het 4)	–	–	–	–
PLINK (only SNP array-based sites/--homozyg-window-het 1)	–	–	–	–

BirThree

	**Min.**	**Median**	**Mean**	**Max.**
BCFtools/RoH (all variant sites/ ToMMo genetic map)	436,013,285	501,623,732	505,931,167	783,901,154
BCFtools/RoH (only SNP array-based sites/ ToMMo genetic map)	34,669,360	55,373,796	61,110,307	398,243,796
PLINK (all variant sites/--homozyg-window-het 1)	500,974,238	624,000,560	627,622,136	894,224,738
PLINK (all variant sites/--homozyg-window-het 2)	673,575,486	755,369,740	758,579,183	1,008,071,538
PLINK (all variant sites/--homozyg-window-het 3)	791,755,395	845,424,042	848,691,286	1,086,389,235
PLINK (all variant sites/--homozyg-window-het 4)	867,207,971	915,815,702	919,263,432	1,150,862,539
PLINK (only SNP array-based sites/--homozyg-window-het 1)	151,509,589	201,740,606	206,508,058	524,814,573

Table S1C. Basic statistics for the total numbers of ROH (NROH) detected in 3.5KJPNv2 dataset and BirThree dataset with the implementation of a ROH length threshold of 1.5 Mb.

3.5KJPNv2

	Min.	Median	Mean	Max.
BCFtools/RoH (all variant sites/ ToMMo genetic map)	0.00	7.00	8.37	60.00
BCFtools/RoH (only SNP array-based sites/ ToMMo genetic map)	1.00	10.00	10.16	46.00
PLINK (all variant sites/--homozyg-window-het 1)	0.00	3.00	4.68	95.00
PLINK (all variant sites/--homozyg-window-het 2)	0.00	5.00	6.59	81.00
PLINK (all variant sites/--homozyg-window-het 3)	0.00	7.00	8.53	71.00
PLINK (all variant sites/--homozyg-window-het 4)	0.00	9.00	10.06	64.00
PLINK (only SNP array-based sites/--homozyg-window-het 1)	1.00	10.00	10.94	48.00
BCFtools/RoH (all variant sites/1KGP genetic map)	0.00	7.00	8.28	62.00
BCFtools/RoH (only SNP array-based sites/1KGP genetic map)	1.00	10.00	10.18	46.00
**Removed loci with Mendel error rate > 0.05 predicted in BirThree**				
BCFtools/RoH (all variant sites/ ToMMo genetic map)	1.00	7.00	8.49	61.00
BCFtools/RoH (only SNP array-based sites/ ToMMo genetic map)	–	–	–	–
PLINK (all variant sites/--homozyg-window-het 1)	0.00	3.00	4.76	93.00
PLINK (all variant sites/--homozyg-window-het 2)	0.00	5.00	6.70	77.00
PLINK (all variant sites/--homozyg-window-het 3)	–	–	–	–
PLINK (all variant sites/--homozyg-window-het 4)	–	–	–	–
PLINK (only SNP array-based sites/--homozyg-window-het 1)	–	–	–	–

**Removed loci with Mendel error rate > 0.01 predicted in BirThree**				
BCFtools/RoH (all variant sites/ ToMMo genetic map)	1.00	8.00	8.74	60.00
BCFtools/RoH (only SNP array-based sites/ ToMMo genetic map)	—	—	—	—
PLINK (all variant sites/--homozyg-window-het 1)	0.00	3.00	4.86	93.00
PLINK (all variant sites/--homozyg-window-het 2)	0.00	5.00	6.90	78.00
PLINK (all variant sites/--homozyg-window-het 3)	—	—	—	—
PLINK (all variant sites/--homozyg-window-het 4)	—	—	—	—
PLINK (only SNP array-based sites/--homozyg-window-het 1)	—	—	—	—

BirThree

	Min.	Median	Mean	Max.
BCFtools/RoH (all variant sites/ ToMMo genetic map)	2.00	10.00	10.44	41.00
BCFtools/RoH (only SNP array-based sites/ ToMMo genetic map)	0.00	7.00	7.71	38.00
PLINK (all variant sites/--homozyg-window-het 1)	0.00	7.00	7.18	37.00
PLINK (all variant sites/--homozyg-window-het 2)	2.00	10.00	10.88	39.00
PLINK (all variant sites/--homozyg-window-het 3)	5.00	14.00	14.16	42.00
PLINK (all variant sites/--homozyg-window-het 4)	8.00	16.00	16.82	45.00
PLINK (only SNP array-based sites/--homozyg-window-het 1)	3.00	10.00	11.06	38.00

Table S1D. Basic statistics for the total sums of ROH (SROH) detected in 3.5KJPNv2 dataset and BirThree dataset with the implementation of a ROH length threshold of 1.5 Mb.

3.5KJPNv2

	Min.	Median	Mean	Max.
BCFtools/RoH (all variant sites/ ToMMo genetic map)	0	14,788,891	23,464,275	408,841,409
BCFtools/RoH (only SNP array-based sites/ ToMMo genetic map)	2,225,703	20,536,030	29,879,304	442,998,794
PLINK (all variant sites/--homozyg-window-het 1)	0	5,120,842	12,701,591	346,706,317
PLINK (all variant sites/--homozyg-window-het 2)	0	9,667,408	17,901,917	377,645,167
PLINK (all variant sites/--homozyg-window-het 3)	0	14,077,094	22,679,954	398,495,364
PLINK (all variant sites/--homozyg-window-het 4)	0	17,676,115	26,260,213	408,356,599
PLINK (only SNP array-based sites/--homozyg-window-het 1)	2,533,675	21,143,884	30,192,850	433,885,303
BCFtools/RoH (all variant sites/1KGP genetic map)	0	14,545,749	23,242,534	409,979,717
BCFtools/RoH (only SNP array-based sites/1KGP genetic map)	2,634,918	20,619,632	29,906,618	443,297,245
**Removed loci with Mendel error rate > 0.05 predicted in BirThree**				
BCFtools/RoH (all variant sites/ ToMMo genetic map)	1,506,531	15,177,451	23,975,600	409,826,292
BCFtools/RoH (only SNP array-based sites/ ToMMo genetic map)	–	–	–	–
PLINK (all variant sites/--homozyg-window-het 1)	0	5,366,037	13,146,947	354,175,310
PLINK (all variant sites/--homozyg-window-het 2)	0	10,057,478	18,446,382	383,751,399
PLINK (all variant sites/--homozyg-window-het 3)	–	–	–	–

PLINK (all variant sites/--homozyg-window-het 4)	–	–	–	–
PLINK (only SNP array-based sites/--homozyg-window-het 1)	–	–	–	–

Removed loci with Mendel error rate > 0.01predicted in BirThree

BCFtools/RoH (all variant sites/ ToMMo genetic map)	1,531,628	15,735,338	24,655,266	413,162,318
BCFtools/RoH (only SNP array-based sites/ ToMMo genetic map)	–	–	–	–
PLINK (all variant sites/--homozyg-window-het 1)	0	5,698,793	13,814,297	365,997,815
PLINK (all variant sites/--homozyg-window-het 2)	0	10,690,627	19,230,492	388,733,065
PLINK (all variant sites/--homozyg-window-het 3)	–	–	–	–
PLINK (all variant sites/--homozyg-window-het 4)	–	–	–	–
PLINK (only SNP array-based sites/--homozyg-window-het 1)	–	–	–	–

BirThree

	**Min.**	**Median**	**Mean**	**Max.**
BCFtools/RoH (all variant sites/ ToMMo genetic map)	3,133,771	21,354,319	27,486,031	371,735,386
BCFtools/RoH (only SNP array-based sites/ ToMMo genetic map)	0	16,043,873	22,104,265	371,174,200
PLINK (all variant sites/--homozyg-window-het 1)	0	14,450,298	20,846,395	360,969,243
PLINK (all variant sites/--homozyg-window-het 2)	3,375,284	22,818,472	29,116,852	374,591,074
PLINK (all variant sites/--homozyg-window-het 3)	11,106,882	30,341,399	36,505,896	382,886,382
PLINK (all variant sites/--homozyg-window-het 4)	16,590,371	36,763,535	42,598,045	384,084,851
PLINK (only SNP array-based sites/--homozyg-window-het 1)	5,092,298	21,401,696	27,499,818	365,268,859

We predicted 102,159 loci and 311,898 loci with Mendelian error rate exceeding 5% or 1% in BirThree dataset, out of which 20,403 and 57,037 sites were found in 3.5KJPNv2 dataset, respectively.

Table S1E. Basic statistics for the total numbers of ROH (NROH) detected in 3.5KJPNv2 dataset and BirThree dataset with the implementation of a ROH length threshold of 300 Kb.

3.5KJPNv2

	Min.	Median	Mean	Max.
BCFtools/RoH (all variant sites/ ToMMo genetic map)	499	567	568	646
BCFtools/RoH (only SNP array-based sites/ ToMMo genetic map)	108	148	148	193
PLINK (all variant sites/--homozyg-window-het 1)	387	454	455	585
PLINK (all variant sites/--homozyg-window-het 2)	546	617	617	698
PLINK (all variant sites/--homozyg-window-het 3)	621	712	712	786
PLINK (all variant sites/--homozyg-window-het 4)	678	779	779	866
PLINK (only SNP array-based sites/--homozyg-window-het 1)	357	429	430	496

BirThree

	Min.	Median	Mean	Max.
BCFtools/RoH (all variant sites/ ToMMo genetic map)	467	535	534	592
BCFtools/RoH (only SNP array-based sites/ ToMMo genetic map)	36	59	59	87
PLINK (all variant sites/--homozyg-window-het 1)	356	512	512	571
PLINK (all variant sites/--homozyg-window-het 2)	556	677	677	738
PLINK (all variant sites/--homozyg-window-het 3)	675	780	780	848
PLINK (all variant sites/--homozyg-window-het 4)	744	858	858	930
PLINK (only SNP array-based sites/--homozyg-window-het 1)	207	277	277	326

Table S1F. Basic statistics for the total sums of ROH (SROH) detected in 3.5KJPNv2 dataset and BirThree dataset with the implementation of a ROH length threshold of 300 Kb.

3.5KJPNv2

	Min.	Median	Mean	Max.
BCFtools/RoH (all variant sites/ ToMMo genetic map)	253,493,209	294,957,369	302,075,997	655,493,346
BCFtools/RoH (only SNP array-based sites/ ToMMo genetic map)	75,921,293	109,003,805	117,652,562	511,842,935
PLINK (all variant sites/--homozyg-window-het 1)	173,979,345	213,757,658	220,984,472	582,664,251
PLINK (all variant sites/--homozyg-window-het 2)	264,309,361	303,938,005	311,078,757	662,730,215
PLINK (all variant sites/--homozyg-window-het 3)	320,118,560	359,528,218	366,571,657	709,342,442
PLINK (all variant sites/--homozyg-window-het 4)	356,895,750	398,939,189	405,916,932	747,867,508
PLINK (only SNP array-based sites/--homozyg-window-het 1)	212,479,255	253,830,968	261,267,926	633,548,965

BirThree

	Min.	Median	Mean	Max.
BCFtools/RoH (all variant sites/ ToMMo genetic map)	255,453,172	291,747,918	296,065,657	591,377,238
BCFtools/RoH (only SNP array-based sites/ ToMMo genetic map)	33,186,649	53,621,170	59,270,784	397,257,438
PLINK (all variant sites/--homozyg-window-het 1)	160,077,389	257,339,622	261,889,649	564,569,829
PLINK (all variant sites/--homozyg-window-het 2)	273,372,738	353,311,530	357,352,402	651,777,621
PLINK (all variant sites/--homozyg-window-het 3)	364,321,114	416,044,830	420,050,354	699,589,249
PLINK (all variant sites/--homozyg-window-het 4)	404,339,691	463,040,936	467,005,817	740,107,162

PLINK (only SNP array-based sites/--homozyg-window-het 1)	135,878,973	183,235,902	188,337,153	505,219,445

Table S1G. Basic statistics for the genomic inbreeding coefficient (F_{ROH}) detected in 3.5KJPNv2 dataset with the implementation of a ROH length threshold of 1.5 MB.

3.5KJPNv2

	**Mean F_{ROH}**	**Standard Deviations**
BCFtools/RoH (all variant sites/ ToMMo genetic map)	0.007821	0.012484
BCFtools/RoH (only SNP array-based sites/ ToMMo genetic map)	0.00996	0.013333
PLINK (all variant sites/--homozyg-window-het 1)	0.004234	0.01038
PLINK (all variant sites/--homozyg-window-het 2)	0.005967	0.011538
PLINK (all variant sites/--homozyg-window-het 3)	0.00756	0.012135
PLINK (all variant sites/--homozyg-window-het 4)	0.008753	0.012401
PLINK (only SNP array-based sites/--homozyg-window-het 1)	0.010064	0.013185
