# Supplementary material for: Profiling of runs of homozygosity from whole-genome sequence data in Japanese biobank
Source: J Hum Genet. 2025 Apr 3;70(6):287–96. doi: 10.1038/s10038-025-01331-3 (PMC12058513; doi:10.1038/s10038-025-01331-3)
Supplement: Supplementary file 3 — Detailed summary statistics for Functional Enrichment Analysis within ROH islands [file 10038_2025_1331_MOESM3_ESM.pdf]

Table S2A. Detailed summary statistics of Functional Enrichment Analysis within Runs of Homozygosity (ROH) Islands. This table presents the detailed statistics of functional enrichment analysis on genes identified within runs of homozygosity (ROH) islands (> 100 KB), detected in the BirThree dataset via BCFtools (by setting 99.9th percentile threshold based on the frequencies of overlapping ROH_{100} regions shared among individuals). The gProfiler tool was utilized to identify enriched biological pathways (BP), molecular functions (MF), and cellular components (CC) from Gene Ontology (GO), KEGG, and Reactome. The intersections column displays the genes from the query list that overlap with each functional term, contributing to its statistical significance. Detailed information regarding statistics can be found on gProfiler website.

source	term_name	term_id	highlighted	adjusted_p_value	negative_log10_of_adjusted_p_value	term_size	query_size	intersection_size	effective_domain_size	intersections
GO:MF	cysteine-type deubiquitinase activity	GO:0004843	TRUE	1.05E-26	25.97906294	117	68	20	20212	USP17L10,USP17L11,USP17L12,USP17L13,USP17L15,USP17L17,USP17L18,USP17L19,USP17L20,USP17L21,USP17L22,USP17L23,USP17L24,USP17L26,USP17L27,USP17L28,USP17L29,USP17L30,USP17L5,USP38
GO:MF	deubiquitinase activity	GO:0101005	FALSE	3.08E-26	25.51195338	123	68	20	20212	USP17L10,USP17L11,USP17L12,USP17L13,USP17L15,USP17L17,USP17L18,USP17L19,USP17L20,USP17L21,USP17L22,USP17L23,USP17L24,USP17L26,USP17L27,USP17L28,USP17L29,USP17L30,USP17L5,USP38
GO:MF	ubiquitin-like protein peptidase activity	GO:0019783	FALSE	1.92E-25	24.71745951	134	68	20	20212	USP17L10,USP17L11,USP17L12,USP17L13,USP17L15,USP17L17,USP17L18,USP17L19,USP17L20,USP17L21,USP17L22,USP17L23,USP17L24,USP17L26,USP17L27,USP17L28,USP17L29,USP17L30,USP17L5,USP38
GO:MF	cysteine-type peptidase activity	GO:0008234	FALSE	6.72E-22	21.1727389	198	68	20	20212	USP17L10,USP17L11,USP17L12,USP17L13,USP17L15,USP17L17,USP17L18,USP17L19,USP17L20,USP17L21,USP17L22,USP17L23,USP17L24,USP17L26,USP17L27,USP17L28,USP17L29,USP17L30,USP17L5,USP38

GO:MF	peptidase activity	GO:0008233	FALSE	4.81E-12	11.31803605	625	68	20	20212	USP17L10,USP17L11,USP17L12,USP17L13,USP17L15,USP17L17,USP17L18,USP17L19,USP17L20,USP17L21,USP17L22,USP17L23,USP17L24,USP17L26,USP17L27,USP17L28,USP17L29,USP17L30,USP17L5,USP38
GO:MF	bitter taste receptor activity	GO:0033038	TRUE	1.32E-10	9.880732468	20	68	7	20212	TAS2R14,TAS2R20,TAS2R30,TAS2R31,TAS2R43,TAS2R46,TAS2R50
GO:MF	taste receptor activity	GO:0008527	FALSE	1.10E-09	8.958945242	26	68	7	20212	TAS2R14,TAS2R20,TAS2R30,TAS2R31,TAS2R43,TAS2R46,TAS2R50
GO:MF	hyaluronic acid binding	GO:0005540	TRUE	3.19E-07	6.495946904	30	68	6	20212	USP17L24,USP17L26,USP17L27,USP17L28,USP17L29,USP17L30
GO:MF	hydrolase activity	GO:0016787	FALSE	0.000391363	3.407419945	2478	68	24	20212	ATAD5,INPP4B,LARS1,PSMC4,USP17L10,USP17L11,USP17L12,USP17L13,USP17L15,USP17L17,USP17L18,USP17L19,USP17L20,USP17L21,USP17L22,USP17L23,USP17L24,USP17L26,USP17L27,USP17L28,USP17L29,USP17L30,USP17L5,USP38
GO:MF	catalytic activity, acting on a protein	GO:0140096	FALSE	0.002472606	2.606845145	2355	68	22	20212	RANBP2,SH3RF2,USP17L10,USP17L11,USP17L12,USP17L13,USP17L15,USP17L17,USP17L18,USP17L19,USP17L20,USP17L21,USP17L22,USP17L23,USP17L24,USP17L26,USP17L27,USP17L28,USP17L29,USP17L30,USP17L5,USP38
GO:BP	protein deubiquitination	GO:0016579	TRUE	4.90E-26	25.31021184	125	67	20	21031	USP17L10,USP17L11,USP17L12,USP17L13,USP17L15,USP17L17,USP17L18,USP17L19,USP17L20,USP17L21,USP17L22,USP17L23,USP17L24,USP17L26,USP17L27,USP17L28,USP17L29,USP17L30,USP17L5,USP38
GO:BP	protein modification by small protein removal	GO:0070646	FALSE	6.37E-25	24.19574	141	67	20	21031	USP17L10,USP17L11,USP17L12,USP17L13,USP17L15,USP17L17,USP17L18,USP17L19,USP17L20,USP17L21,USP17L22,USP17L23,USP17L24,USP17L26,USP17L27,USP17L28,USP17L29,USP17L30,USP17L5,USP38

GO:BP	protein modification by small protein conjugation or removal	GO:0070647	FALSE	1.90E-11	10.72071432	970	67	23	21031	RANBP2,SH3RF2,SPSB4,USP17L10,USP17L11,USP17L12,USP17L13,USP17L15,USP17L17,USP17L18,USP17L19,USP17L20,USP17L21,USP17L22,USP17L23,USP17L24,USP17L26,USP17L27,USP17L28,USP17L29,USP17L30,USP17L5,USP38
GO:BP	post-translational protein modification	GO:0043687	FALSE	5.63E-11	10.24951294	1021	67	23	21031	RANBP2,SH3RF2,SPSB4,USP17L10,USP17L11,USP17L12,USP17L13,USP17L15,USP17L17,USP17L18,USP17L19,USP17L20,USP17L21,USP17L22,USP17L23,USP17L24,USP17L26,USP17L27,USP17L28,USP17L29,USP17L30,USP17L5,USP38
GO:BP	protein deubiquitination involved in ubiquitin-dependent protein catabolic process	GO:0071947	FALSE	2.98E-10	9.52593954	10	67	6	21031	USP17L24,USP17L26,USP17L27,USP17L28,USP17L29,USP17L30
GO:BP	sensory perception of taste	GO:0050909	TRUE	1.23E-09	8.909289075	67	67	9	21031	TAS2R14,TAS2R19,TAS2R20,TAS2R30,TAS2R31,TAS2R42,TAS2R43,TAS2R46,TAS2R50
GO:BP	detection of chemical stimulus involved in sensory perception of bitter taste	GO:0001580	FALSE	3.97E-08	7.401170044	37	67	7	21031	TAS2R14,TAS2R20,TAS2R30,TAS2R31,TAS2R43,TAS2R46,TAS2R50
GO:BP	sensory perception of bitter taste	GO:0050913	FALSE	1.03E-07	6.988230919	42	67	7	21031	TAS2R14,TAS2R20,TAS2R30,TAS2R31,TAS2R43,TAS2R46,TAS2R50
GO:BP	detection of chemical stimulus involved in sensory perception of taste	GO:0050912	FALSE	1.22E-07	6.912150899	43	67	7	21031	TAS2R14,TAS2R20,TAS2R30,TAS2R31,TAS2R43,TAS2R46,TAS2R50
GO:BP	proteolysis	GO:0006508	TRUE	2.5598E-06	5.591793263	1573	67	22	21031	PSMC4,SH3RF2,SPSB4,USP17L10,USP17L11,USP17L12,USP17L13,USP17L15,USP17L17,USP17L18,USP17L19,USP17L20,USP17L21,USP17L22,USP17L24,USP17L26,USP17L27,USP17L28,USP17L29,USP17L30,USP17L5,USP38

GO:BP	positive regulation of epithelial cell apoptotic process	GO:1904037	TRUE	6.87156E-06	5.162944682	42	67	6	21031	USP17L24,USP17L26,USP17L27,USP17L28,USP17L29,USP17L30
GO:BP	regulation of apoptotic process	GO:0042981	FALSE	2.61927E-05	4.581820448	1463	67	20	21031	ATAD5,SH3RF2,USP17L10,USP17L11,USP17L12,USP17L13,USP17L15,USP17L17,USP17L18,USP17L19,USP17L20,USP17L21,USP17L22,USP17L24,USP17L26,USP17L27,USP17L28,USP17L29,USP17L30,USP17L5
GO:BP	regulation of programmed cell death	GO:0043067	FALSE	4.25743E-05	4.370852427	1506	67	20	21031	ATAD5,SH3RF2,USP17L10,USP17L11,USP17L12,USP17L13,USP17L15,USP17L17,USP17L18,USP17L19,USP17L20,USP17L21,USP17L22,USP17L24,USP17L26,USP17L27,USP17L28,USP17L29,USP17L30,USP17L5
GO:BP	apoptotic process	GO:0006915	FALSE	0.000450639	3.346171422	1913	67	21	21031	ATAD5,EDAR,SH3RF2,USP17L10,USP17L11,USP17L12,USP17L13,USP17L15,USP17L17,USP17L18,USP17L19,USP17L20,USP17L21,USP17L22,USP17L24,USP17L26,USP17L27,USP17L28,USP17L29,USP17L30,USP17L5
GO:BP	programmed cell death	GO:0012501	FALSE	0.000828002	3.081968606	1984	67	21	21031	ATAD5,EDAR,SH3RF2,USP17L10,USP17L11,USP17L12,USP17L13,USP17L15,USP17L17,USP17L18,USP17L19,USP17L20,USP17L21,USP17L22,USP17L24,USP17L26,USP17L27,USP17L28,USP17L29,USP17L30,USP17L5
GO:BP	cell death	GO:0008219	FALSE	0.000856152	3.06744928	1988	67	21	21031	ATAD5,EDAR,SH3RF2,USP17L10,USP17L11,USP17L12,USP17L13,USP17L15,USP17L17,USP17L18,USP17L19,USP17L20,USP17L21,USP17L22,USP17L24,USP17L26,USP17L27,USP17L28,USP17L29,USP17L30,USP17L5
GO:BP	regulation of epithelial cell apoptotic process	GO:1904035	FALSE	0.002368975	2.62543956	110	67	6	21031	USP17L24,USP17L26,USP17L27,USP17L28,USP17L29,USP17L30
GO:BP	epithelial cell apoptotic process	GO:1904019	FALSE	0.009630749	2.016339933	140	67	6	21031	USP17L24,USP17L26,USP17L27,USP17L28,USP17L29,USP17L30

GO:BP	protein modification process	GO:0036211	FALSE	0.018015379	1.744356603	3031	67	24	21031	ERC1,RANBP2,SH3RF2,SPSB4,USP17L10,USP17L11,USP17L12,USP17L13,USP17L15,USP17L17,USP17L18,USP17L19,USP17L20,USP17L21,USP17L22,USP17L23,USP17L24,USP17L26,USP17L27,USP17L28,USP17L29,USP17L30,USP17L5,USP38
GO:BP	macromolecule modification	GO:0043412	FALSE	0.019219394	1.716260303	3262	67	25	21031	ARID4B,ERC1,RANBP2,SH3RF2,SPSB4,USP17L10,USP17L11,USP17L12,USP17L13,USP17L15,USP17L17,USP17L18,USP17L19,USP17L20,USP17L21,USP17L22,USP17L23,USP17L24,USP17L26,USP17L27,USP17L28,USP17L29,USP17L30,USP17L5,USP38
GO:BP	ubiquitin-dependent protein catabolic process	GO:0006511	FALSE	0.032324563	1.490467333	599	67	10	21031	PSMC4,SH3RF2,SPSB4,USP17L24,USP17L26,USP17L27,USP17L28,USP17L29,USP17L30,USP38
GO:BP	modification-dependent protein catabolic process	GO:0019941	FALSE	0.03725046	1.428868364	609	67	10	21031	PSMC4,SH3RF2,SPSB4,USP17L24,USP17L26,USP17L27,USP17L28,USP17L29,USP17L30,USP38
GO:BP	modification-dependent macromolecule catabolic process	GO:0043632	FALSE	0.04340272	1.362483053	620	67	10	21031	PSMC4,SH3RF2,SPSB4,USP17L24,USP17L26,USP17L27,USP17L28,USP17L29,USP17L30,USP38
GO:BP	sensory perception of chemical stimulus	GO:0007606	FALSE	0.046685693	1.330816191	497	67	9	21031	TAS2R14,TAS2R19,TAS2R20,TAS2R30,TAS2R31,TAS2R42,TAS2R43,TAS2R46,TAS2R50
GO:CC	endoplasmic reticulum	GO:0005783	TRUE	0.00010046	3.998007029	2055	67	21	22097	LARS1,PLN,USP17L10,USP17L11,USP17L12,USP17L13,USP17L15,USP17L17,USP17L18,USP17L19,USP17L20,USP17L21,USP17L22,USP17L23,USP17L24,USP17L26,USP17L27,USP17L28,USP17L29,USP17L30,USP17L5
GO:CC	cytosol	GO:0005829	TRUE	0.001139431	2.943312076	5487	67	34	22097	AKAP10,ARID4B,CLASP1,CRLF3,ERC1,GCC2,GGPS1,INPP4B,LARS1,LIMS1,PSMC4,RANBP2,SPECC1,SPSB4,SULT1C4,USP17L10,USP17L11,USP17L12,USP17L13,USP17L15,USP17L17,USP17L18,USP17L19,USP17L20,USP17L21,USP17L22,USP17L24,USP17L26,USP17L27,USP17L28,USP17L29,USP17L30,USP17L5,USP38

KEGG	Taste transduction	KEGG:04742	TRUE	1.13E-11	10.94662962	85	21	9	8484	TAS2R14,TAS2R19,TAS2R20,TAS2R30,TAS2R31,TAS2R42,TAS2R43,TAS2R46,TAS2R50
REAC	Ub-specific processing proteases	REAC:R-HSA-5689880	TRUE	1.15E-18	17.93943936	221	44	19	10842	PSMC4,USP17L10,USP17L11,USP17L12,USP17L13,USP17L15,USP17L17,USP17L18,USP17L19,USP17L20,USP17L21,USP17L22,USP17L24,USP17L26,USP17L27,USP17L28,USP17L29,USP17L30,USP17L5
REAC	Deubiquitination	REAC:R-HSA-5688426	TRUE	3.28E-16	15.48406807	297	44	19	10842	PSMC4,USP17L10,USP17L11,USP17L12,USP17L13,USP17L15,USP17L17,USP17L18,USP17L19,USP17L20,USP17L21,USP17L22,USP17L24,USP17L26,USP17L27,USP17L28,USP17L29,USP17L30,USP17L5
REAC	Class C/3 (Metabotropic glutamate/pheromone receptors)	REAC:R-HSA-420499	FALSE	8.97E-12	11.04734743	39	44	9	10842	TAS2R14,TAS2R19,TAS2R20,TAS2R30,TAS2R31,TAS2R42,TAS2R43,TAS2R46,TAS2R50
REAC	Sensory perception of sweet, bitter, and umami (glutamate) taste	REAC:R-HSA-9717207	TRUE	8.26E-08	7.082803785	41	44	7	10842	TAS2R14,TAS2R20,TAS2R30,TAS2R31,TAS2R43,TAS2R46,TAS2R50
REAC	Sensory perception of taste	REAC:R-HSA-9717189	TRUE	2.27E-07	6.643852968	47	44	7	10842	TAS2R14,TAS2R20,TAS2R30,TAS2R31,TAS2R43,TAS2R46,TAS2R50
REAC	Post-translational protein modification	REAC:R-HSA-597592	FALSE	9.10831E-06	5.040562068	1419	44	21	10842	PSMC4,RANBP2,SPSB4,USP17L10,USP17L11,USP17L12,USP17L13,USP17L15,USP17L17,USP17L18,USP17L19,USP17L20,USP17L21,USP17L22,USP17L24,USP17L26,USP17L27,USP17L28,USP17L29,USP17L30,USP17L5
REAC	Metabolism of proteins	REAC:R-HSA-392499	FALSE	9.361E-05	4.028677871	1946	44	23	10842	ENSG00000285053,LARS1,PSMC4,RANBP2,SPSB4,USP17L10,USP17L11,USP17L12,USP17L13,USP17L15,USP17L17,USP17L18,USP17L19,USP17L20,USP17L21,USP17L22,USP17L24,USP17L26,USP17L27,USP17L28,USP17L29,USP17L30,USP17L5

REAC	G alpha (i) signalling events	REAC:R-HSA-418594	FALSE	0.001303905	2.884754075	312	44	9	10842	TAS2R14,TAS2R19,TAS2R20,TAS2R30,TAS2R31,TAS2R42,TAS2R43,TAS2R46,TAS2R50
REAC	GPCR ligand binding	REAC:R-HSA-500792	FALSE	0.028723338	1.541765097	460	44	9	10842	TAS2R14,TAS2R19,TAS2R20,TAS2R30,TAS2R31,TAS2R42,TAS2R43,TAS2R46,TAS2R50

Table S2B. Detailed summary statistics of Functional Enrichment Analysis within Runs of Homozygosity (ROH) Islands. This table presents the detailed statistics of functional enrichment analysis on genes identified within runs of homozygosity (ROH) islands (> 100 KB), detected in the BirThree dataset via PLINK (by setting 99.9th percentile threshold based on the frequencies of overlapping ROH_{100} regions shared among individuals). The gProfiler tool was utilized to identify enriched biological pathways (BP), molecular functions (MF), and cellular components (CC) from Gene Ontology (GO), KEGG, and Reactome. The intersections column displays the genes from the query list that overlap with each functional term, contributing to its statistical significance. Detailed information regarding statistics can be found on gProfiler website.

source	term_name	term_id	highlighted	adjusted_p_value	negative_log10_of_adjusted_p_value	term_size	query_size	intersection_size	effective_domain_size	intersections
GO:MF	3-hydroxyacyl-CoA dehydrogenase activity	GO:0003857	TRUE	0.002935338	2.532341826	7	105	3	20212	HADHA,HADHB,HSD17B12
GO:MF	cysteine desulfurase activity	GO:0031071	TRUE	0.016707273	1.777094436	2	105	2	20212	ENSG00000272897,NFS1
GO:MF	long-chain-3-hydroxyacyl-CoA dehydrogenase activity	GO:0016509	FALSE	0.049951522	1.301451278	3	105	2	20212	HADHA,HSD17B12
GO:MF	vitamin-K-epoxide reductase (warfarin-sensitive) activity	GO:0047057	TRUE	0.049951522	1.301451278	3	105	2	20212	ENSG00000255439,VKORC1
GO:CC	mitochondrial fatty acid beta-oxidation multienzyme complex	GO:0016507	TRUE	0.024690922	1.607462701	3	109	2	22097	HADHA,HADHB
GO:CC	fatty acid beta-oxidation multienzyme complex	GO:0036125	FALSE	0.024690922	1.607462701	3	109	2	22097	HADHA,HADHB

KEGG	Fatty acid elongation	KEGG:00062	TRUE	0.009873472	2.005530103	27	32	3	8484	HADHA,HADHB,HSD17B12
REAC	Beta oxidation of myristoyl-CoA to lauroyl-CoA	REAC:R-HSA-77285	TRUE	0.025043751	1.60130063	3	53	2	10842	HADHA,HADHB
REAC	Beta oxidation of palmitoyl-CoA to myristoyl-CoA	REAC:R-HSA-77305	FALSE	0.025043751	1.60130063	3	53	2	10842	HADHA,HADHB

Table S2C. Detailed summary statistics of Functiional Enrichment Analysis within Runs of Homozygosity (ROH) Islands. This table presents the detailed statistics of functional enrichment analysis on genes identified within runs of homozygosity (ROH) islands (> 100 KB), detected in the 3.5KJPNv2 dataset via PLINK (by setting 99.9th percentile threshold based on the frequencies of overlapping ROH_{100} regions shared among individuals). The gProfiler tool was utilized to identify enriched biological pathways (BP), molecular functions (MF), and cellular components (CC) from Gene Ontology (GO), KEGG, and Reactome. The intersections column displays the genes from the query list that overlap with each functional term, contributing to its statistical significance. Detailed information regarding statistics can be found on gProfiler website.

source	term_name	term_id	highlighted	adjusted_p_value	negative_log10_of_adjusted_p_value	term_size	query_size	intersection_size	effective_domain_size	intersections
GO:MF	vitamin-K-epoxide reductase (warfarin-sensitive) activity	GO:0047057	TRUE	0.049953	1.301437	3	101	2	20212	ENSG00000255439,VKORC1

Table S2D. Detailed summary statistics of Functional Enrichment Analysis within Runs of Homozygosity (ROH) Islands. This table presents the detailed statistics of functional enrichment analysis on genes identified within runs of homozygosity (ROH) islands (> 1.5 MB), detected in the BirThree dataset via BCFtools (by setting 99.5th percentile threshold based on the frequencies of overlapping ROH_{1500} regions shared among individuals). The gProfiler tool was utilized to identify enriched biological pathways (BP), molecular functions (MF), and cellular components (CC) from Gene Ontology (GO), KEGG, and Reactome. The intersections column displays the genes from the query list that overlap with each functional term, contributing to its statistical significance. Detailed information regarding statistics can be found on gProfiler website.

source	term_name	term_id	highlighted	adjusted_p_value	negative_log10_of_adjusted_p_value	term_size	query_size	intersection_size	effective_domain_size	intersections
GO:MF	olfactory receptor activity	GO:0004984	TRUE	8.17E-09	8.087860736	397	19	9	20212	OR4A47,OR4B1,OR4C12,OR4C13,OR4C3,OR4C5,OR4S1,OR4X1,OR4X2
GO:MF	G protein-coupled receptor activity	GO:0004930	FALSE	5.83018E-06	5.234318358	838	19	9	20212	OR4A47,OR4B1,OR4C12,OR4C13,OR4C3,OR4C5,OR4S1,OR4X1,OR4X2
GO:MF	transmembrane signaling receptor activity	GO:0004888	FALSE	0.000202748	3.69304294	1269	19	9	20212	OR4A47,OR4B1,OR4C12,OR4C13,OR4C3,OR4C5,OR4S1,OR4X1,OR4X2
GO:MF	molecular transducer activity	GO:0060089	FALSE	0.000782508	3.10651107	1491	19	9	20212	OR4A47,OR4B1,OR4C12,OR4C13,OR4C3,OR4C5,OR4S1,OR4X1,OR4X2
GO:MF	signaling receptor activity	GO:0038023	FALSE	0.000782508	3.10651107	1491	19	9	20212	OR4A47,OR4B1,OR4C12,OR4C13,OR4C3,OR4C5,OR4S1,OR4X1,OR4X2
GO:BP	detection of chemical stimulus involved in sensory perception of smell	GO:0050911	TRUE	8.70E-09	8.060442621	397	17	9	21031	OR4A47,OR4B1,OR4C12,OR4C13,OR4C3,OR4C5,OR4S1,OR4X1,OR4X2
GO:BP	sensory perception of smell	GO:0007608	FALSE	1.53E-08	7.813969769	423	17	9	21031	OR4A47,OR4B1,OR4C12,OR4C13,OR4C3,OR4C5,OR4S1,OR4X1,OR4X2
GO:BP	detection of chemical stimulus involved in sensory perception	GO:0050907	FALSE	2.32E-08	7.634734984	443	17	9	21031	OR4A47,OR4B1,OR4C12,OR4C13,OR4C3,OR4C5,OR4S1,OR4X1,OR4X2
GO:BP	detection of chemical stimulus	GO:0009593	FALSE	4.74E-08	7.324048079	480	17	9	21031	OR4A47,OR4B1,OR4C12,OR4C13,OR4C3,OR4C5,OR4S1,OR4X1,OR4X2
GO:BP	sensory perception of chemical stimulus	GO:0007606	FALSE	6.46E-08	7.1894626	497	17	9	21031	OR4A47,OR4B1,OR4C12,OR4C13,OR4C3,OR4C5,OR4S1,OR4X1,OR4X2
GO:BP	detection of stimulus involved in sensory perception	GO:0050906	FALSE	8.87E-08	7.052024764	515	17	9	21031	OR4A47,OR4B1,OR4C12,OR4C13,OR4C3,OR4C5,OR4S1,OR4X1,OR4X2
GO:BP	detection of stimulus	GO:0051606	FALSE	6.61E-07	6.179920045	646	17	9	21031	OR4A47,OR4B1,OR4C12,OR4C13,OR4C3,OR4C5,OR4S1,OR4X1,OR4X2

GO:BP	sensory perception	GO:0007600	FALSE	1.99769E-05	4.699471336	953	17	9	21031	OR4A47,OR4B1,OR4C12,OR4C13,OR4C3,OR4C5,OR4S1,OR4X1,OR4X2
GO:BP	G protein-coupled receptor signaling pathway	GO:0007186	TRUE	0.000256936	3.590175775	1281	17	9	21031	OR4A47,OR4B1,OR4C12,OR4C13,OR4C3,OR4C5,OR4S1,OR4X1,OR4X2
GO:BP	nervous system process	GO:0050877	FALSE	0.000900599	3.045468541	1484	17	9	21031	OR4A47,OR4B1,OR4C12,OR4C13,OR4C3,OR4C5,OR4S1,OR4X1,OR4X2
GO:BP	system process	GO:0003008	FALSE	0.029819408	1.525500978	2258	17	9	21031	OR4A47,OR4B1,OR4C12,OR4C13,OR4C3,OR4C5,OR4S1,OR4X1,OR4X2
KEGG	Olfactory transduction	KEGG:04740	TRUE	1.14E-09	8.941297157	428	10	9	8484	OR4A47,OR4B1,OR4C12,OR4C13,OR4C3,OR4C5,OR4S1,OR4X1,OR4X2
REAC	Expression and translocation of olfactory receptors	REAC:R-HSA-9752946	TRUE	1.02E-10	9.991767365	354	10	9	10842	OR4A47,OR4B1,OR4C12,OR4C13,OR4C3,OR4C5,OR4S1,OR4X1,OR4X2
REAC	Olfactory Signaling Pathway	REAC:R-HSA-381753	TRUE	1.22E-10	9.914621786	361	10	9	10842	OR4A47,OR4B1,OR4C12,OR4C13,OR4C3,OR4C5,OR4S1,OR4X1,OR4X2
REAC	Sensory Perception	REAC:R-HSA-9709957	TRUE	8.19E-09	8.086876154	575	10	9	10842	OR4A47,OR4B1,OR4C12,OR4C13,OR4C3,OR4C5,OR4S1,OR4X1,OR4X2

Table S2E. Detailed summary statistics of Functional Enrichment Analysis within Runs of Homozygosity (ROH) Islands. This table presents the detailed statistics of functional enrichment analysis on genes identified within runs of homozygosity (ROH) islands (> 1.5 MB), detected in the 3.5KJPNv2 dataset via BCFtools (by setting 99.5th percentile threshold based on the frequencies of overlapping ROH_{1500} regions shared among individuals). The gProfiler tool was utilized to identify enriched biological pathways (BP), molecular functions (MF), and cellular components (CC) from Gene Ontology (GO), KEGG, and Reactome. The intersections column displays the genes from the query lists that overlap with each functional term, contributing to its statistical significance. Detailed information regarding statistics can be found on gProfiler website.

source	term_name	term_id	highlighted	adjusted_p_value	negative_log10_of_adjusted_p_value	term_size	query_size	intersection_size	effective_domain_size	intersections
GO:MF	olfactory receptor activity	GO:0004984	TRUE	1.16359E-05	4.934201324	397	19	7	20212	OR4A47,OR4B1,OR4C3,OR4C5,OR4S1,OR4X1,OR4X2
GO:MF	G protein-coupled receptor activity	GO:0004930	FALSE	0.001767715	2.752587866	838	19	7	20212	OR4A47,OR4B1,OR4C3,OR4C5,OR4S1,OR4X1,OR4X2
GO:MF	transmembrane signaling receptor activity	GO:0004888	FALSE	0.025801957	1.58834736	1269	19	7	20212	OR4A47,OR4B1,OR4C3,OR4C5,OR4S1,OR4X1,OR4X2
GO:BP	detection of chemical stimulus involved in sensory perception of smell	GO:0050911	TRUE	4.79621E-05	4.319101915	397	19	7	21031	OR4A47,OR4B1,OR4C3,OR4C5,OR4S1,OR4X1,OR4X2
GO:BP	detection of stimulus	GO:0051606	FALSE	6.07501E-05	4.216453048	646	19	8	21031	OR4A47,OR4B1,OR4C3,OR4C5,OR4S1,OR4X1,OR4X2,PTPRJ
GO:BP	sensory perception of smell	GO:0007608	FALSE	7.40381E-05	4.130545027	423	19	7	21031	OR4A47,OR4B1,OR4C3,OR4C5,OR4S1,OR4X1,OR4X2

GO:BP	detection of chemical stimulus involved in sensory perception	GO:0050907	FALSE	0.000101503	3.993521188	443	19	7	21031	OR4A47,OR4B1,OR4C3,OR4C5,OR4S1,OR4X1,OR4X2
GO:BP	detection of chemical stimulus	GO:0009593	FALSE	0.000175303	3.756209969	480	19	7	21031	OR4A47,OR4B1,OR4C3,OR4C5,OR4S1,OR4X1,OR4X2
GO:BP	sensory perception of chemical stimulus	GO:0007606	FALSE	0.000222076	3.65349779	497	19	7	21031	OR4A47,OR4B1,OR4C3,OR4C5,OR4S1,OR4X1,OR4X2
GO:BP	detection of stimulus involved in sensory perception	GO:0050906	FALSE	0.000282704	3.548667552	515	19	7	21031	OR4A47,OR4B1,OR4C3,OR4C5,OR4S1,OR4X1,OR4X2
GO:BP	sensory perception	GO:0007600	FALSE	0.01710409	1.766900033	953	19	7	21031	OR4A47,OR4B1,OR4C3,OR4C5,OR4S1,OR4X1,OR4X2
KEGG	Olfactory transduction	KEGG:04740	TRUE	5.11334E-06	5.291295137	428	10	7	8484	OR4A47,OR4B1,OR4C3,OR4C5,OR4S1,OR4X1,OR4X2
REAC	Expression and translocation of olfactory receptors	REAC:R-HSA-9752946	TRUE	7.50245E-06	5.124796741	354	12	7	10842	OR4A47,OR4B1,OR4C3,OR4C5,OR4S1,OR4X1,OR4X2
REAC	Olfactory Signaling Pathway	REAC:R-HSA-381753	TRUE	8.58965E-06	5.066024533	361	12	7	10842	OR4A47,OR4B1,OR4C3,OR4C5,OR4S1,OR4X1,OR4X2
REAC	Sensory Perception	REAC:R-HSA-9709957	TRUE	0.00020879	3.680290025	575	12	7	10842	OR4A47,OR4B1,OR4C3,OR4C5,OR4S1,OR4X1,OR4X2

Table S2F. Detailed summary statistics of Functional Enrichment Analysis within Runs of Homozygosity (ROH) Islands. This table presents the detailed statistics of functional enrichment analysis on genes identified within runs of homozygosity (ROH) islands (> 1.5 MB), detected in the 3.5KJPNv2 dataset via PLINK (by setting 99.5th percentile threshold based on the frequencies of overlapping ROH_{1500} regions shared among individuals). The gProfiler tool was utilized to identify enriched biological pathways (BP), molecular functions (MF), and cellular components (CC) from Gene Ontology (GO), KEGG, and Reactome. The intersections column displays the genes from the query list that overlap with each functional term, contributing to its statistical significance. Detailed information regarding statistics can be found on gProfiler website.

source	term_name	term_id	highlighted	adjusted_p_value	negative_log10_of_adjusted_p_value	term_size	query_size	intersection_size	effective_domain_size	intersections
GO:MF	aminomethyltransferase activity	GO:0004047	TRUE	0.010263084	1.988722098	3	56	2	20212	AMT,ENSG00000283189
GO:MF	inositol 5-diphosphate pentakisphosphate 5-kinase activity	GO:0052836	TRUE	0.010263084	1.988722098	3	56	2	20212	IP6K1,IP6K2
GO:MF	inositol diphosphate tetrakisphosphate kinase activity	GO:0052839	TRUE	0.010263084	1.988722098	3	56	2	20212	IP6K1,IP6K2
GO:MF	IMP dehydrogenase activity	GO:0003938	TRUE	0.020489612	1.688466255	4	56	2	20212	ENSG00000290315,IMPDH2
GO:MF	inositol-1,3,4,5,6-pentakisphosphate kinase activity	GO:0000827	TRUE	0.034088547	1.467391515	5	56	2	20212	IP6K1,IP6K2
GO:MF	inositol hexakisphosphate kinase activity	GO:0000828	FALSE	0.034088547	1.467391515	5	56	2	20212	IP6K1,IP6K2
GO:MF	inositol heptakisphosphate kinase activity	GO:0000829	TRUE	0.034088547	1.467391515	5	56	2	20212	IP6K1,IP6K2
GO:MF	inositol hexakisphosphate 5-kinase activity	GO:0000832	TRUE	0.034088547	1.467391515	5	56	2	20212	IP6K1,IP6K2
REAC	Synthesis of IPs in the nucleus	REAC:R-HSA-1855191	TRUE	0.020033096	1.698251925	4	35	2	10842	IP6K1,IP6K2
