# Supplementary material for: Profiling of runs of homozygosity from whole-genome sequence data in Japanese biobank
Source: J Hum Genet. 2025 Apr 3;70(6):287–96. doi: 10.1038/s10038-025-01331-3 (PMC12058513; doi:10.1038/s10038-025-01331-3)

Figure S1A. Genome-wide ROH distribution patterns in 3.5KJPNv2 dataset (on Chromosome 6 only). ROH segments longer than 1.5 Mb are inferred as red bars. ROH segments between 100 Kb and 1.5 Mb are inferred as black bars. Horizontal axis represents the base pair distances of ROHs detected by BCFtools on chromosome 6. Vertical axis represents each individual in the 3.5KJPNv2 dataset.

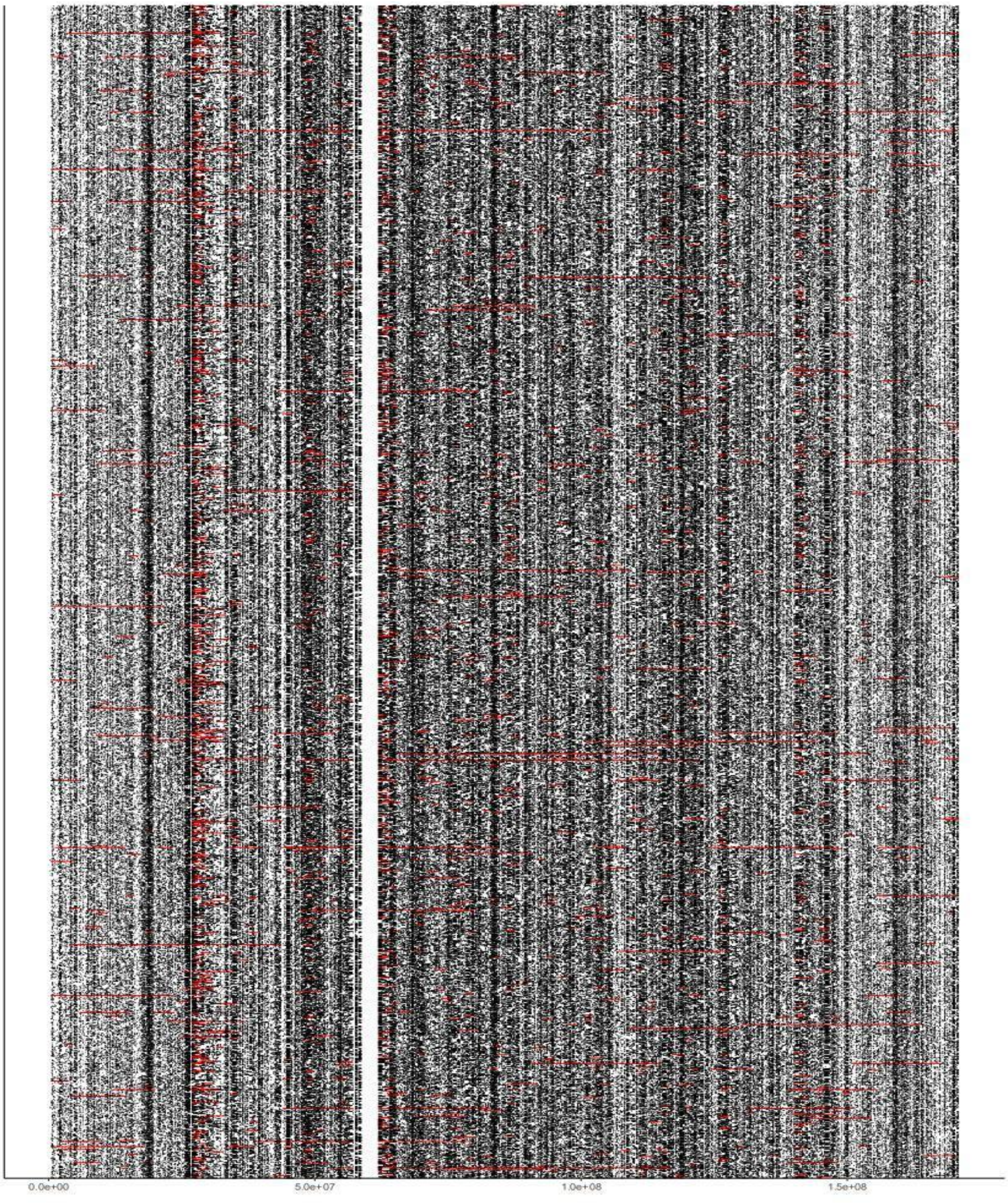

Figure S1B. OmniExpressExome array-based sites specific ROH distribution patterns in 3.5KJPNv2 dataset (on Chromosome 6 only). ROH segments longer than 1.5 Mb are inferred as red bars. ROH segments between 100 Kb and 1.5 Mb are inferred as black bars. Horizontal axis represents the base pair distances of ROHs detected by BCFtools on chromosome 6. Vertical axis represents each individual in the 3.5KJPNv2 dataset.

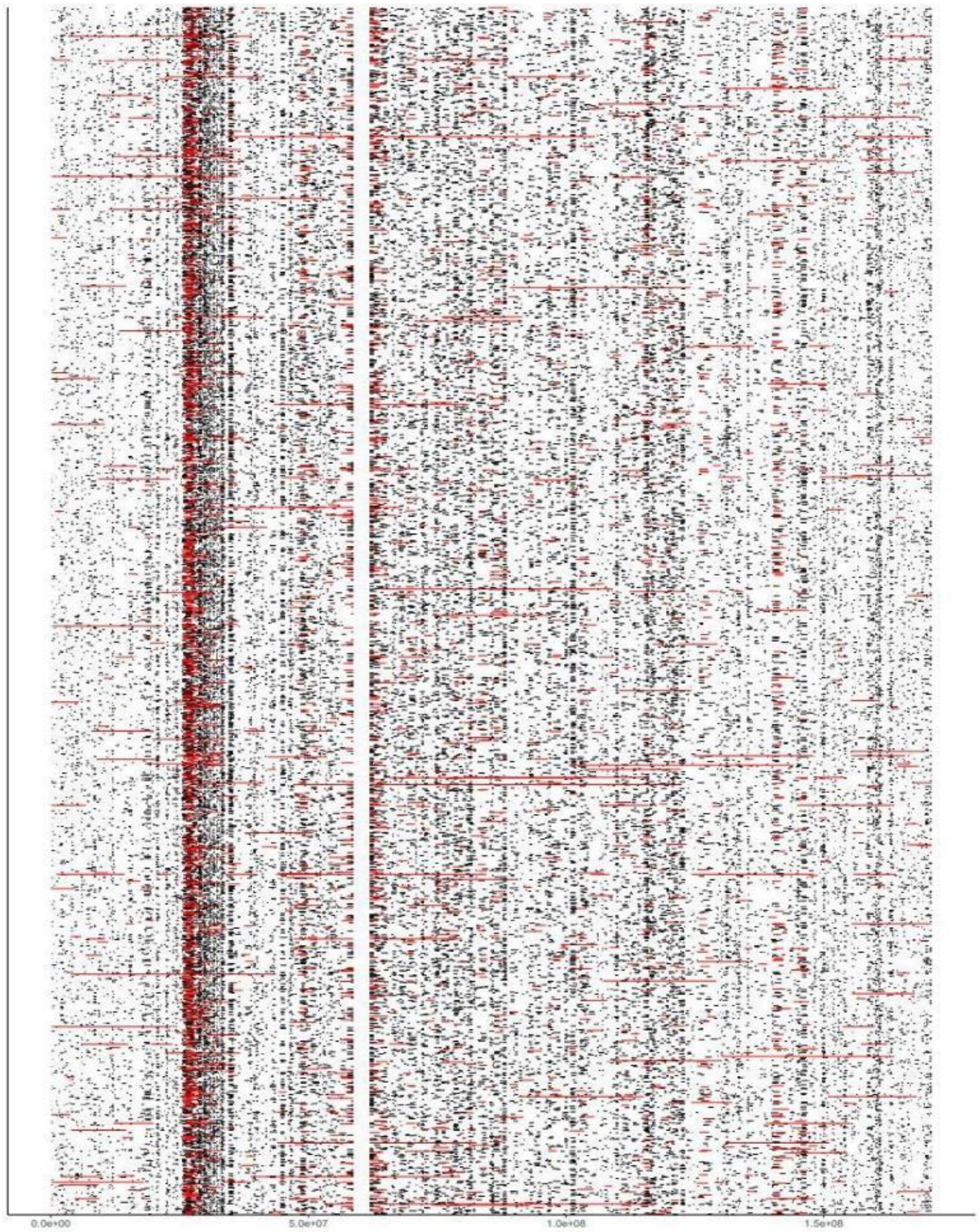

Supplement: Supplementary file 4 — Genomic Distributions of ROHs per individual (on chromosome 6 in 3.5KJPNv2 dataset only) [file 10038_2025_1331_MOESM4_ESM.pdf]
