# Supplementary material for: Profiling of runs of homozygosity from whole-genome sequence data in Japanese biobank
Source: J Hum Genet. 2025 Apr 3;70(6):287–96. doi: 10.1038/s10038-025-01331-3 (PMC12058513; doi:10.1038/s10038-025-01331-3)

Figure S1C. ROH island distribution patterns in BirThree and 3.5KJPNv2 dataset (ROH > 100 KB) (BCFtools). ROH islands are identified in both BirThree and 3.5KJPNv2 datasets by selecting genomic regions based on the frequencies of overlapping ROH regions (for ROHs > 100 KB detected via BCFtools) shared among individuals, using a 99.9th percentile threshold. ROH islands identified in 3.5KJPNv2 dataset are inferred as red bars and those in BirThree datasets are inferred as blue bars. Horizontal axis represents the genomic positions. Vertical axis represents chromosomes in each dataset.

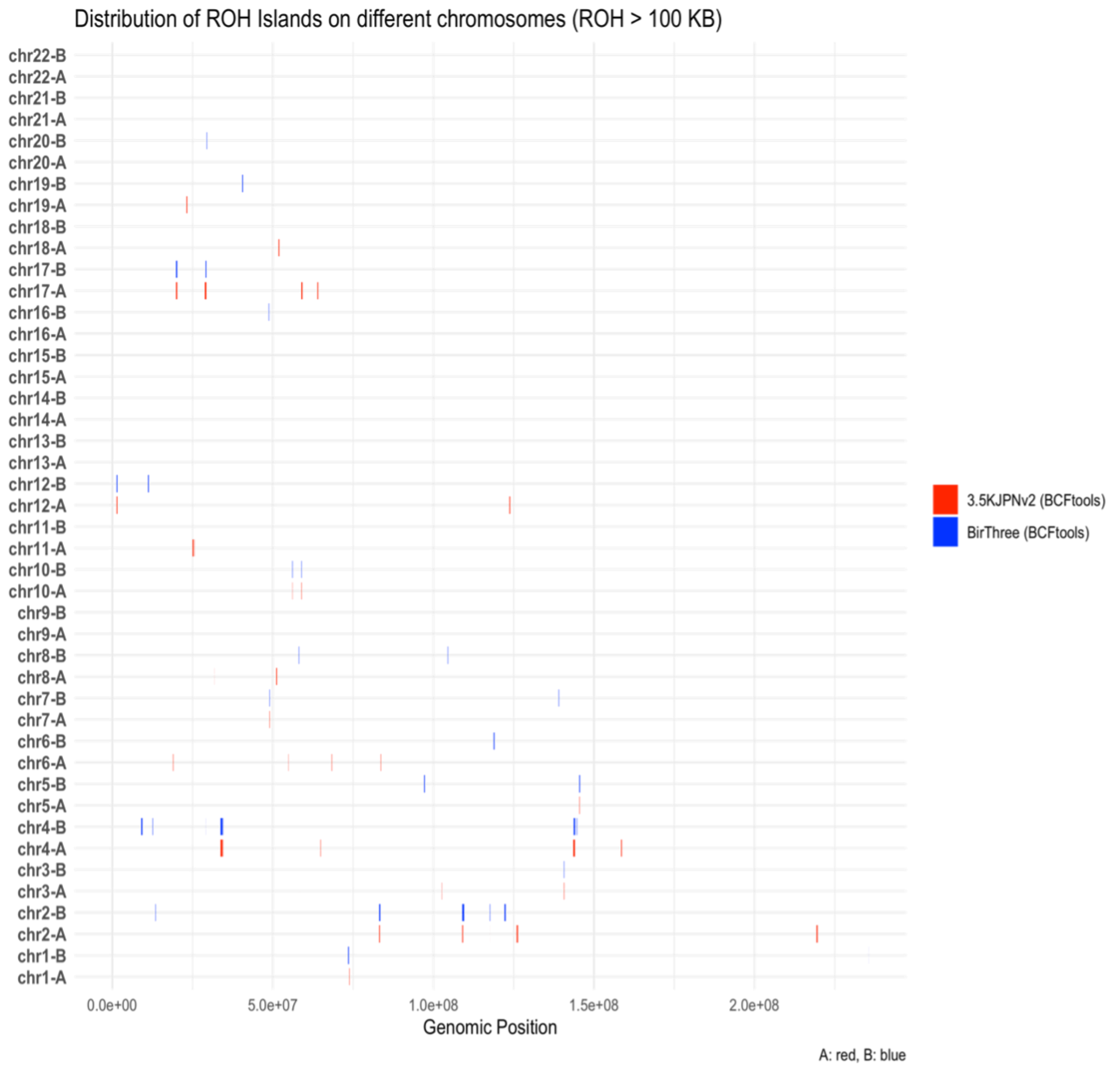

Figure S1D. ROH island distribution patterns in BirThree and 3.5KJPNv2 dataset (ROH > 1.5 MB) (BCFtools). ROH islands are identified in both BirThree and 3.5KJPNv2 datasets by selecting genomic regions based on the frequencies of overlapping ROH regions (for ROHs > 1.5 MB detected via BCFtools) shared among individuals, using a 99.5th percentile threshold. ROH islands identified in 3.5KJPNv2 dataset are inferred as red bars and those in BirThree datasets are inferred as blue bars. Horizontal axis represents the genomic positions. Vertical axis represents chromosomes in each dataset.

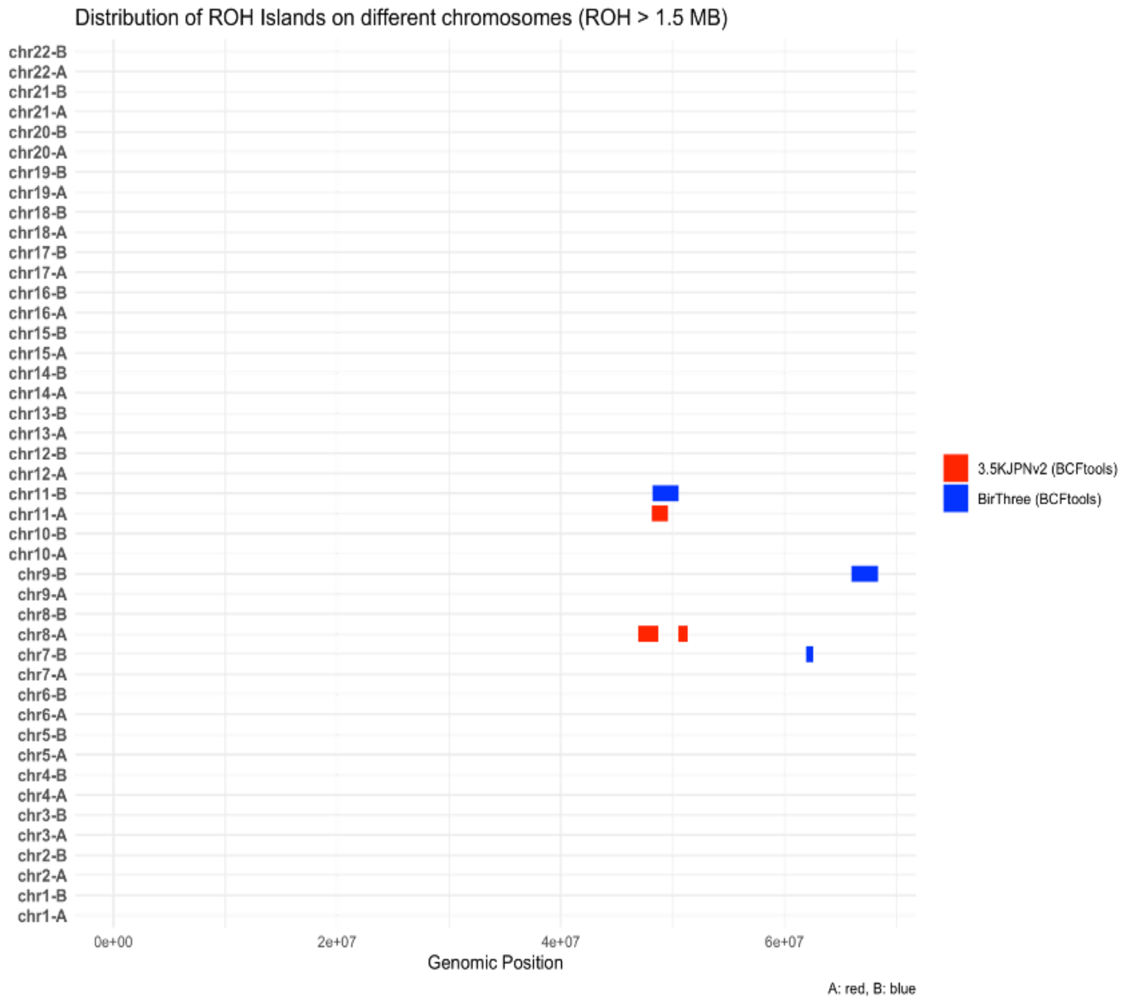

Figure S1E. Comparisons of ROH island distribution patterns between PLINK and BCFtools (ROH > 100 KB) in BirThree dataset. ROH islands are identified in BirThree datasets by selecting genomic regions based on the frequencies of overlapping ROH regions (for ROHs > 100 KB detected via PLINK and BCFtools) shared among individuals, using a 99.9th percentile threshold. ROH islands identified by BCFtools are inferred as red bars and those identified by PLINK are inferred as blue bars. Horizontal axis represents the genomic positions. Vertical axis represents chromosomes in each dataset.

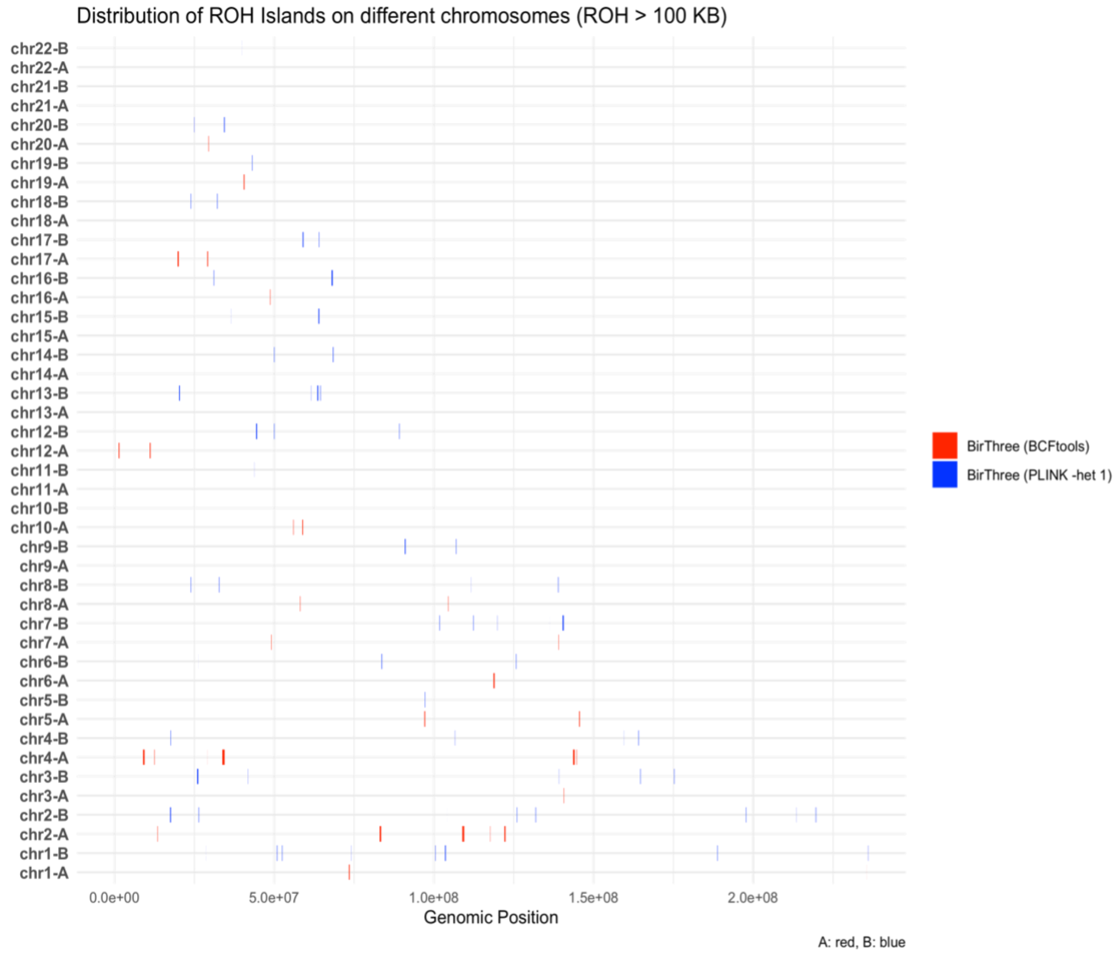

Figure S1F. Comparisons of ROH island distribution patterns between PLINK and BCFtools (ROH > 100 KB) in 3.5KJPNv2 dataset. ROH islands are identified in 3.5KJPNv2 datasets by selecting genomic regions based on the frequencies of overlapping ROH regions (for ROHs > 100 KB detected via PLINK and BCFtools) shared among individuals, using a 99.9th percentile threshold. ROH islands identified by BCFtools are inferred as red bars and those identified by PLINK are inferred as blue bars. Horizontal axis represents the genomic positions. Vertical axis represents chromosomes in each dataset.

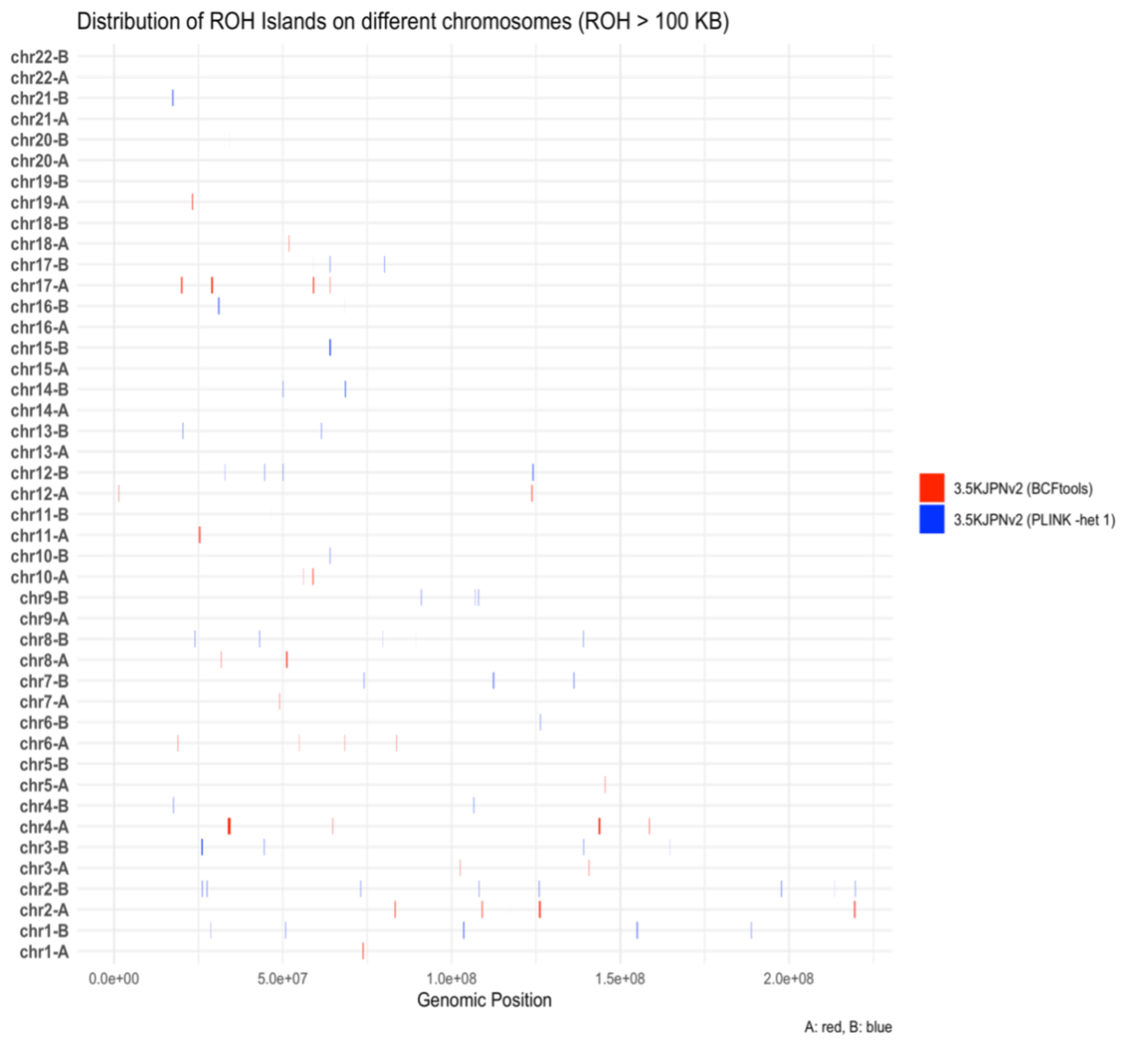

Figure S1G. ROH island distribution patterns in BirThree and 3.5KJPNv2 dataset (ROH > 1.5 MB) (PLINK). ROH islands are identified in both BirThree and 3.5KJPNv2 datasets by selecting genomic regions based on the frequencies of overlapping ROH regions (for ROHs > 1.5 MB detected via PLINK) shared among individuals, using a 99.5th percentile threshold. ROH islands identified in 3.5KJPNv2 dataset are inferred as red bars and those in BirThree datasets are inferred as blue bars. Horizontal axis represents the genomic positions. Vertical axis represents chromosomes in each dataset.

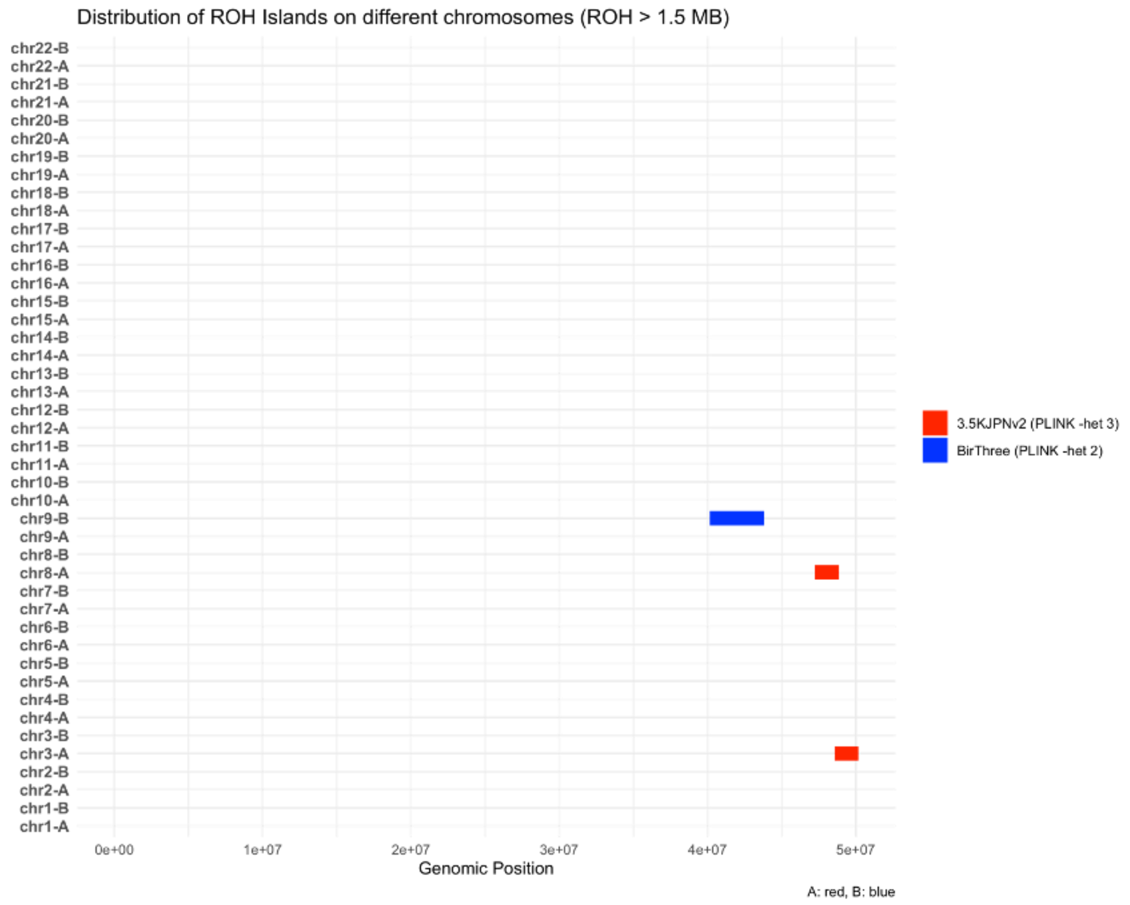

Supplement: Supplementary file 5 — Genomic Distributions of ROH islands on autosomes [file 10038_2025_1331_MOESM5_ESM.pdf]
