# Supplementary material for: Profiling of runs of homozygosity from whole-genome sequence data in Japanese biobank
Source: J Hum Genet. 2025 Apr 3;70(6):287–96. doi: 10.1038/s10038-025-01331-3 (PMC12058513; doi:10.1038/s10038-025-01331-3)
Supplement: Supplementary file 6 — Full figures for Functional Enrichment Analysis of Annotated Genes within ROH islands [file 10038_2025_1331_MOESM6_ESM.pdf]

Figure S2A. Functional Enrichment Analysis of Annotated Genes within Runs of Homozygosity (ROH) Islands (>100 KB)**.** This figure presents functional enrichment analysis on genes identified within runs of homozygosity (ROH) islands, detected in the BirThree and 3.KJPNv2 datasets via BCFtools and PLINK (by setting 99.9th percentile threshold based on the frequencies of overlapping ROH_{100} regions shared among individuals). The gProfiler was used to identify enriched biological pathways (BP), molecular functions (MF), and cellular components (CC) from Gene Ontology (GO), KEGG, and Reactome. The y-axis displays the enrichment score, indicating statistical significance, while the x-axis and color coding represent the data source. Dot size corresponds to the number of genes associated with each term. A summary of comparative statistics with other datasets is also provided.

BirThree (BCFtools) (ROH > 100 KB) (99.9th percentile)

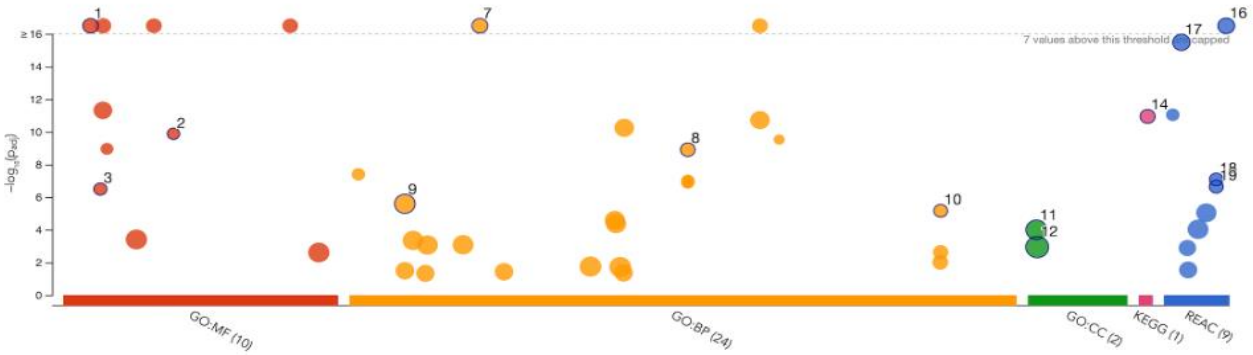

3.5KJPNv2 (BCFtools) (ROH > 100 KB) (99.9th percentile)

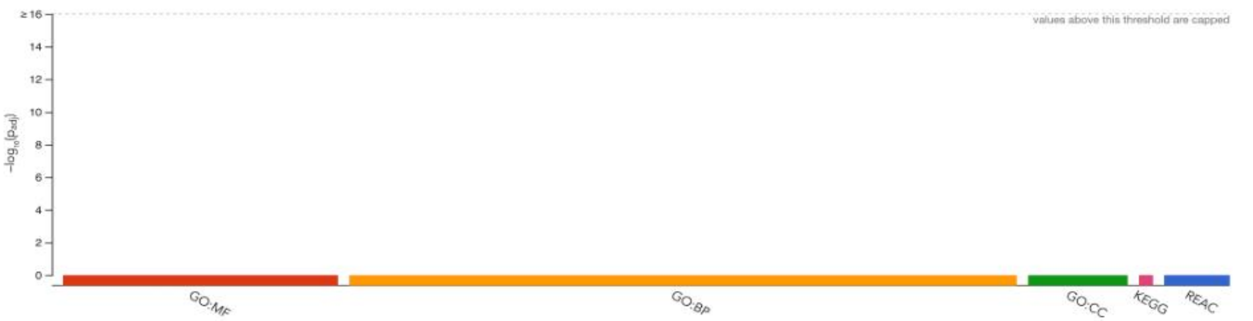

BirThree (PLINK -het 1) (ROH > 100 KB) (99.9th percentile)

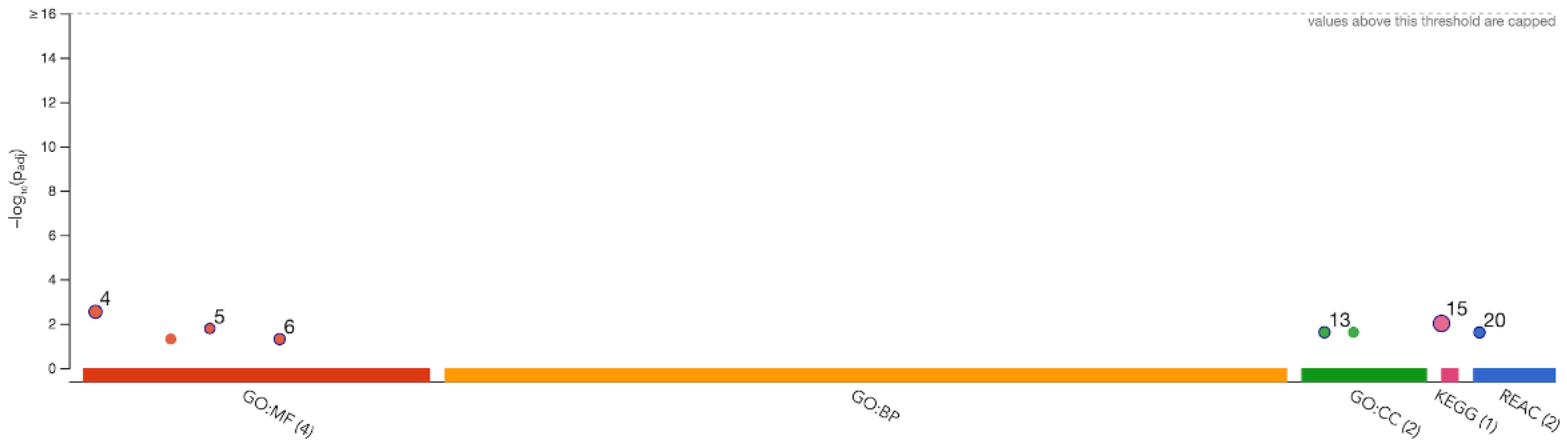

3.5KJPNv2 (PLINK -het 1) (ROH > 100 KB) (99.9th percentile)

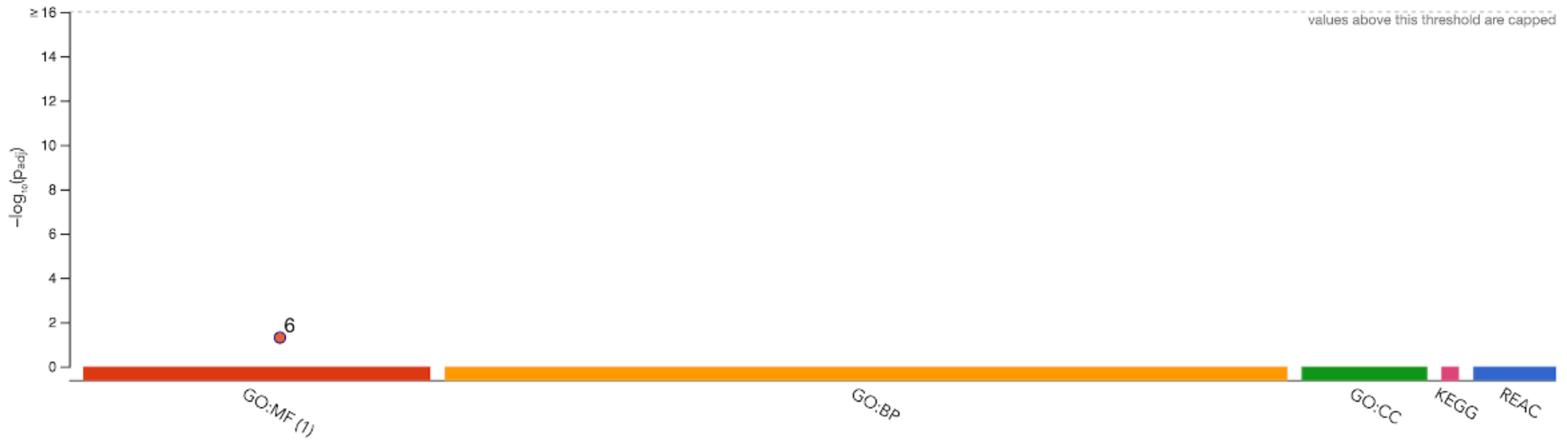

ID	Source	Term ID	Term Name	p_{adj} (BirThree (BCFtool...	p_{adj} (3.5KJPNv2 (BCF...	p_{adj} (BirThree (PLI...	p_{adj} (3.5KJPNv2 (PLI...
1	GO:MF	GO:0004843	cysteine-type deubiquitinase activity	1.049×10^{-26}	~~1.000~~	~~1.000~~	~~1.000~~
2	GO:MF	GO:0033038	bitter taste receptor activity	1.316×10^{-10}			
3	GO:MF	GO:0005540	hyaluronic acid binding	3.192×10^{-7}			
4	GO:MF	GO:0003857	3-hydroxyacyl-CoA dehydrogenase activity			2.935×10^{-3}	
5	GO:MF	GO:0031071	cysteine desulfurase activity			1.671×10^{-2}	
6	GO:MF	GO:0047057	vitamin-K-epoxide reductase (warfarin-s...			4.995×10^{-2}	4.995×10^{-2}
7	GO:BP	GO:0016579	protein deubiquitination	4.895×10^{-26}	~~1.000~~	~~1.000~~	~~1.000~~
8	GO:BP	GO:0050909	sensory perception of taste	1.232×10^{-9}			
9	GO:BP	GO:0006508	proteolysis	2.560×10^{-6}	~~1.000~~	~~1.000~~	~~1.000~~
10	GO:BP	GO:1904037	positive regulation of epithelial cell apopt...	6.872×10^{-6}			
11	GO:CC	GO:0005783	endoplasmic reticulum	1.005×10^{-4}	~~1.000~~	~~1.000~~	~~1.000~~
12	GO:CC	GO:0005829	cytosol	1.139×10^{-3}	~~1.000~~	~~1.000~~	~~1.000~~
13	GO:CC	GO:0016507	mitochondrial fatty acid beta-oxidation m...			2.469×10^{-2}	
14	KEGG	KEGG:04742	Taste transduction	1.131×10^{-11}			
15	KEGG	KEGG:00062	Fatty acid elongation			9.873×10^{-3}	
16	REAC	REAC:R-HS...	Ub-specific processing proteases	1.150×10^{-18}	~~1.000~~	~~1.000~~	~~1.000~~
17	REAC	REAC:R-HS...	Deubiquitination	3.280×10^{-16}	~~1.000~~	~~1.000~~	~~1.000~~
18	REAC	REAC:R-HS...	Sensory perception of sweet, bitter, and ...	8.264×10^{-8}			
19	REAC	REAC:R-HS...	Sensory perception of taste	2.271×10^{-7}			
20	REAC	REAC:R-HS...	Beta oxidation of palmitoyl-CoA to myrist...			2.504×10^{-2}	

version e111_eg58_p18_f463989d
date 10/16/2024, 11:56:29 AM
organism hsapiens

g:Profiler

Figure S2B. Functional Enrichment Analysis of Annotated Genes within Runs of Homozygosity (ROH) Islands (> 1.5 MB)**.** This figure presents functional enrichment analysis on genes identified within runs of homozygosity (ROH) islands, detected in the BirThree and 3.KJPNv2 datasets via BCFtools and PLINK (by setting 99.5th percentile threshold based on the frequencies of overlapping ROH_{1500} regions shared among individuals). The gProfiler tool was used to identify enriched biological pathways (BP), molecular functions (MF), and cellular components (CC) from Gene Ontology (GO), KEGG, and Reactome. The y-axis displays the enrichment score, indicating statistical significance, while the x-axis and color coding represent the data source. Dot size corresponds to the number of genes associated with each term. A summary of comparative statistics with other datasets is also provided.

> BirThree (BCFtools) (ROH > 1.5 MB) (99.5th percentile)

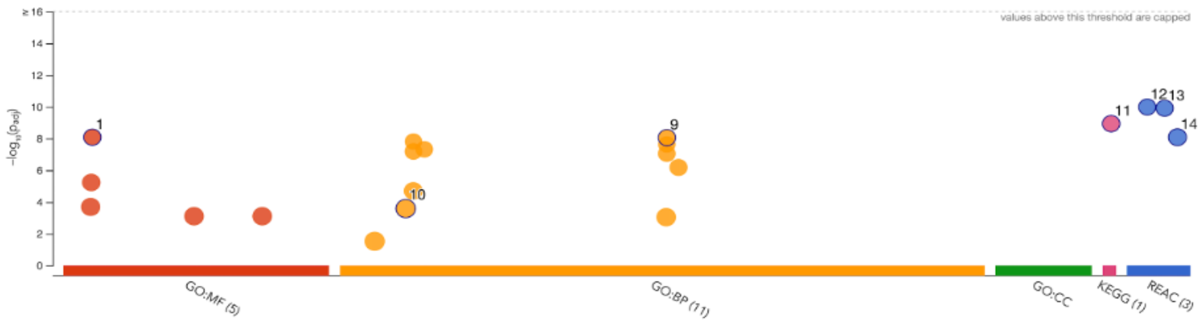

> 3.5KJPNv2 (BCFtools) (ROH > 1.5 MB) (99.5th percentile)

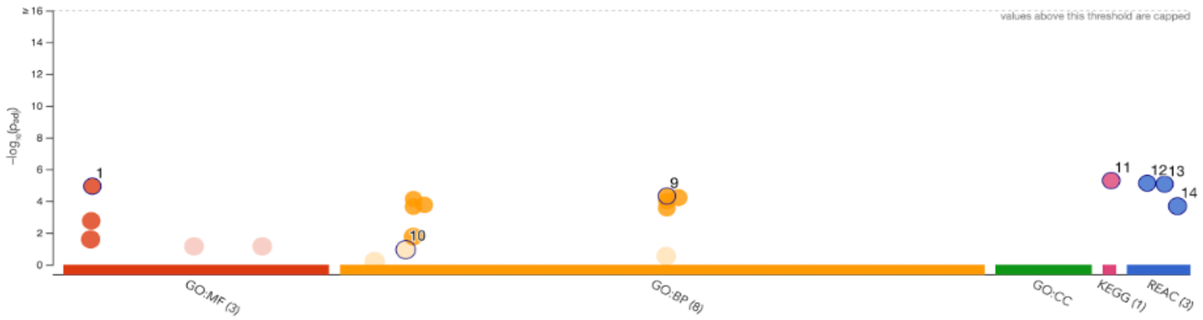

> BirThree (PLINK -het 2) (ROH > 1.5 MB) (99.5th percentile)

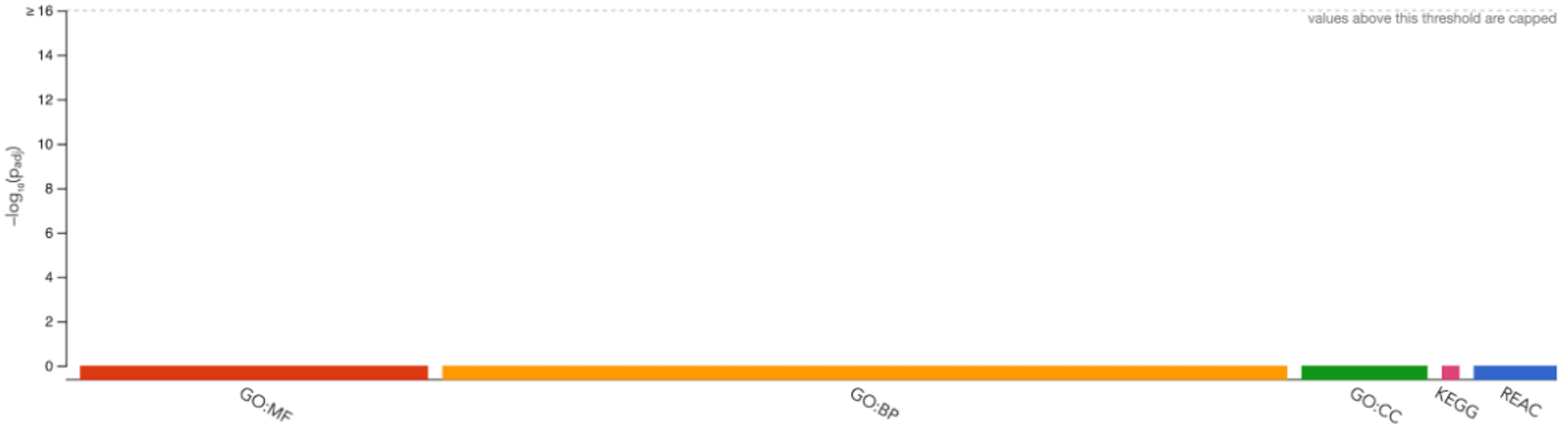

> 3.5KJPNv2 (PLINK -het 3) (ROH > 1.5 MB) (99.5th percentile)

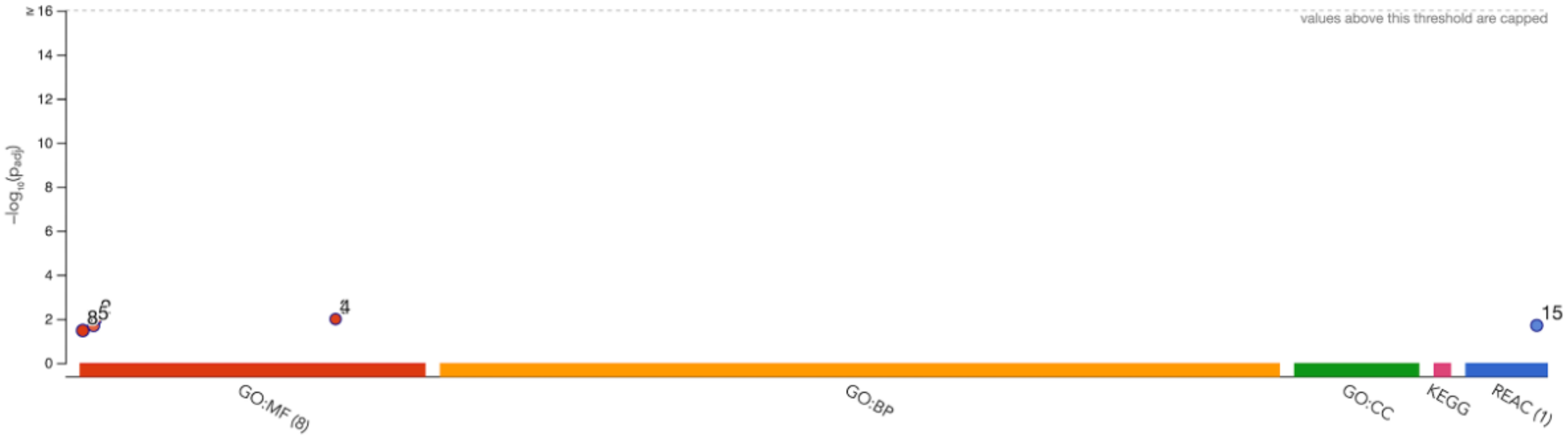

ID	Source	Term ID	Term Name	p_{adj} (BirThree (BCF...	p_{adj} (3.5KJPNv2 (B...	p_{adj} (BirThree (PLI...	p_{adj} (3.5KJPNv2 (P...
1	GO:MF	GO:0004984	olfactory receptor activity	8.168×10^{-9}	1.164×10^{-5}		
2	GO:MF	GO:0004047	aminomethyltransferase activity				1.026×10^{-2}
3	GO:MF	GO:0052839	inositol diphosphate tetrakisphosphate kinase acti...				1.026×10^{-2}
4	GO:MF	GO:0052836	inositol 5-diphosphate pentakisphosphate 5-kinas...				1.026×10^{-2}
5	GO:MF	GO:0003938	IMP dehydrogenase activity				2.049×10^{-2}
6	GO:MF	GO:0000829	inositol heptakisphosphate kinase activity				3.409×10^{-2}
7	GO:MF	GO:0000827	inositol-1,3,4,5,6-pentakisphosphate kinase activity				3.409×10^{-2}
8	GO:MF	GO:0000832	inositol hexakisphosphate 5-kinase activity				3.409×10^{-2}
9	GO:BP	GO:0050911	detection of chemical stimulus involved in sensory...	8.701×10^{-9}	4.796×10^{-5}		
10	GO:BP	GO:0007186	G protein-coupled receptor signaling pathway	2.569×10^{-4}	~~1.150×10^{-4}~~		~~1.000~~
11	KEGG	KEGG:04740	Olfactory transduction	1.145×10^{-9}	5.113×10^{-6}		
12	REAC	REAC:R-HS...	Expression and translocation of olfactory receptors	1.019×10^{-10}	7.502×10^{-6}		
13	REAC	REAC:R-HS...	Olfactory Signaling Pathway	1.217×10^{-10}	8.590×10^{-6}		
14	REAC	REAC:R-HS...	Sensory Perception	8.187×10^{-9}	2.088×10^{-4}		~~1.000~~
15	REAC	REAC:R-HS...	Synthesis of IPs in the nucleus				2.003×10^{-2}

version e111_eg58_p18_f463989d
date 10/16/2024, 12:11:24 PM
organism hsapiens

g:Profiler
