# Supplementary material for: Profiling of runs of homozygosity from whole-genome sequence data in Japanese biobank
Source: J Hum Genet. 2025 Apr 3;70(6):287–96. doi: 10.1038/s10038-025-01331-3 (PMC12058513; doi:10.1038/s10038-025-01331-3)
Supplement: Supplementary file 7 — Assessments on SNP-density effects [file 10038_2025_1331_MOESM7_ESM.pdf]

Figure S3A. Comparisons of mean number of ROH (NROH) per individual based on different minimal lengths after pruning: ROH segments > 1.5 Mb (left) and ROH segments > 100 Kb (right). Violin plot represents the distribution of mean number of ROH segments detected via BCFtools across individuals between 3.5KJPNv2, pruned 3.5KJPNv2 and BirThree datasets. Color schemes represent different datasets.

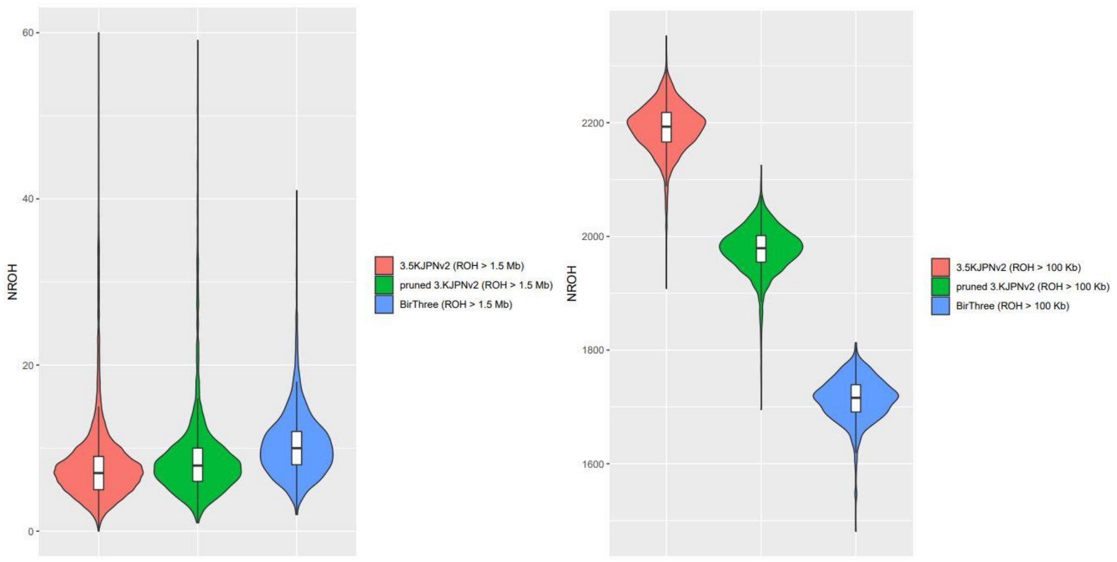

Figure S3B. Distribution of mean number of ROH (NROH) per individual who are commonly involved in both datasets, before preliminary removal of sites with higher Mendelian error rates in 3.5KJPNv2 dataset (minimal ROH length > 1.5 Mb) (N = 208 individuals). Violin plot represents the distribution of mean number of ROH segments longer than 1.5 Mb across overlapped individuals in both datasets before removing sites with higher Mendelian error rates. Color schemes represent specific conditions: genomic regions and parameter adjustments in selected tools.

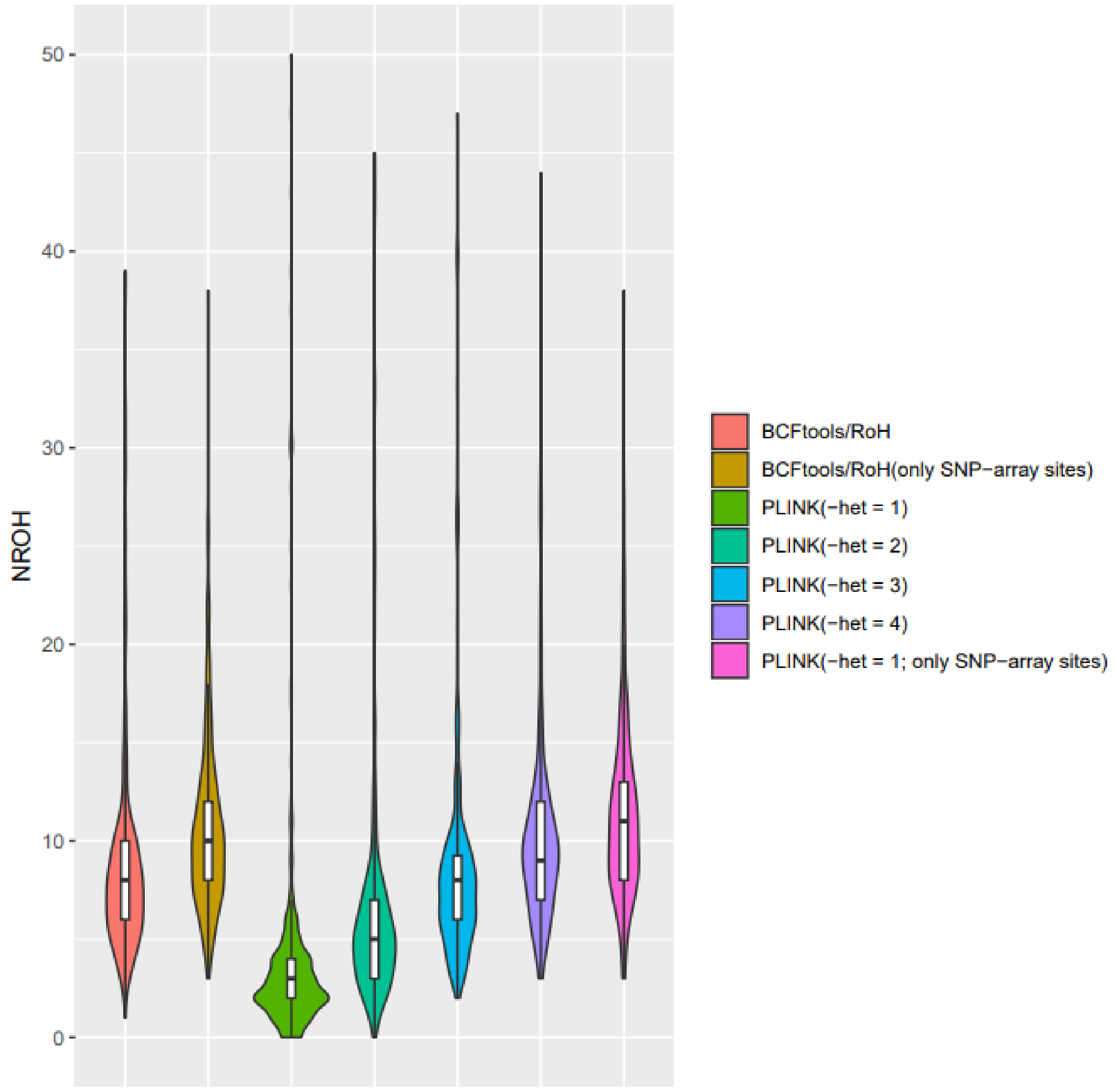

Figure S3C. Distribution of mean number of ROH (NROH) per individual who are commonly involved in both datasets, after preliminary removal of sites with higher Mendelian error rates in BirThree dataset (minimal ROH length > 1.5 Mb) (N = 208 individuals). Violin plot represents the distribution of mean number of ROH segments longer than 1.5 Mb across overlapped individuals in both datasets after removing sites with higher Mendelian error rates. Color schemes represent specific conditions: genomic regions and parameter adjustments in selected tools.

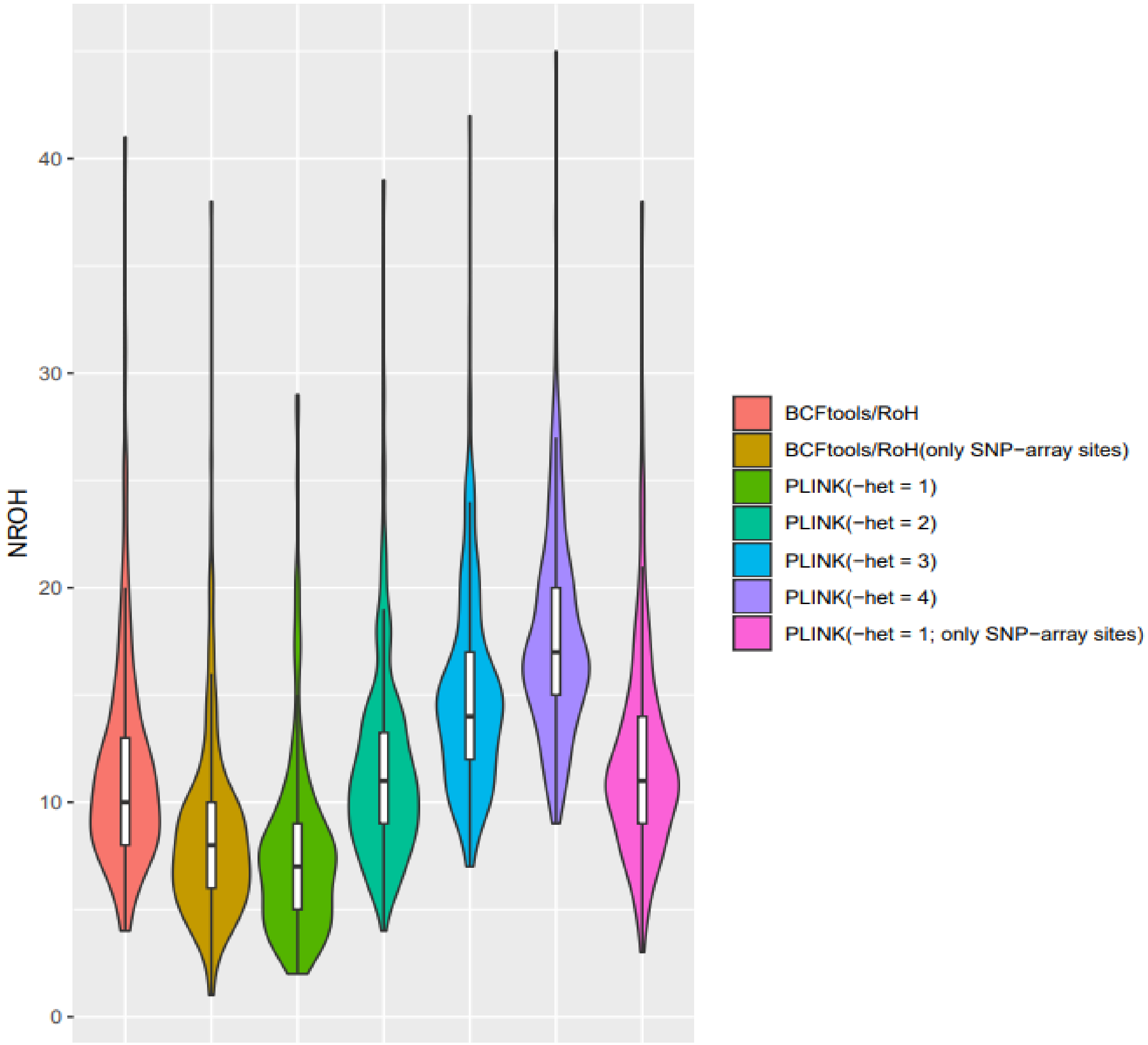

Figure S3D. Distribution of mean number of ROH (NROH) per individual, who are commonly involved in both datasets, before preliminary removal of sites with higher Mendelian error rates in 3.5KJPNv2 dataset (minimal ROH length > 100 Kb) (N = 208 individuals). Violin plot represents the distribution of mean number of ROH segments longer than 100 Kb across overlapped individuals in both datasets before removing sites with higher Mendelian error rates. Color schemes represent specific conditions: genomic regions and parameter adjustments in selected tools.

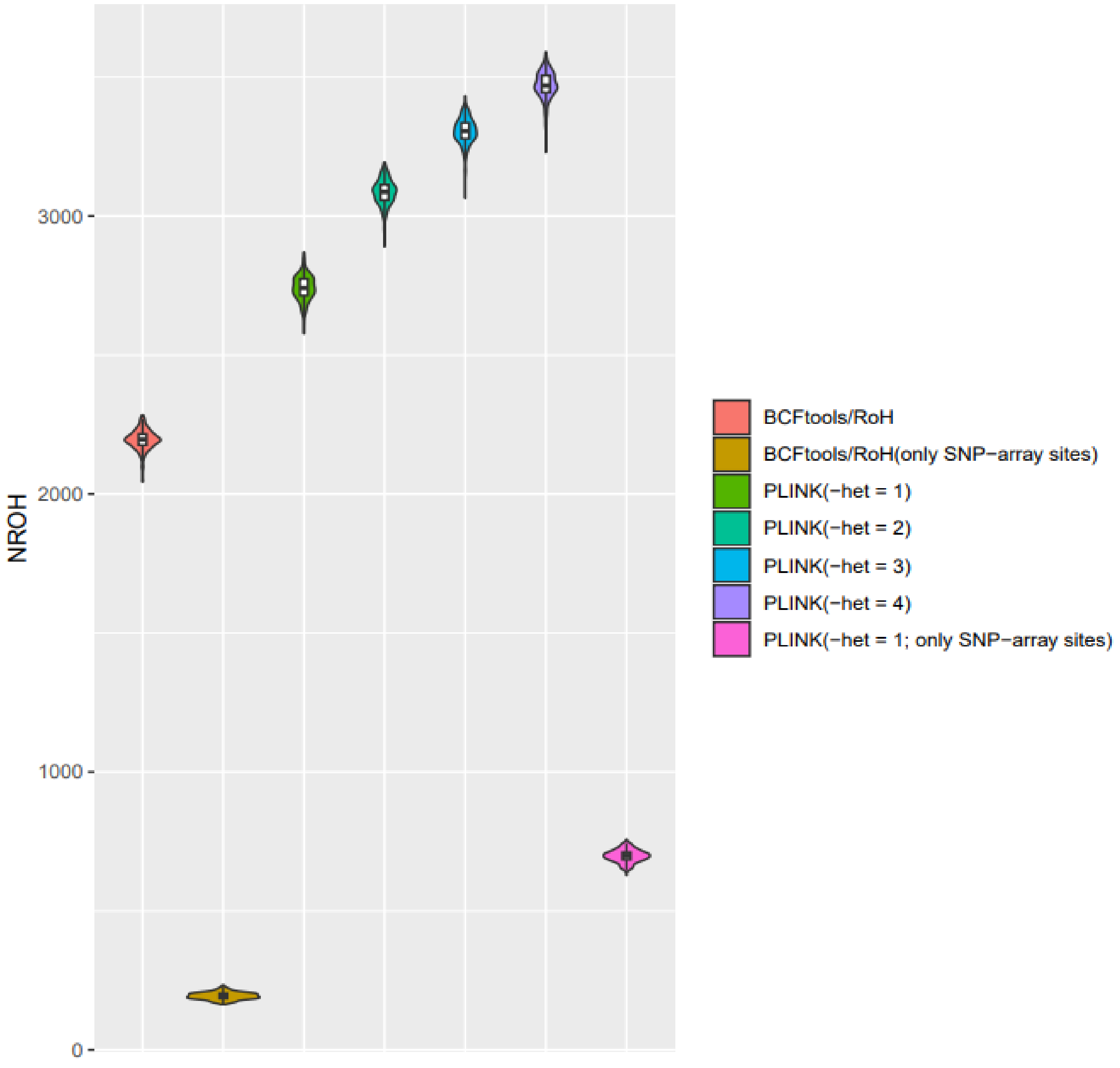

Figure S3E. Distribution of mean number of ROH (NROH) per individual, who are commonly involved in both datasets, after preliminary removal of sites with higher Mendelian error rates in BirThree dataset (minimal ROH length > 100 Kb) (N = 208 individuals). Violin plot represents the distribution of mean number of ROH segments longer than 100 Kb across overlapped individuals in both datasets after removing sites with higher Mendelian error rates. Color schemes represent specific conditions: genomic regions and parameter adjustments in selected tools.

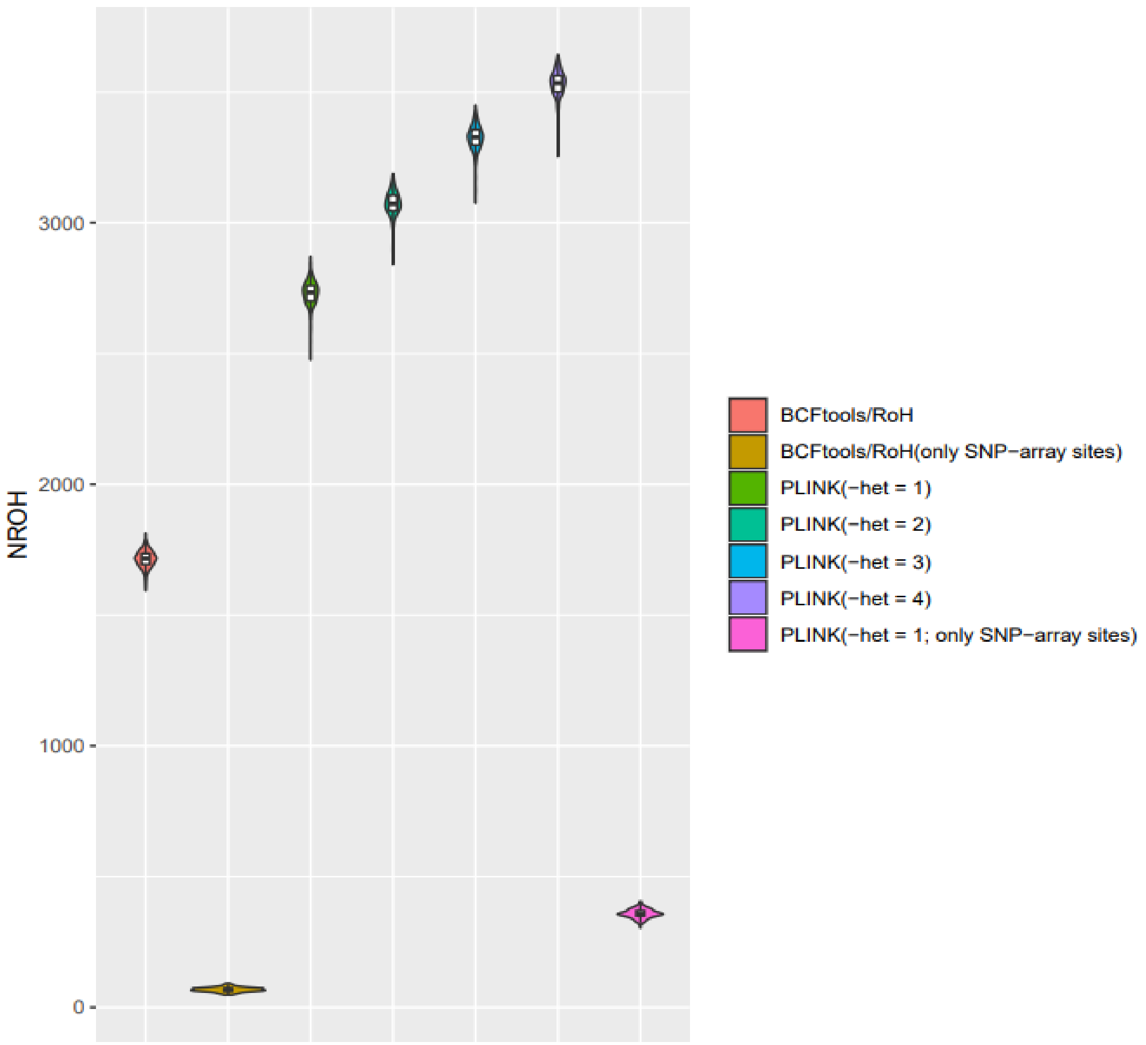

Figure S3F. Distribution of mean number of ROH (NROH) per individual in 3.5KJPNv2 dataset (minimal ROH length > 100 KB). Violin plot represents the distribution of mean number of ROH segments longer than 100 KB across individuals in the 3.5KJPNv2 dataset. Color schemes represent specific conditions: genomic regions and parameter adjustments in selected tools.

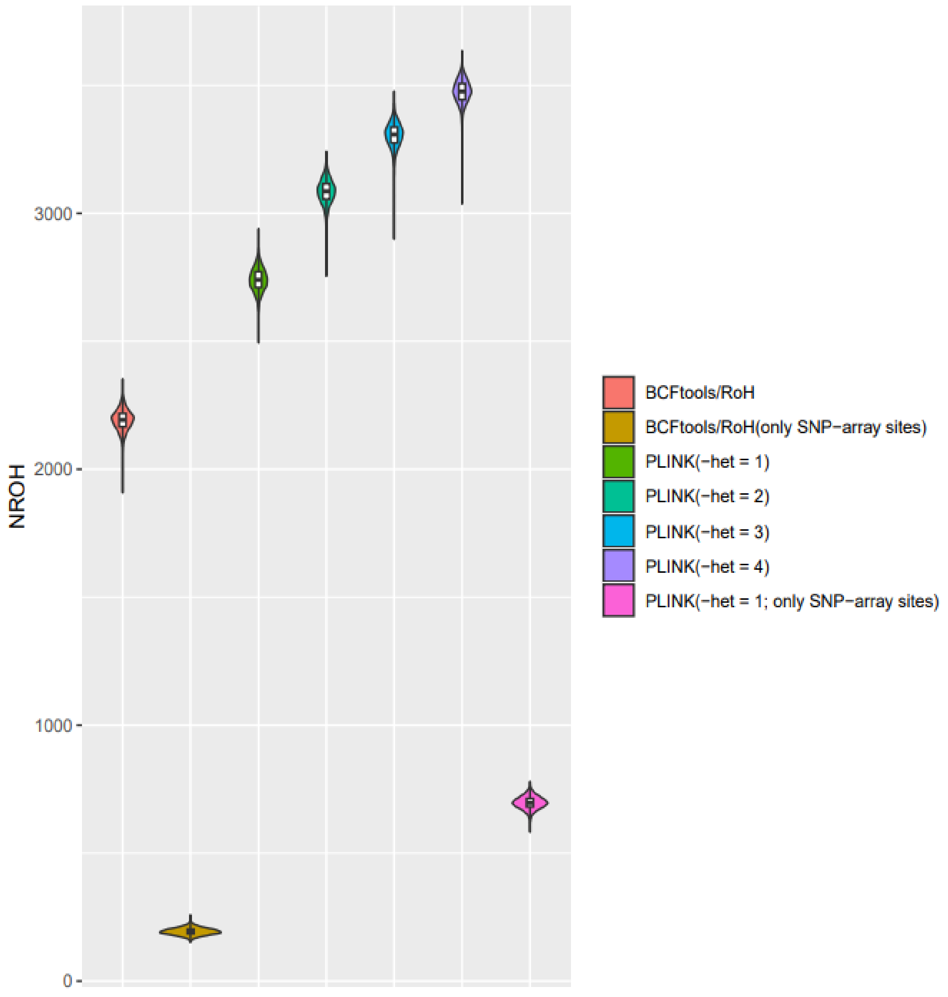

Figure S3G. Distribution of mean number of ROH (NROH) per individual in BirThree dataset (minimal ROH length > 100 KB). Violin plot represents the distribution of mean number of ROH segments longer than 100 KB across individuals in BirThree dataset. Color schemes represent specific conditions: genomic regions and parameter adjustments in selected

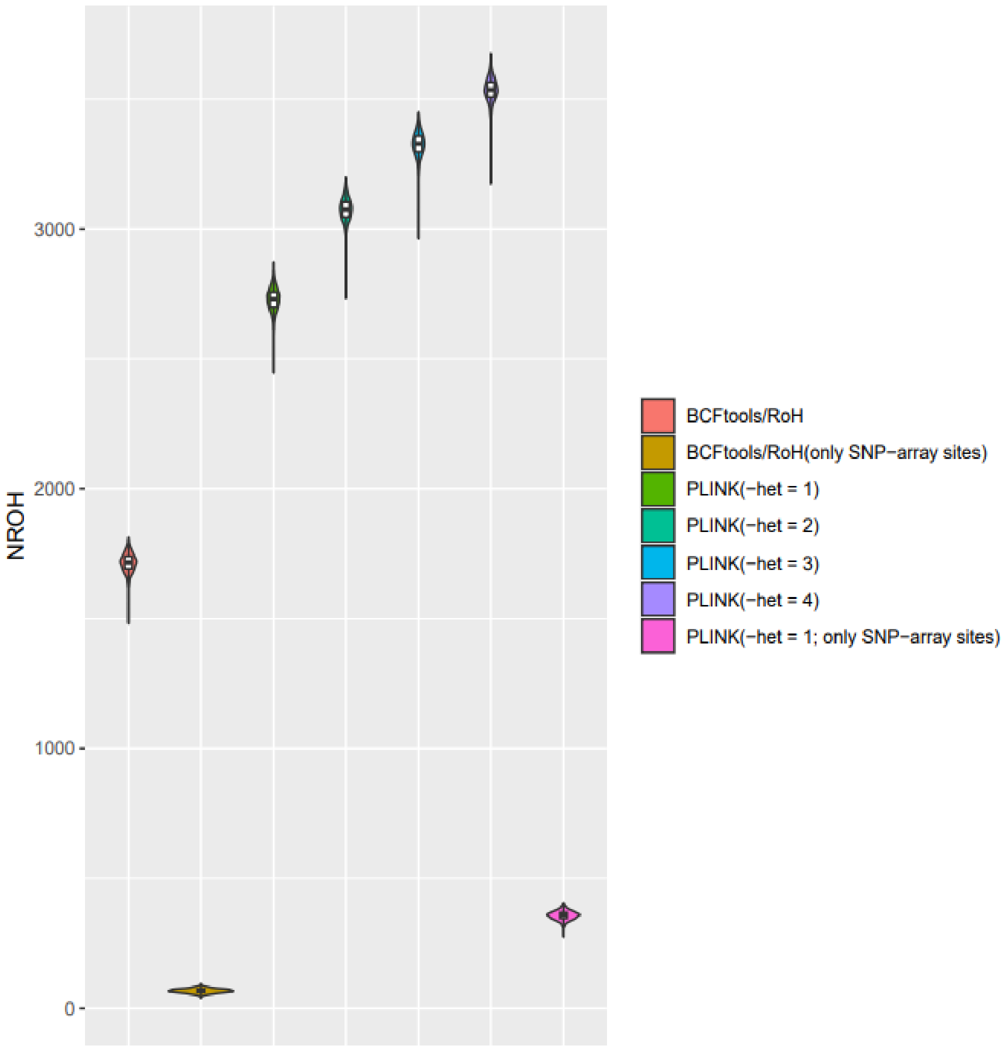

tools.
