# Supplementary material for: Profiling of runs of homozygosity from whole-genome sequence data in Japanese biobank
Source: J Hum Genet. 2025 Apr 3;70(6):287–96. doi: 10.1038/s10038-025-01331-3 (PMC12058513; doi:10.1038/s10038-025-01331-3)

Figure S4A. Stratification of ROH segments by six different intervals in 3.5KJPNv2 dataset (Linear scale). Line graphs show the distribution of mean number of ROH segments in each interval across individuals in the 3.5KJPNv2 dataset. Color schemes represent specific conditions: genomic regions and parameter adjustments in selected tools.

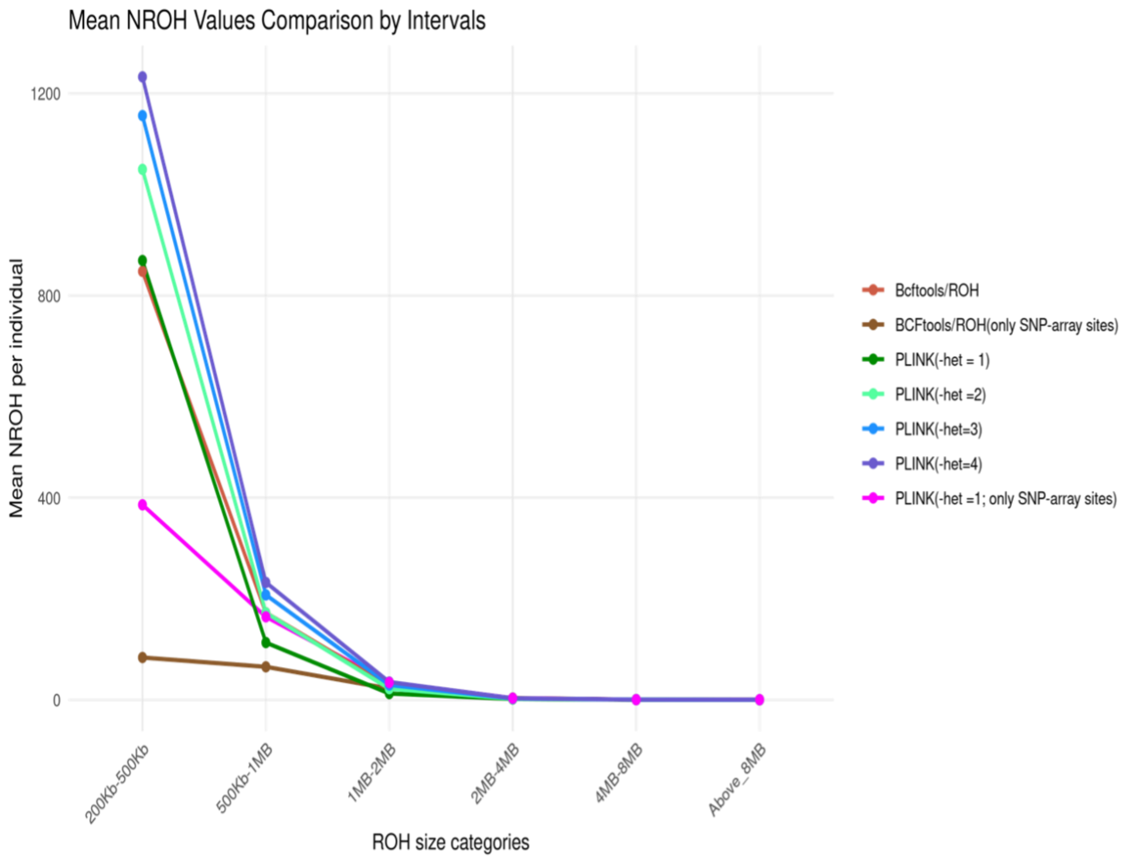

Figure S4B. Stratification of ROH segments by six different intervals in BirThree dataset (Linear scale). Line graphs show the distribution of mean number of ROH segments in each interval across individuals in the BirThree dataset. Color schemes represent specific conditions: genomic regions and parameter adjustments in selected tools.

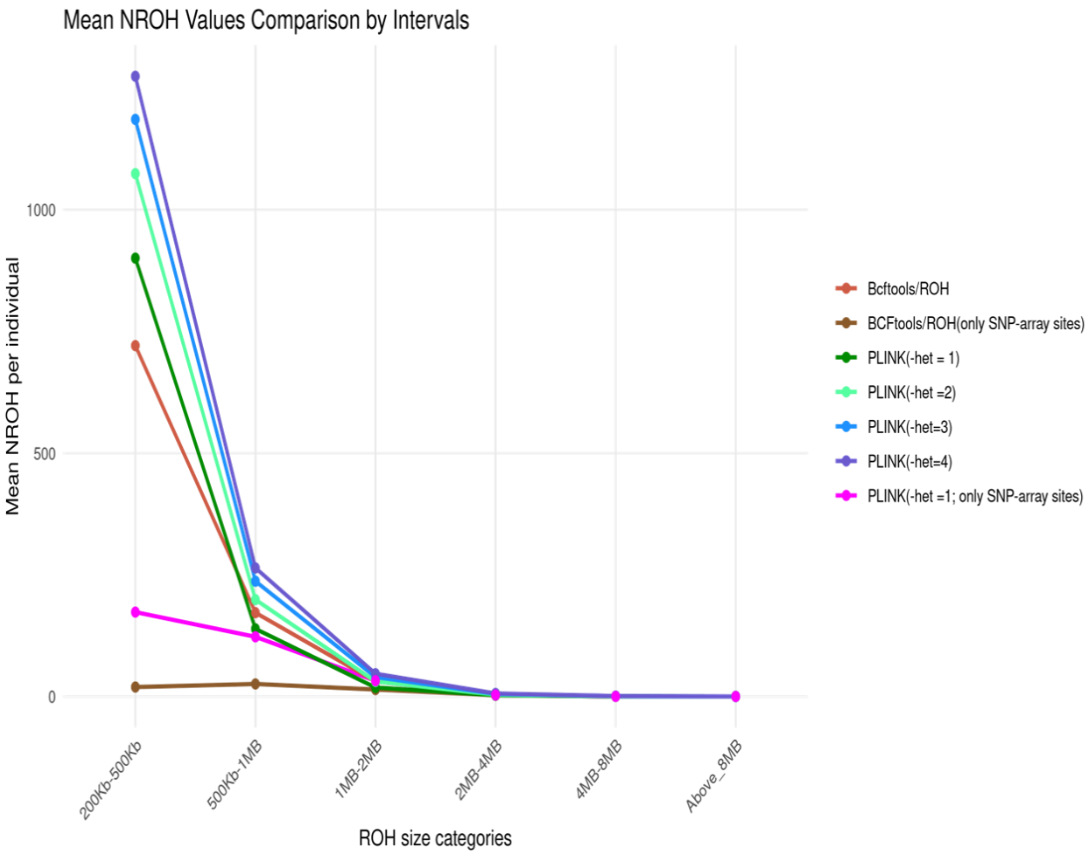

Figure S4C. Stratification of ROH segments by six different intervals in 3.5KJPNv2 dataset (Log-scale). Line graphs show the distribution of mean number of ROH segments in each interval across individuals in the 3.5KJPNv2 dataset. Color schemes represent specific conditions: genomic regions and parameter adjustments in selected tools.

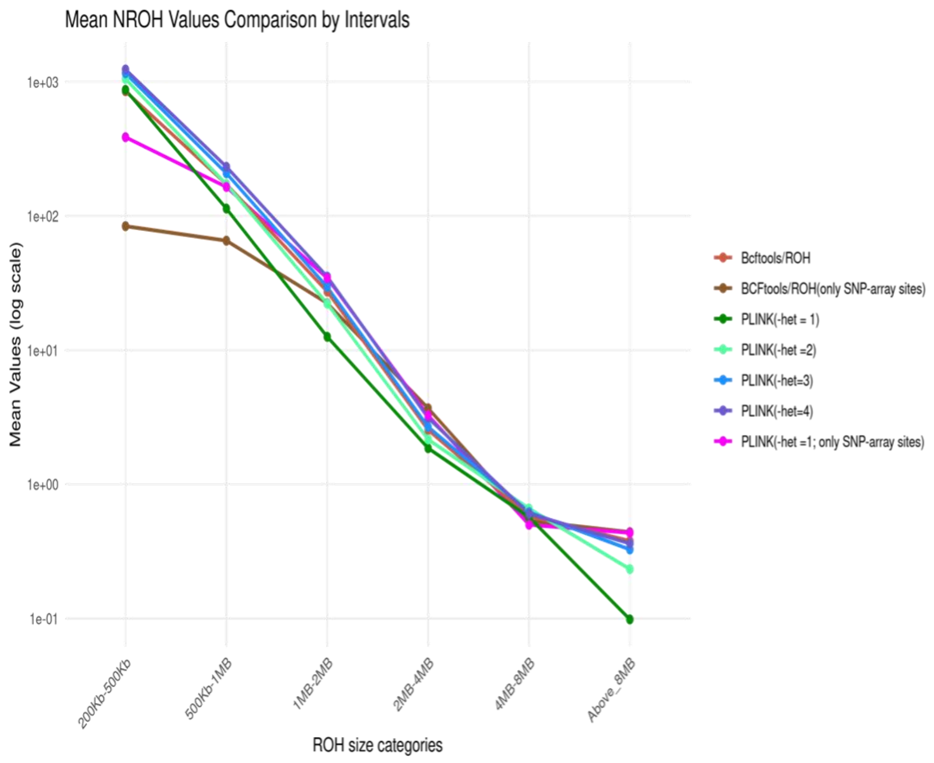

Figure S4D. Stratification of ROH segments by six different intervals in BirThree dataset (Log-scale). Line graphs show the distribution of mean number of ROH segments in each interval across individuals in the BirThree dataset. Color schemes represent specific conditions: genomic regions and parameter adjustments in selected tools.

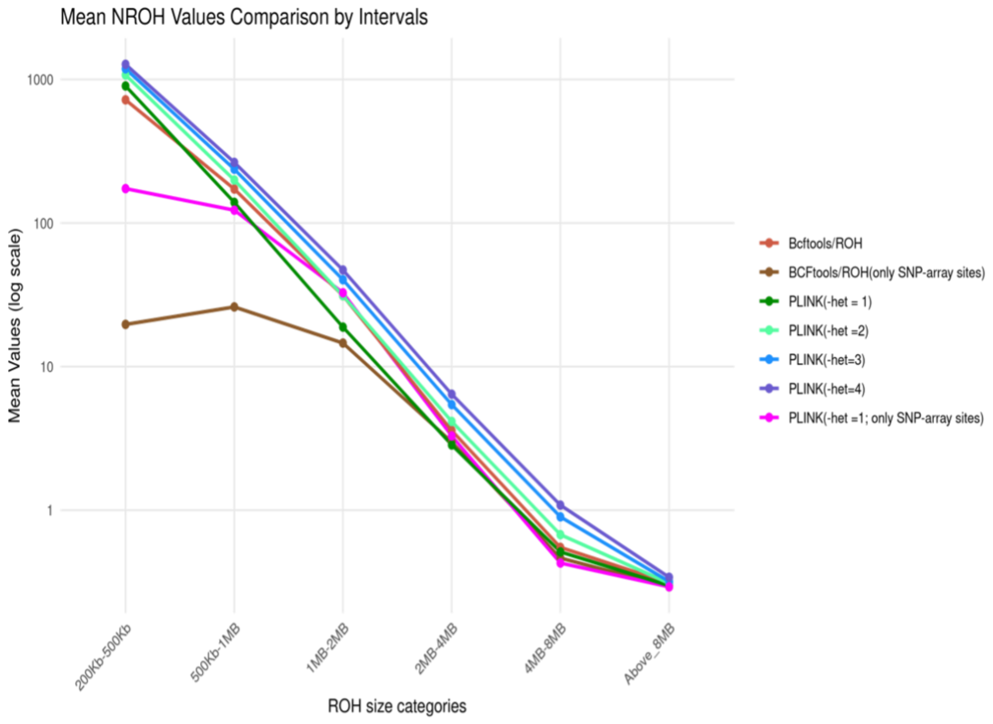

Figure S4E. Distribution of ROH segments by size, detected by BCFtools in 3.5KJPNv2 dataset. Histogram illustrating the frequency of ROH segment lengths within specific intervals. Color schemes represent specific genomic regions.

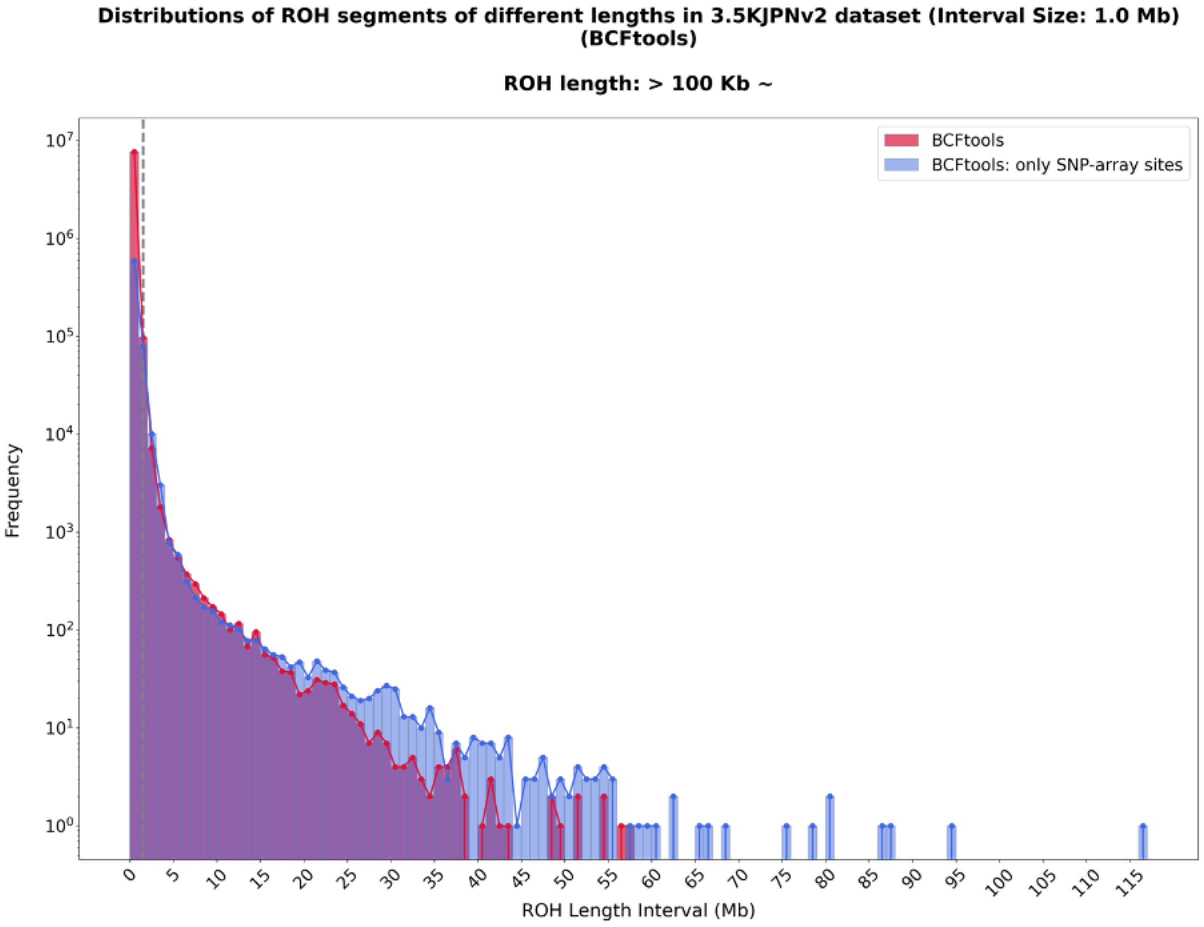

Figure S4F. Distribution of ROH segments by size, detected by BCFtools in BirThree dataset. Histogram illustrating the frequency of ROH segment lengths within specific intervals. Color schemes represent specific genomic regions.

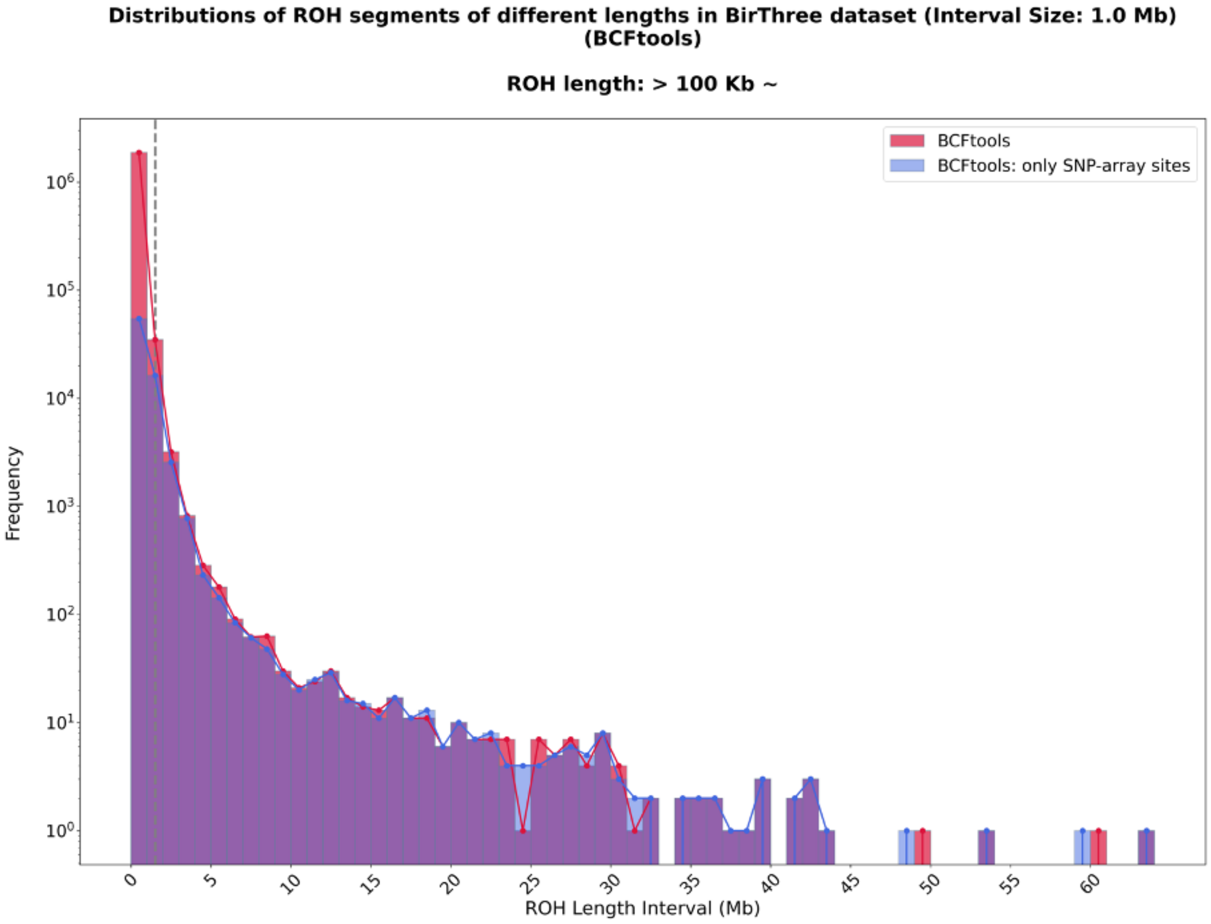

Figure S4G. Distribution of ROH segments by size, detected by PLINK in 3.5KJPNv2 dataset. Histogram illustrating the frequency of ROH segment lengths within specific intervals. Color schemes represent specific genomic regions and parameter adjustments.

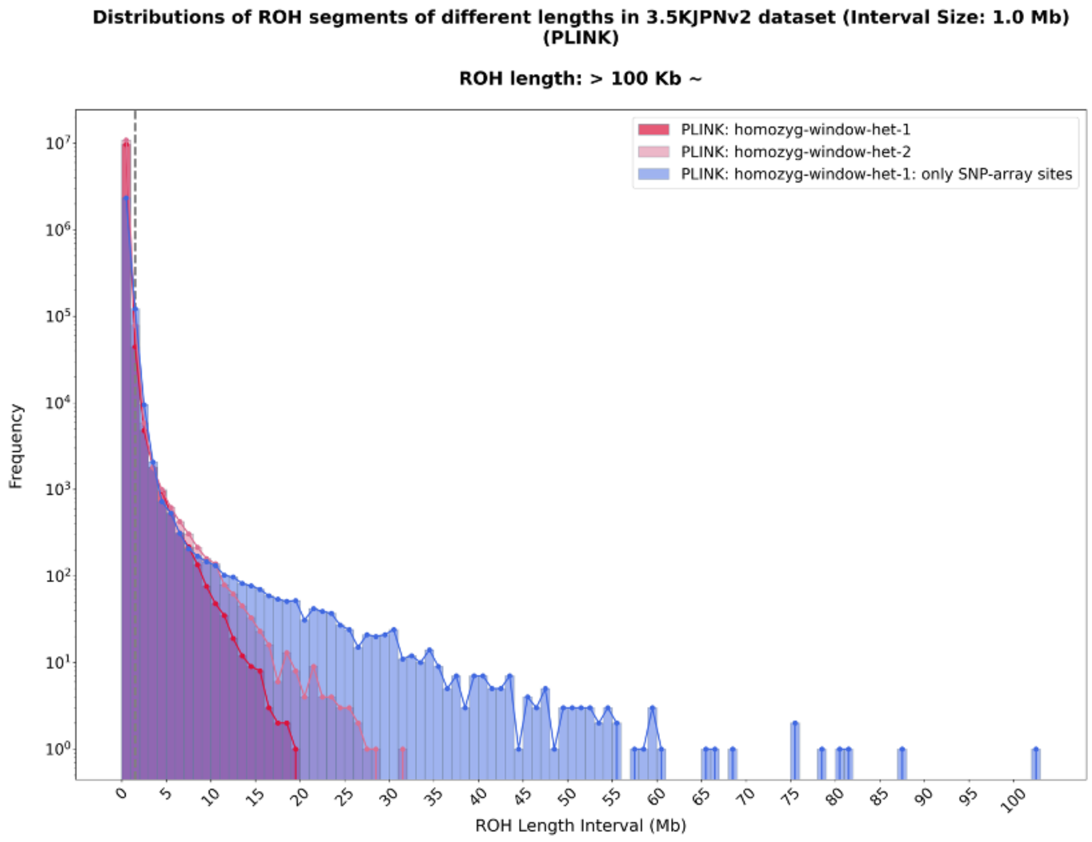

Figure S4H. Distribution of ROH segments by size, detected by PLINK in BirThree dataset. Histogram illustrating the frequency of ROH segment lengths within specific intervals. Color schemes represent specific genomic regions and parameter adjustments.

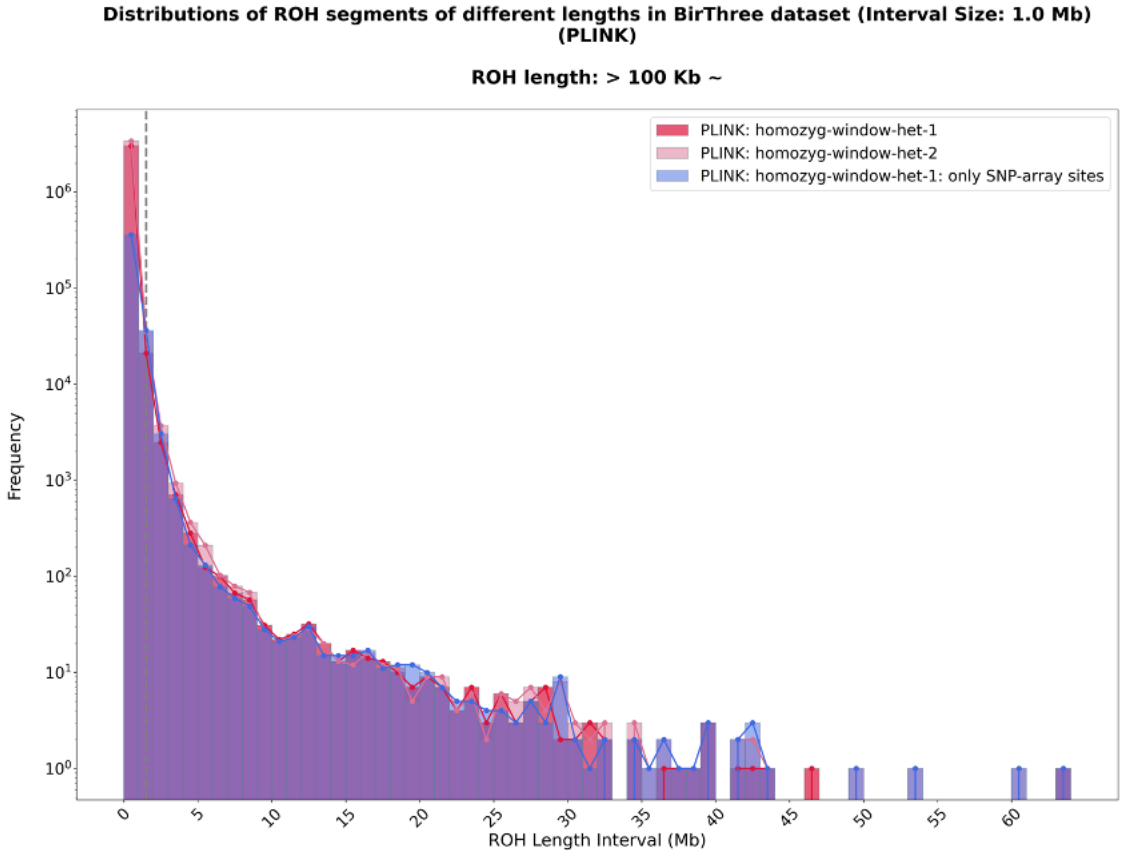

Supplement: Supplementary file 8 — Stratification of ROHs based on different ROH length intervals [file 10038_2025_1331_MOESM8_ESM.pdf]
